# Efficacy of a World Health Organization–Guided Self-Help Intervention for Reducing Psychological Distress in Afghan Refugees: Randomized Controlled Trial

**DOI:** 10.2196/89928

**Published:** 2026-05-20

**Authors:** Angela Nickerson, Gulsah Kurt, Philippa Specker, Anna Camilleri, Dessy Susanty, Rizka Argadianti, David Keegan, Randy Nandyatama, Atika Yuanita, Angga Putra Reynady Hermawan, Richard A Bryant, Meaghan O'Donnell, Mahdi Rafei, Rahmatullah Bayani, Belinda J Liddell

**Affiliations:** 1 School of Psychology, UNSW Sydney, Sydney, New South Wales, 2052, Australia, +61 2 9065 7755; 2 SUAKA Indonesian Civil Society Association for Refugee Rights Protection, Jakarta, Indonesia; 3 Excelsia College, Sydney, New South Wales, Australia; 4 Universitas Gadjah Mada, Yogyakarta, Yogyakarta, Indonesia; 5 Phoenix Australia, Department of Psychiatry, The University of Melbourne, Melbourne, Victoria, Australia; 6 Refugee Learning Nest, Bogor, Indonesia; 7 School of Psychological Sciences, University of Newcastle Australia, Newcastle, New South Wales, Australia

**Keywords:** refugees, Doing What Matters, World Health Organization, psychological distress, functioning

## Abstract

**Background:**

Common mental health disorders are highly prevalent among refugees. There is an urgent need to address the mental health burden in this population.

**Objective:**

This study tested the efficacy of an individually supported self-help stress-management intervention developed by the World Health Organization—Doing What Matters in Times of Stress (DWM)—in reducing psychological distress and improving functioning among refugees in Indonesia, a major transit country in the Asia-Pacific region.

**Methods:**

A single-blind randomized controlled trial with 303 Farsi-speaking refugees was conducted between June 2024 and June 2025. Participants with moderate to high psychological distress (Kessler Psychological Distress Scale [K10] score≥20) were randomly allocated to the facilitator-guided individual DWM condition (n=202) or a repeated assessment control condition (n=101). The primary outcome was psychological distress (K10 score) at the posttreatment assessment. Secondary outcomes were posttraumatic stress disorder symptoms, functional impairment, social functioning, and personally identified problems.

**Results:**

Intent-to-treat analysis indicated that participants in the DWM condition showed greater reductions in K10 scores than those in the repeated assessment control condition (posttreatment: *β*=−.563, SE=0.124; *P*<.001; Cohen *d*=0.56; 1-month follow-up: *β*=−.447, SE=0.140; *P*=.002; Cohen *d*=0.45). Similarly, those participants in the DWM condition reported greater improvements in posttraumatic stress disorder symptoms, well-being, social functioning, functional impairment, and personally identified psychological problems. No serious adverse events were reported.

**Conclusions:**

The findings provide the first evidence for the effectiveness of DWM in reducing psychological distress and improving overall functioning among urban refugees living in a transit setting. Individually supported self-help interventions such as DWM mayoffer an effective, feasible, and scalable approach to improving mental health for refugees.

## Introduction

In 2024, one out of every 67 people in the world was forcibly displaced [1]. Exposure to persecution and war puts refugees at high risk of mental disorders, with estimated prevalence rates of 32% for both posttraumatic stress disorder (PTSD) and depression among refugees and asylum seekers [2]. Over three-quarters of refugees worldwide are displaced to low- and middle-income countries (LMICs) [1,3]. For refugees in LMICs, resources are often scarce, and living conditions can be precarious. Lack of legal status often limits access to financial support, housing, and essential services and increases vulnerability to discrimination, violence, and exploitation [4-6]. There is also often a paucity of refugee support services and local health care to meet refugees’ mental health needs in LMICs [3]. This creates a critical gap between the significant mental health burden held by refugees and the resources available to promote psychological recovery.

There is a need for low-cost, scalable, and sustainable approaches to reduce psychological symptoms among refugees in LMICs. Interventions that require mental health specialists, intensive training and supervision, and face-to-face delivery over several months may not be feasible in low-resource settings [7]. To address this problem, the World Health Organization (WHO) has developed a suite of scalable interventions to alleviate mental burden in humanitarian and low-resource settings [8,9]. One of these—Doing What Matters in Times of Stress (DWM) [10]—is a predominantly self-help intervention, where individuals are provided with a workbook and audio files in their native language and receive short phone calls from nonspecialists to support their engagement with the materials. Although some trials have been conducted with DWM in combination with other interventions [11], the only study to date testing DWM as a standalone intervention was undertaken with Syrian refugees and Turkish nationals in Türkiye [12]. While this trial found promising evidence for a reduction in PTSD symptoms in Syrian refugees and depression symptoms in Turkish nationals, it was underpowered to detect group differences. Other studies have tested Self-Help Plus, DWM’s group-based alternative, demonstrating its efficacy in preventing mental disorders among refugees with subclinical symptoms in Western Europe and Türkiye [13,14] and reducing psychological distress in female Sudanese refugees in a camp setting in Uganda [15]. To date, however, there has not been a fully powered trial investigating the efficacy of the DWM program in reducing psychological distress among refugees living in LMIC. This is an important gap, as there is evidence that individually supported interventions may confer greater benefit than their group-based counterparts [16]. Further, self-help approaches with individual guidance may overcome important implementation barriers for refugees in transit settings, including stigma, logistical challenges, and the need for individual tailoring of content and delivery [17].

In this study, we conducted a randomized controlled trial evaluating the efficacy of DWM in reducing psychological distress among Afghan refugees living in prolonged displacement in the greater Jakarta area in Indonesia. Indonesia is a key transit country for refugees in the Asia-Pacific, hosting approximately 12,000 refugees [18]. Like many transit countries, Indonesia is a nonsignatory to the United Nations Refugee Convention and its Protocol and does not permanently resettle refugees. In this context (as in many other transit settings), refugees are exposed to a myriad of daily stressors and have access to few formal services [19]. This means that the conditions of safety and security that are important for mental health recovery following adversity are notably absent [20], and that it is incumbent on refugee communities and refugee-led organizations to provide necessary supports to displaced individuals. There is thus an urgent need to evaluate the efficacy of scalable self-help approaches that can be implemented by refugee communities to reduce psychological distress. The objective of this study was to evaluate the efficacy of DWM in reducing psychological distress (primary outcome), PTSD symptoms, personally identified psychological problems, improving well-being and functioning among Afghan refugees living in Indonesia, as well as to evaluate the clinical significance of associated symptom change. We hypothesized that, compared to a repeated assessment control (RAC) group, refugees who received DWM would show greater improvements in psychological distress at posttreatment (primary outcome) and 1-month follow-up, as well as greater improvements in PTSD symptoms, functional impairment, personally identified psychological problems, social functioning, and well-being.

## Methods

### Design and Setting

We conducted a 2-arm single-blind randomized controlled trial with refugees from Afghanistan living in Indonesia. Indonesia hosts approximately 12,000 refugees, with approximately half of these living in urban settings in the greater Jakarta area [21]. While all refugees ostensibly live in Indonesia temporarily as they await resettlement to other countries, in actuality, this waiting period is often indefinite due to limited resettlement places [22]. During this protracted displacement, refugees in Indonesia do not have the right to work or to reunite with their families. They have little access to education and government-sponsored services, with the limited support available to refugees being provided by nongovernment and refugee-led community-based organizations [23]. Approximately half of the refugees in Indonesia originate from Afghanistan [18]. Refugees from Afghanistan have experienced decades of armed conflict, political instability, and human rights abuses, with a renewed wave of mass displacement occurring following the Taliban’s return to power in 2021, which sharply curtailed civil liberties, education, and employment [24]. The humanitarian crisis in Afghanistan is considered one of the most complex crises of our time, given the lack of resolution after more than 2 decades of international intervention. Many Afghan refugees have also been exposed to prolonged premigratory trauma and perilous migration journeys, including violence, loss, and extended periods of insecurity in transit or displacement settings, contributing to high levels of psychological distress on arrival in host countries [25].

We elected to undertake this study in a digital format (ie, recruitment via social media and assessment and delivery of DWM materials via Zoom [Zoom Video Communications] calls) to increase access to the intervention in a population that was geographically dispersed around the greater Jakarta area. This methodology was considered feasible given (1) widespread internet access in Indonesia [26], (2) the successful implementation of digital procedures in our previous longitudinal study of over 1300 refugees living in Indonesia [19], and (3) reports from our study partners that smartphone use was ubiquitous among refugees in Indonesia, consistent with evidence that refugees commonly use digital methods to stay in contact with family in the country of origin or displaced in other settings [26,27]. In recognition of the cost of accessing reliable internet in Indonesia, participants in both conditions were provided with an internet allowance.

### Ethical Considerations

The trial protocol (Multimedia Appendix 1) was prospectively registered at the Australian and New Zealand Clinical Trials Registry (ACTRN12624000609550) and approved by the UNSW Sydney Human Research Ethics Committee in Australia (iRECS4937) and the Badan Riset dan Inovasi Nasional in Indonesia (25032024000003 and 07052025008596). This superiority trial was conducted in collaboration with the Universitas Gadjah Mada, the Indonesian Civil Society Association for Refugee Rights Protection (SUAKA), and Refugee Learning Nest. The CONSORT-eHealth (Consolidated Standards of Reporting Trials of Electronic and Mobile Health Applications and Online Telehealth) checklist is available in Checklist 1. Participants provided informed consent and were able to opt out of the study at any time without penalty. After each assessment, participants received a US $6 digital grocery voucher as compensation and an internet allowance. Participants in the RAC group were offered access to the self-help version of the DWM intervention after completing the assessments, ensuring that no participants were denied the opportunity to receive the intervention. This study complied with informed consent guidelines and adhered to local, national, regional, and international law and regulations regarding protection of personal information, privacy, and human rights

### Procedures

Participants were recruited online through social media, advertisements on the study website, and flyers distributed to organizations supporting refugees in the Greater Jakarta area in Indonesia (Jakarta, Bogor, Depok, Tangerang, and Bekasi). Interested participants completed an online registration form that included study information, consent to a screening call with a trained assessor, the Kessler Psychological Distress Scale (K10) [28], an initial eligibility question confirming refugee status in Indonesia, and demographic questions. Those who consented and met the initial eligibility criteria were contacted by a trained assessor for an online screening call. During this call, information was collected on access to a digital device and internet connection, current treatment, suicide risk, and severe cognitive impairment. The K10 was also readministered to verify self-reported responses. Participants who met the full eligibility criteria proceeded to the baseline assessment prior to randomization. All assessments were conducted online by trained assessors.

### Inclusion and Exclusion Criteria

The inclusion criteria were (1) being aged 18 years or older, (2) being from a refugee background (ie, living in Indonesia and having registered with the United Nations High Commissioner for Refugees (UNHCR) or intending to register with the UNHCR), (3) residing in Greater Jakarta area (Jakarta, Bogor, Depok, Tangerang, or Bekasi), (4) ability to speak and read Farsi, (5) elevated psychological distress (≥20 on K10 [28]), and (6) providing written or verbal informed consent before entering the study. The Farsi language was selected for this study, as it represented a common language for Afghan refugees from Dari, Hazaragi, and Pashto-speaking backgrounds. Exclusion criteria included (1) acute medical condition, (2) imminent suicide risk or expressed acute needs or protection risk (ie, being at acute risk of domestic and/or sexual violence), (3) severe mental disorder (psychotic disorder or substance dependence), (4) severe cognitive impairment (eg, severe intellectual disability or dementia), (5) concurrent psychological treatment (eg, concurrently receiving psychological services from a psychologist, counselor, or mental health professional), (6) no access to smartphone or internet connection, and (7) having another household member who had already applied to take part in the study. Those excluded based on suicide risk, severe mental disorder, or severe cognitive impairment were referred to the available general or specialist services.

### Randomization and Masking

Eligible participants were randomly assigned to DWM or the RAC condition at a 2:1 ratio. This ratio was implemented to allow for the investigation of mechanisms of action within the DWM condition (which will be detailed in future reports). Randomization was conducted by a researcher who was not involved in the delivery of the intervention. Randomization was performed using a computerized software (REDCap [Research Electronic Data Capture]; Vanderbilt University) [29,30]. Assessors were masked to treatment condition allocation. Assessors were managed separately from other members of the research team and did not interact with the DWM facilitators. Participants were instructed not to share their condition allocation with the assessors to ensure objectivity of the assessments.

### Conditions

#### DWM Intervention

DWM is a self-help stress management program developed by the WHO [10] to teach individuals evidence-based strategies for reducing psychological distress. The program consists of an illustrated booklet with 5 sections and accompanying audio recordings, introducing techniques based on acceptance and commitment therapy [10,31], such as mindful attention, acceptance of difficult thoughts and feelings, engaging in valued actions, and practicing kindness toward oneself and others. In this study, the Farsi version of DWM was delivered over 5 weeks via weekly one-to-one calls with a trained facilitator. The original Farsi materials developed by the WHO [10] were used in this study without modification to preserve fidelity and replicability. Participants in the DWM condition received the materials via WhatsApp message and then commenced the program with an introduction call from their assigned facilitator. In this call, the facilitators explained the DWM program, outlined their roles, discussed privacy and confidentiality, and identified participants’ goals for participating in the program. This was followed by weekly lesson calls (via Zoom), during which facilitators reviewed and practiced each strategy with the participants, answered any questions, and discussed any challenges they encountered while using the materials. At the end of each call, participants and their facilitators collaboratively completed the daily action plan to schedule between-session practices and to identify potential challenges and solutions to help participants stay on track. Each lesson call lasted 15 to 30 minutes and was recorded online. We randomly selected 10% (n=120) of the recorded calls for review, ensuring that each lesson was equally represented. These recordings were evaluated during the implementation phase to assess both intervention fidelity and facilitator competence. Two facilitator team leaders watched 36 recorded calls together to establish consistency in their evaluations and then individually reviewed 42 calls. Treatment fidelity was evaluated using a 5-item checklist (yes or no), assessing the completion of the required components of each lesson (eg, provide a summary of the lesson, practice the strategy, and reflect on the practice experience). Facilitator competence was assessed using a version of the Enhancing Assessment of Common Therapeutic Factors scale [32] adapted for this study (Multimedia Appendix 2). For the Enhancing Assessment of Common Therapeutic Factors ratings, 2 facilitator team leaders watched each of the videos and rated each facilitator on basic helping skills (eg, active listening, demonstrating empathy, and promoting confidentiality).

#### RAC Condition

A RAC condition was implemented in this study to evaluate the relative benefit of DWM over and above supportive contact with refugee community members. This condition was selected as (1) we were interested in investigating the efficacy of DWM in contexts where there are limited supports available; the absence of formal services for refugees in Indonesia meant that a care-as-usual control condition was not meaningful; and (2) the substantial assessment schedule in this study (4 assessments administered via Zoom by refugee community members) allows us to, at least partly, control for potential benefits of social contact on psychological distress [33]. While the RAC afforded an ecologically valid control condition that allows us to address study aims, this design means we are unable to make inferences regarding the superiority of DWM compared to other methods, nor to completely rule out that study effects were driven by supportive contact (as those in the DWM condition received a greater dosage of this).

Participants in the RAC condition were informed of their group allocation via WhatsApp message, provided with information about their upcoming assessments with the assessors, and notified that they would receive access to the DWM materials upon completing assessments. Once they completed the assessments, they were provided with a link to access the DWM booklet and audio recordings. A service directory including available services supporting refugees in Indonesia was available to those in both conditions who requested it upon completing the program.

All facilitators (women: 4/8, 50%) and assessors (women: 5/10, 50%) were members of the refugee community in Indonesia. They were selected based on the following criteria: (1) aged 18 years or older, (2) from a refugee background (ie, living in Indonesia and having registered with the UNHCR or intending to register with the UNHCR), (3) ability to speak and read Farsi and English, and (4) prior experience in supporting refugees. They had no prior formal mental health training. Both facilitators and assessors completed 6 days of in-person training for their respective roles. The training was delivered by 2 doctoral-level clinical psychologists with extensive experience working with displaced communities and an Indonesia-based clinical psychologist with detailed contextual knowledge. Training was supported by an Indonesia-based community engagement officer and a psychology graduate, both had experience in working with refugee communities. Core training elements included basic helping skills, managing participant distress during calls, and suicide risk assessment. The training also covered basic principles of remote intervention delivery and conducting assessments. In addition, the facilitators were trained in the DWM protocol, while the assessors were trained in standardized assessment techniques and research principles. Following the classroom training and role-plays, both facilitators and assessors completed 2 rounds of experiential learning through piloting the intervention and assessments with practice participants. During the pilot phase, they received individualized and group supervision to ensure that they demonstrated sufficient skills for full project delivery.

During the trial, facilitators received weekly supervision from an external doctoral-level clinical psychologist and an Indonesia-based clinical psychologist. The main topics covered included clarifying the privacy and purpose of each DWM strategy, managing difficult situations (eg, unresponsive or less motivated participants), refining skills for lesson delivery, and discussing self-care strategies. Assessors also received weekly supervision from the same external doctoral-level clinical psychologist and an experienced psychology graduate, focusing on standardized administration of measures, assessment fidelity, managing difficult situations on calls, and suicide risk assessments.

### Assessment

#### Overview

Participants were assessed at 4 time points: baseline, mid-treatment (3 weeks after randomization or upon completion of Lesson 3 in the DWM condition), posttreatment (6 weeks after randomization or after completing Lesson 5 in DWM), and 1-month follow-up (9 weeks after randomization or 1 month after completing Lesson 5 in DWM).

#### Measures

To determine eligibility, we used the total score of the K10 [28], a 10-item measure of anxiety and depression symptoms, with total scores ranging from 0 to 50. Participants were eligible to take part in the study if they reached the threshold of moderate to high psychological distress, which is represented by a cutoff score of 20 on the K10 [34]. A cutoff of 20 was selected to recruit participants with moderate to severe levels of psychological distress. This score was derived from population-based surveys that specify that a cutoff of 20 represents the likelihood of having a mild mental disorder [34]. Further, this approach is consistent with other research investigating the efficacy of scalable interventions that have used an inclusion cutoff of 20 on the K10 to represent moderate to severe psychological distress [35].

Suicide risk was assessed with three questions adapted from the Problem Management Plus manual [8], which assessed whether the participant (1) had serious thoughts or a plan to end their life over the past month, (2) had taken any actions to end their life over the past month, or (3) planned to end their life in the next 2 weeks. Participants were ineligible to take part in this study if they reported a suicide attempt in the past month or a plan to end their life in the next 2 weeks.

Severe mental disorder and cognitive impairment were again assessed using the Problem Management Plus observation checklist completed by the assessors. Participants were evaluated based on their ability to understand and follow the conversation (indicative of cognitive impairment) and whether they appeared disconnected from reality or exhibited unusual behaviors (eg, disorganized speech and confusion).

The primary outcome for this study was psychological distress, which was assessed using the K10 (α=.83) [28]. Secondary outcomes for this study were assessed as follows. Symptoms of PTSD were measured using the PTSD Checklist—Civilian six-item version [36], rated on a 5-point Likert scale (0=not at all and 4=extremely; α=.76; range 0-24, with higher scores indicating higher PTSD symptoms). Perceived level of well-being was assessed with the World Health Organization 5-Item Well-Being Index [37], rated on a 6-point Likert scale (0=at no time and 5=all of the time; α=.72; range 0‐25, with higher scores indicating better well-being). Functional impairment was measured with the 12-item World Health Organization Disability Assessment Schedule [38], rated on a 5-point scale (1=no difficulty and 5=extreme difficulty; α=.87; range 0‐60, with higher scores indicating greater functional impairment). Social functioning was assessed with 9 items from a modified version of the Social Adjustment Scale—Self Report [39,40], asking for frequency and quality of interactions with family and friends and spending time on leisure-time activities. The items are rated on a 5-point Likert scale (1=not at all and 5=all the time; α=.65; range 9‐45, with higher scores indicating greater social adjustment). Self-identified problems were measured with the Psychological Outcomes Profiles [41], asking participants to generate 2 salient problems and rate their impact overall on a 5-point scale (1=not at all and 5=severely affected; Spearman-Brown coefficient=0.66; range 0‐10, with higher scores indicating greater reported problems). Eight items adapted from the Harvard Trauma Questionnaire [42] were used to assess exposure to potentially traumatic events, rated as 1=yes and 0=no. Seven items adapted from the modified version of the Post-Migration Living Difficulties Checklist [43-45] were used to measure experiences of postdisplacement stressors in Indonesia, with items rated on a 5-point scale (0=a small problem and 4=a very serious problem).

All translated measures underwent an extensive review process to ensure their comprehension and appropriateness for use with Afghan refugees in Indonesia. In line with recent recommendations [46], a group of Afghan refugee community members (team leaders, trained assessors, and facilitators involved in the study) reviewed each scale and collaboratively refined the wording of specific items to ensure that they were appropriate for the context of this study.

### Statistical Analysis

Based on previous studies with Self-Help Plus (the group-based version of DWM) [13,15], we hypothesized a 0.40 between-groups effect size on our primary outcome (K10) and calculated that a minimum of 73 participants per condition would be required to achieve 80% power for α=.05. Given that we were also interested in investigating mechanisms within the DWM condition, we required a larger sample size in this group (n=146). Estimating at 350% attrition rate due to the mobility of the refugee population in Indonesia, a final sample size of 303 participants (DWM: n=202 and RAC: n=101) was planned.

For our primary analysis, we investigated the impact of DWM on the total K10 score at each time point in the intention-to-treat sample. We used a linear mixed models approach to estimate the between-groups effect of DWM versus RAC at mid-treatment, posttreatment, and follow-up. This model had time as a fixed effect, covaried for baseline K10 score, and included participant as a random effect. We constrained the fixed effect of the intervention to be 0 and created dummy variables for time at mid-treatment, posttreatment, and follow-up (with pretreatment being the reference group). We then included dummy-coded time×intervention interaction terms in the model for each time point. This yielded estimates of the average between-group differences in K10 score for DWM versus RAC at each time point, controlling for baseline scores on K10. Robust inference for fixed effects was obtained using cluster-robust (sandwich) SEs with the small-sample correction, implemented via the *clubSandwich* package in R (R Foundation for Statistical Computing), with clustering at the participant level. Statistical tests and *P* values were based on Satterthwaite-type degrees of freedom derived from the CR2 variance estimator. We next tested whether the same pattern of results was found when adjusting for covariates (age, gender, count of traumatic experiences, and time spent in Indonesia), in the per-protocol population, among follow-up completers, and for secondary outcomes using the same approach described earlier. We calculated effect sizes (Cohen *d*) by dividing the between-groups effect by the pooled baseline SD, adjusting for baseline scores on the outcome variable. We calculated Reliable Change Index scores for the K10 following the procedure outlined by Jacobson and Traux [47] to examine whether changes from baseline to mid-treatment, posttreatment, and 1-month follow-up assessments were reliable and clinically meaningful in addition to being statistically significant. All statistical analyses were conducted in R Studio (version 4.4.3; Posit Software, PBC).

## Results

**Figure 1.**
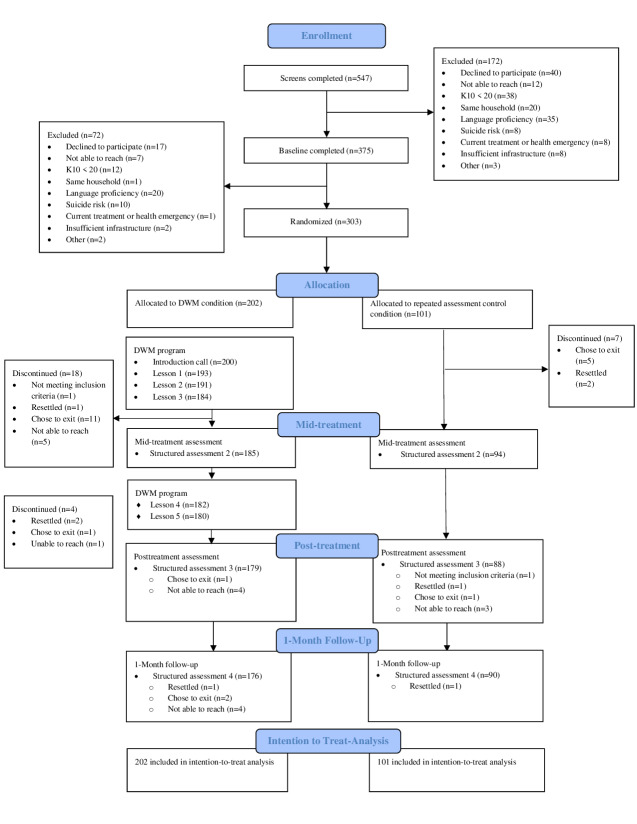
CONSORT flow diagram. CONSORT: Consolidated Standards of Reporting Trials; DWM: Doing What Matters in Times of Stress; K10: Kessler Psychological Distress Scale.

Participant recruitment was conducted from June 2024 to March 2025. One-month follow-up assessments were completed in June 2025. A total of 547 potentially eligible participants completed the screening assessment, of whom 375 proceeded to the baseline assessment. Of these 375 participants, 72 participants were excluded because of limited Farsi proficiency (n=20); self-withdrawal from the study (n=17); decreased K10 scores since the screening call, which rendered them ineligible for participation (n=12); suicide risk (n=10); and other reasons (eg, currently receiving psychological treatment, lack of private space in which to do calls, and no access to smartphone or internet; n=13). Of the 375 people who completed baseline assessments, 303 were randomized to the DWM or RAC condition. The flow of the participants is provided in the CONSORT (Consolidated Standards of Reporting Trials) diagram (Figure 1).

The retention rate from baseline to follow-up assessment was 88%. There were no significant differences in the distributions of participants lost to follow-up between the study conditions at any assessment point (Multimedia Appendix 3). The comparison between the participants who were lost to follow-up and retained on key characteristics is presented in Multimedia Appendix 4. The majority of the participants in the DWM condition completed all 5 lessons (n=180, 89%). No serious adverse event was reported.

Participant characteristics are presented in Table 1. Approximately two-thirds of participants were men (which is consistent with the gender representation of refugees in Indonesia). Most participants were single, originated from Afghanistan, and were living in Bogor, a large city located approximately 60 km from Jakarta. The majority of participants were living in independent housing, were separated from all their immediate family, and were not earning money. On average, participants had been in Indonesia for over 8 years.

**Table 1.** Sociodemographic characteristics of participants in the DWM^a^ and RAC^b^ groups.

	All participants	DWM condition	RAC condition
Age (years), mean (SD)	30.76 (8.54)	31.19 (8.96)	29.89 (7.61)
Gender, n (%)			
Women	81 (27)	60 (30)	21 (21)
Men	222 (73)	142 (70)	80 (79)
Education level, n (%)			
No education or primary education	177 (58)	115 (57)	62 (61)
High school or trade training	100 (33)	68 (34)	32 (32)
Tertiary education	26 (9)	19 (9)	7 (7)
Marital status, n (%)			
Married or in a relationship	108 (36)	73 (36)	35 (35
Not currently in a relationship	195 (64)	129 (64)	66 (65)
Country of origin, n (%)			
Iran	22 (7)	16 (8)	6 (6)
Afghanistan	278 (92)	184 (91)	94 (93)
Pakistan	3 (10)	2 (10)	1 (1)
City of residency, n (%)			
Bogor	188 (62)	122 (60)	66 (65)
Jakarta	69 (23)	48 (24)	21 (21)
Other	46 (15)	32 (16)	14 (14)
Living situation, n (%)			
Independent housing	270 (89)	180 (89)	90 (89)
Refugee shelter	33 (11)	22 (11)	11 (11)
Financial status, n (%)			
Earning money	63 (21)	47 (23)	16 (16)
Not earning money	238 (79)	155 (77)	83 (84)
PTE^c^ exposure, mean (SD)	2.71 (1.82)	2.81 (1.83)	2.52 (1.79)
Time in Indonesia (years), mean (SD)	8.33 (2.37)	8.29 (2.34)	8.39 (2.43)
Family separation, n (%)			
No immediate family in Indonesia	182 (60)	117 (58)	65 (64)
Some immediate family in Indonesia	73 (24)	50 (25)	23 (23)
All immediate family in Indonesia	48 (16)	35 (17)	13 (13)
Previous psychological treatment, n (%)			
No	228 (75)	149 (74)	79 (78)
Yes	75 (25)	53 (26)	22 (22)
Severity of daily stressors, mean (SD)	2.46 (0.70)	2.42 (0.72)	2.53 (0.67)
Psychological distress (K10^d^), mean (SD)	33.14 (6.26)	32.93 (6.20)	33.55 (6.38)
PTSD^e^ symptoms total score (PCL^f^), mean (SD)	13.82 (4.79)	13.65 (4.89)	14.17 (4.59)
Well-being total score (WHO-5^g^), mean (SD)	5.89 (4.00)	6.06 (3.90)	5.55 (4.18)
Disability total score (WHODAS^h^), mean (SD)	29.07 (9.21)	28.83 (8.99)	29.56 (9.66)
Social functioning total score (SAS-SR^i^), mean (SD)	25.40 (5.11)	25.93 (5.17)	24.35 (4.86)

a DWM: Doing What Matters in Times of Stress.

b RAC: repeated assessment control.

c PTE: potentially traumatic event.

d K10: Kessler Psychological Distress Scale.

e PTSD: posttraumatic stress disorder.

f PCL: PTSD Checklist.

g WHO-5: World Health Organization 5-Item Well-Being Index.

h WHODAS: World Health Organization Disability Assessment Schedule.

i SAS-SR: Social Adjustment Scale—Self-Report.

In relation to treatment fidelity and facilitator competency, treatment fidelity was near-perfect (97%), and the facilitators’ competency was consistently rated at least acceptable across 11 domains, with the majority rated as excellent (96% overall).

The results of intent-to-treat linear mixed models investigating differences in DWM and the RAC condition on primary and secondary outcome measures are presented in Table 2. Compared to the RAC, DWM led to significantly greater improvements in psychological distress (primary outcome; posttreatment: *β*=−.563, SE=0.124; *P*<.001; *d*=−0.563; follow-up: *β*=−.447, SE=0.140; *P*=.002; *d*=−0.447), as well as PTSD symptoms (posttreatment: *β*=−.471, SE=0.115; *P*<.001; *d*=−0.471; follow-up: *β*=−.406, SE=0.126; *P*=.002; *d*=−0.406), well-being (posttreatment: *β*=.601, SE=0.144; *P*<.001; *d*=0.601; follow-up: *β*=.324, SE=0.152; *P*=.04; *d*=0.324), functional impairment (posttreatment: *β*=−.216, SE=0.109; *P*=.048; *d*=−0.226; follow-up: *β*=−.226, SE=0.114; *P*=.049; *d*=−0.216), social functioning (posttreatment: *β*=.339, SE=0.119; *P*=.005; *d*=0.339; follow-up: *β*=.255, SE=0.118; *P*=.03; *d*=0.255), and personally identified psychological problems (posttreatment: *β*=−.401, SE=0.137; *P*=.003; *d*=−0.401; follow-up: *β*=−.321, SE=0.152; *P*=.04; *d*=−0.321). Notably, the RAC condition also showed significant decreases in psychological distress, PTSD symptoms, well-being, functional impairment, and social functioning at follow-up. The same results were obtained in the covariate-adjusted model (Table 3).

**Table 2.** Summary statistics of results for primary and secondary outcomes at each time point (intent-to-treat analyses).

	DWM^a^ estimated average value (SE)	RAC^b^ estimated average value (SE)	b (SE)	*P* value	*β* (SE)
K10^c^ score					
Mid-treatment	29.141 (0.447)	32.034 (0.518)	−2.893 (0.689)	<.001	−.462 (0.110)
Posttreatment	27.580 (0.485)	31.100 (0.602)	−3.520 (0.779)	<.001	−.563 (0.124)
Follow-up	26.465 (0.529)	29.261 (0.691)	−2.796 (0.877)	.002	−.447 (0.140)
PCL^d^ score					
Mid-treatment	11.614 (0.315)	13.928 (0.392)	−2.314 (0.510)	<.001	−.483 (0.106)
Posttreatment	11.516 (0.349)	13.772 (0.422)	−2.256 (0.553)	<.001	−.471 (0.115)
Follow-up	10.888 (0.375)	12.834 (0.466)	−1.946 (0.603)	.002	−.406 (0.126)
WHO-5^e^ score					
Mid-treatment	8.352 (0.343)	6.850 (0.379)	1.503 (0.514)	.004	.376 (0.129)
Posttreatment	8.999 (0.383)	6.597 (0.423)	2.402 (0.577)	<.001	.601 (0.144)
Follow-up	9.177 (0.391)	7.884 (0.457)	1.293 (0.608)	.04	.324 (0.152)
WHODAS^f^ score					
Mid-treatment	26.130 (0.597)	28.040 (0.756)	−1.910 (0.973)	.05	−.207 (0.106)
Posttreatment	25.333 (0.592)	27.416 (0.858)	−2.082 (1.050)	.049	−.226 (0.114)
Follow-up	23.790 (0.664)	25.781 (0.737)	−1.991 (1.002)	.048	−.216 (0.109)
SAS-SR^g^ score					
Mid-treatment	30.451 (0.365)	28.481 (0.460)	1.970 (0.597)	.001	.385 (0.117)
Posttreatment	30.634 (0.342)	28.899 (0.493)	1.735 (0.611)	.005	.339 (0.119)
Follow-up	31.160 (0.352)	29.854 (0.483)	1.306 (0.606)	.03	.255 (0.118)
PSYCHLOPS^h^					
Mid-treatment	8.724 (0.127)	9.003 (0.130)	−0.279 (0.182)	.13	−.221 (0.109)
Posttreatment	8.684 (0.118)	9.191 (0.126)	−0.507 (0.173)	.003	−.401 (0.137)
Follow-up	8.565 (0.143)	8.971 (0.128)	−0.406 (0.193)	.04	−.321 (0.152)

a DWM: Doing What Matters in Times of Stress.

b RAC: repeated assessment control.

c K10: Kessler Psychological Distress Scale.

d PCL: PTSD Checklist.

e WHO-5: World Health Organization 5-Item Well-Being Index.

f WHODAS: World Health Organization Disability Assessment Schedule.

g SAS-SR: Social Adjustment Scale—Self-Report.

h PSYCHLOPS: Psychological Outcomes Profiles.

**Table 3.** Summary statistics of results for primary and secondary outcomes at each time point (intent-to-treat analyses for covariate-adjusted model).

	DWM^a^ estimated average value (SE)	RAC^b^ estimated average value (SE)	b (SE)	*P* value	*β* (SE)
K10^c^ score					
Mid-treatment	29.096 (0.440)	32.113 (0.521)	−3.017 (0.691)	<.001	−.482 (0.110)
Posttreatment	27.532 (0.479)	31.176 (0.607)	−3.644 (0.783)	<.001	−.583 (0.125)
Follow-up	26.418 (0.523)	29.340 (0.696)	−2.923 (0.880)	.001	−.467 (0.141)
PCL^d^ score					
Mid-treatment	11.570 (0.309)	14.016 (0.382)	−2.446 (0.501)	<.001	−.511 (0.105)
Posttreatment	11.466 (0.348)	13.856 (0.418)	−2.390 (0.552)	<.001	−.499 (0.115)
Follow-up	10.835 (0.369)	12.920 (0.464)	−2.085 (0.601)	<.001	−.435 (0.125)
WHO-5^e^ score					
Mid-treatment	8.387 (0.340)	6.781 (0.376)	1.607 (0.512)	.002	.402 (0.128)
Posttreatment	9.038 (0.382)	6.534 (0.420)	2.504 (0.575)	<.001	.627 (0.144)
Follow-up	9.223 (0.392)	7.816 (0.460)	1.407 (0.614)	.02	.352 (0.154)
WHODAS^f^ score					
Mid-treatment	26.076 (0.592)	28.142 (0.751)	−2.066 (0.969)	.03	−.224 (0.105)
Posttreatment	25.271 (0.590)	27.507 (0.856)	−2.236 (1.050)	.03	−.243 (0.114)
Follow-up	23.722 (0.660)	25.874 (0.737)	−2.153 (1.003)	.03	−.234 (0.109)
SAS-SR^g^ score					
Mid-treatment	30.507 (0.352)	28.367 (0.454)	2.140 (0.585)	<.001	.418 (0.114)
Posttreatment	30.693 (0.332)	28.790 (0.480)	1.903 (0.598)	.001	.372 (0.117)
Follow-up	31.219 (0.347)	29.742 (0.481)	1.477 (0.602)	.02	.289 (0.118)
PSYCHLOPS^h^					
Mid-treatment	8.717 (0.123)	9.027 (0.130)	−0.310 (0.180)	.09	−.245 (0.142)
Posttreatment	8.675 (0.115)	9.211 (0.123)	−0.536 (0.169)	.001	−.424 (0.134)
Follow-up	8.550 (0.140)	8.993 (0.127)	−0.443 (0.190)	.02	−.350 (0.150)

a DWM: Doing What Matters in Times of Stress.

b RAC: repeated assessment control.

c K10: Kessler Psychological Distress Scale.

d PCL: PTSD Checklist.

e WHO-5: World Health Organization 5-Item Well-Being Index.

f WHODAS: World Health Organization Disability Assessment Schedule.

g SAS-SR: Social Adjustment Scale—Self-Report.

h PSYCHLOPS: Psychological Outcomes Profiles.

Per-protocol results are presented in MultimediaAppendices 5  6. Findings were consistent with the intention-to-treat analyses. Sensitivity analyses with participants who completed the follow-up assessment found a similar pattern, except that the DWM condition no longer showed greater improvements than the RAC condition in functional impairment at posttreatment (*β*=−.323, SE=0.123; *P*=.09) and follow-up (*β*=−.255, SE=0.121; *P*=.06).

At posttreatment and follow-up assessments, the DWM condition had more participants who recovered or showed reliable improvement in their K10 symptoms compared to the RAC condition. Similarly, the proportion of those who deteriorated was lower in the DWM condition than in the RAC condition at each assessment (Table 4).

**Table 4.** Reliable Change Index (RCI) for Kessler Psychological Distress Scale (K10) across assessments^a^.

RCI	Mid-treatment^b^		Posttreatment^c^		One-month follow-up^d^	
	DWM^e^, n (%)	RAC^f^, n (%)	DMW, n (%)	RAC, n (%)	DWM, n (%)	RAC, n (%)
Recovered	24 (13)	7 (7)	36 (20)	4 (5)	45 (26)	9 (10)
Improved	36 (20)	6 (6)	41 (23)	15 (17)	50 (28)	18 (20)
Deteriorated	5 (3)	3 (3)	5 (2)	4(5)	4 (2)	3 (3)
No change	120 (65)	78 (83%)	97 (54)	65 (74)	77 (44)	60 (67)

a Four categories of reliable change were computed: recovered (substantial recovery in symptoms), improved without recovery (reliable improvement but no full recovery), deteriorated (reliable worsening of symptoms), and no change (no reliable or clinically meaningful change in symptoms). Recovery was determined by subtracting 2 SDs from the mean scores of the K10 and comparing these thresholds to participants’ scores at each subsequent assessment point. The remaining categories were indexed based on RCI scores: scores greater than +1.96 indicated deterioration, scores less than −1.96 indicated improvement, and scores falling between −1.96 and +1.96 indicated no change.

b *Χ*2_3_=11.7; *P*=.008.

c *Χ*2_3_=14.8; *P*=.002.

d *Χ*2_3_=15.1; *P*=.002.

e DWM: Doing What Matters in Times of Stress.

f RAC: repeated assessment control.

## Discussion

### Principal Findings

We found that DWM was effective in reducing psychological distress and PTSD symptoms, as well as improving well-being and overall daily functioning, in refugees living in a transit country. To our knowledge, this is the first fully powered randomized controlled trial to investigate the efficacy of DWM with refugees, demonstrating both the efficacy and feasibility of an individually guided self-help approach in improving psychological well-being in a context characterized by significant ongoing stressors, prolonged uncertainty, and limited resources. The clinical significance of these results is reflected in the finding that, compared to the repeated assessment group, participants who received DWM showed significantly greater rates of recovery (45/202, 26% vs 9/101, 10%) and improvement (50/202, 28% vs 18/101, 20%) at the follow-up assessment as measured by the Reliable Change Index. A similar pattern of findings was also observed at posttreatment. This study builds on previous research, demonstrating that DWM’s group-based alternative (Self-Help Plus) improved mental health outcomes in Sudanese refugees in Uganda [15], and among refugees without a psychiatric diagnosis in Western Europe [14], and in Türkiye [13]. It also extends on preliminary results, indicating that DWM reduced PTSD symptoms in Syrian refugees [12].

Our findings provide evidence that DWM may be well-suited for refugees subject to prolonged displacement in LMICs. Importantly, guided self-help interventions require relatively few resources to implement, as participants are afforded an illustrated workbook that is complemented by audio files and facilitator support to aid the learning and practice of the intervention strategies [10]. While this approach has previously been found to be efficacious when delivered in a group-based setting [13-15], in this study, participants were provided with individual-level support via five 30-minute Zoom calls from lay facilitators from the refugee community. There is evidence that individually supported approaches may be more effective than group-based interventions [16]. In addition, this one-to-one support mechanism overcomes key barriers to implementation and engagement for refugees in transit settings such as mental health stigma and reluctance to engage in group-based interventions, especially among survivors of persecution and human rights violations [48]. Individual assistance can also overcome logistical challenges, including geographical dispersal of refugee communities, scheduling difficulties, and challenges with assembling and retaining groups of adequate size. In contrast to group-based approaches, individualized support is conducted at a time that is convenient for both the facilitator and the individual, is readily rescheduled, and can be tailored to address the unique psychological needs and practical circumstances of the individual [17]. While the intervention was delivered online in this study (which can increase privacy, reduce stigma [49], and may be well-suited to refugee populations who regularly use smartphones to remain connected to family and friends in other locations [26,27]), it could also be readily delivered in a face-to-face modality, enhancing its generalizability across settings.

The content of the DWM intervention may also be especially appropriate for refugees living with high levels of insecurity in transit settings. In the context of a myriad of uncontrollable stressors relating to refugee status determination, financial insecurity, lack of access to services, and insecure housing in transit countries [50], concrete problem-solving approaches may be difficult to implement. In contrast, the acceptance and commitment-based strategies implemented in DWM, such as “unhooking” from negative thoughts and behaving in a way that is consistent with one’s values in the face of stress [10,31], may be more actionable and provide refugees with tools to cope in contexts where external stressors are intractable and enduring.

It is notable that, in this study, participants in the control condition also showed significant improvements in most study outcomes, with 30% (27/101) reporting clinically significant improvement or recovery at the 1-month follow-up assessment (compared to 95/202, 50% of the DWM condition). This group received matched assessments (4 in total) to the DWM condition from assessors who were trained in empathic listening and basic help-seeking skills. This supportive contact, combined with the expectancy of receiving the DWM materials after the follow-up assessment, may have contributed to symptom improvement in the control group [33]. This finding highlights the potential importance of connection and emotional support in fostering refugees’ well-being. Further, it is interesting to note that the majority of participants who took part in this study were men. While this represents the broader gender balance of refugees in Indonesia, it is contrary to general trends of lower help-seeking among male refugees [51-53]. One possibility is that the online modality may have facilitated greater accessibility and engagement of the intervention among men; alternatively, the economic benefits arising from the vouchers in this study may have encouraged men to participate. Future research should examine patterns of engagement and the benefits of DWM with subgroups of refugees.

Strengths of this study included excellent treatment adherence (99%) and retention to follow-up (88%), nonspecialist community members acting as study facilitators and providing high-quality intervention delivery, and blind and standardized assessments by trained assessors.

### Limitations

We note a number of limitations. First, the study relied on a relatively short follow-up assessment period (1 month); future research testing the efficacy of DWM should evaluate its efficacy in the longer term. Second, the study lacked an active control condition, and as a result, we cannot exclude the possible role of nonspecific factors such as facilitator support, contributing to the effects of DWM. Third, we note that a number of the assessment measures have not been validated with the cultural groups who participated in the study. Finally, digital access requirements may have introduced selection bias.

Amid global calls to better integrate refugee care into the health systems of host transit countries [3], DWM and other guided self-help approaches may form an effective component of stepped-care approaches that combine evidence-based scalable interventions with specialized psychological treatments that are embedded in broader systems of care [11]. Low-intensity approaches that can be effectively delivered by nonprofessionals may be especially beneficial in transit countries like Indonesia, where the gap in access to formal support services is often bridged by refugee-led organizations [23]. Empowering these groups to support community members through guided self-help interventions like DWM has the potential to reduce the substantial mental health burden of refugees living in transit settings. Alongside advocacy and policy-focused efforts to facilitate resettlement opportunities and address structural barriers to well-being in transit contexts, such community-supported mental health initiatives represent scalable solutions that can be sustainably implemented by refugee communities in the context of limited resources.

### Conclusions

This study provides evidence for DWM as an effective approach to reducing psychological distress that can be feasibly supported by lay individuals from refugee communities. The scaling-up of this and other low-cost guided self-help interventions represents an important pathway to increasing access to mental health care for the millions of refugees worldwide.

## Supplementary material

10.2196/89928 Multimedia Appendix 1 Trial protocol.

10.2196/89928 Multimedia Appendix 2 Abbreviated Enhancing Assessment of Common Therapeutic Factors scale.

10.2196/89928 Multimedia Appendix 3 Distributions of lost to follow-up participants.

10.2196/89928 Multimedia Appendix 4 Key characteristics of lost versus retained participants.

10.2196/89928 Multimedia Appendix 5 Summary results for per-protocol analyses.

10.2196/89928 Multimedia Appendix 6 Summary results for completers-only analyses.

10.2196/89928 Checklist 1 CONSORT-eHEALTH checklist (V 1.6.1).

## Abbreviations

CONSORT: Consolidated Standards of Reporting Trials
CONSORT-eHEALTH: Consolidated Standards of Reporting Trials of Electronic and Mobile Health Applications and Online Telehealth
DWM: Doing What Matters in Times of Stress
K10: Kessler Psychological Distress Scale
LMIC: low- and middle-income countries
PTSD: posttraumatic stress disorder
RAC: repeated assessment control
REDCap: Research Electronic Data Capture
UNHCR: United Nations High Commissioner for Refugees
WHO: World Health Organization
