# Supplementary material for: Efficacy of a World Health Organization–Guided Self-Help Intervention for Reducing Psychological Distress in Afghan Refugees: Randomized Controlled Trial
Source: JMIR Ment Health. 2026 May 20;13:e89928. doi: 10.2196/89928 (PMC13189532; doi:10.2196/89928)
Supplement: Multimedia Appendix 1 [file mental-v13-e89928-s001.docx]

**Clinical Trial Protocol**

**Physiological, Psychological, Psychiatric, Surgical or Health Interventions**

Evaluating the efficacy of and mechanisms underlying a low-intensity psychological intervention for refugees

Version 4, June 2024

Professor Angela Nickerson

**Contents**

[1. General Information 3](#_Toc1367914807)

[2. Safety and Monitoring Contacts 5](#_Toc998800240)

[3. Delegation of Clinical Trial Duties 6](#_Toc896626295)

[4. Trial Objectives and Purpose 7](#_Toc1237430820)

[5. Background Information 8](#_Toc1552141954)

[6. Statement of Compliance 9](#_Toc770023119)

[7. Trial Design 10](#_Toc2079352411)

[8. Sample Size 10](#_Toc2094353466)

[9. Selection and Withdrawal of Subjects 11](#_Toc1957862827)

[9.1 Inclusion Criteria 11](#_Toc896736945)

[9.2 Exclusion Criteria 11](#_Toc1316697282)

[9.3 Recruitment Strategy 11](#_Toc334452095)

[9.4 Screening 11](#_Toc1733140792)

[9.5 Consent 14](#_Toc1559245136)

[9.6 Withdrawal of Consent or Participant 14](#_Toc549235422)

[10. Treatment of Subjects 15](#_Toc1914658310)

[11. Safety and Monitoring 25](#_Toc2031980135)

[12. Non-compliance, Protocol Deviation and Serious Breaches of Good Clinical Practice 29](#_Toc782110157)

[12.1 Protocol Deviation 29](#_Toc1950761457)

[12.2 Serious Breach of Good Clinical Practice 29](#_Toc904214944)

[12.3 Reporting Protocol Deviations 30](#_Toc569627656)

[12.4 Reporting of a Serious Breach 30](#_Toc365945128)

[12.5 Reporting of Serious Breaches by Third Parties 30](#_Toc1103647081)

[13. Review of a Protocol Deviation and a Serious Breach 31](#_Toc507064412)

[14. Statistics 31](#_Toc315254910)

[15. Data Ownership 32](#_Toc23513335)

[16. Handling and Reporting Data 32](#_Toc1216802132)

[17. Monitoring Quality Control and Quality Assurance 32](#_Toc1751152291)

[18. Clinical Trial Research Agreement 33](#_Toc229560797)

[19. Research Governance Site Authorisation 33](#_Toc593301310)

[20. Good Clinical Practice Requirements 33](#_Toc441383889)

[21. Essential Documents for the Conduct of a Clinical Trial 33](#_Toc425596996)

[22. Clinical Trial Delegation and Responsibilities Log 34](#_Toc1167714248)

[23. Safety Monitoring Register Template 38](#_Toc939973317)

[24. References 39](#_Toc1023195328)

[25. Certificate of Insurance 43](#_Toc1251000862)

# **General Information**

| **Protocol Title: Improving Mental Health of Refugees in Indonesia** | | | | | | | |
| --- | --- | --- | --- | --- | --- | --- | --- |
|  | | | | | | | |
| **Protocol identifying number** | **iRECS4937** | | | | | | |
| **Version Number** | **4** | | | **Version date** | **June 2024** | | |
| **Amendment History** | | | | | | | |
| **Version Number** | **1**  **2**  **3** | | | **Version date** | **January 2024**  **April 2024**  **June 2024** | | |
| **Clinical Trial Sponsor** | | | | | | | |
| **Sponsor Name** | **Dr Ted Rohr** | | | | | | |
| **Sponsor Contact** | **UNSW Sponsor’s Delegate** | | | | | | |
| **Telephone** | **0417844054** | | | | | | |
| **Email** | [**Ted.rohr@unsw.edu.au**](mailto:Ted.rohr@unsw.edu.au) | | | | | | |
| **Address** | **UNSW Sydney** | | | | | | |
| **Coordinating Principal Investigator** | | | | | | | |
| **Name** | **Angela Nickerson** | | | | | | |
| **Telephone** | **0415 309 992** | | | | | | |
| **Email** | **a.nickerson@unsw.edu.au** | | | | | | |
| **Type of Appointment with UNSW** | **☒** UNSW Employee  ☐ UNSW Conjoint  ☐ Other (Please describe) | | | | | | |
| **Principal Investigator - 1** | | | | | | | |
| **Name** | **Philippa Specker** | | | | | | |
| **Contact** | **Email** | **p.specker@psy.unsw.edu.au** | | | | **Telephone** | **+612 93480445** |
| **Site** | **UNSW Sydney** | | | | | | |
| **Principal Investigator - 2** | | | | | | | |
| **Name** | **Richard Bryant** | | | | | | |
| **Contact** | **Email** | **r.bryant@unsw.edu.au** | | | | **Telephone** | **+612 93853640** |
| **Site** | **UNSW Sydney** | | | | | | |
| **Principal Investigator - 3** | | | | | | | |
| **Name** | **Randy Nandyatama** | | | | | | |
| **Contact** | **Email** | **randy.wirasta@ugm.ac.id** | | | | **Telephone** | **+62 274 88688** |
| **Site** | **Universitas Gadjah Mada, Indonesia** | | | | | | |
| **Principal Investigator - 4** | | | | | | | |
| **Name** | **Belinda Liddell** | | | | | | |
| **Contact** | **Email** | **b.liddell@unsw.edu.au** | | | | **Telephone** | **+612 93853596** |
| **Site** | **UNSW Sydney** | | | | | | |
| **Principal Investigator - 5** | | | | | | | |
| **Name** | **David Keegan** | | | | | | |
| **Contact** | **Email** | **David.keegan@hostint.org** | | | | **Telephone** | **+61(0)414545855** |
| **Site** | **HOST International, Sydney** | | | | | | |
| **Personnel authorised to sign the protocol and the protocol amendment(s) for the Sponsor** (ICH GCP 6.1.3) | | | | | | | |
| **Name** | **Angela Nickerson** | | | | | | |
| **Telephone** | **0415 309 992** | | | | | | |
| **Email** | **a.nickerson@unsw.edu.au** | | | | | | |
| **Address** | **School of Psychology, UNSW Sydney (Kensington Campus)** | | | | | | |
| **Human Research Ethics Committees** | | | | | | | |
| **Name** | | | **UNSW Human Research Ethics Committee & The National Research and Innovation Agency of Indonesia (BRIN)** | | | | |
| **Status of ethical review** | | | **☐ Approved (iRECS4937)** | | | | |
| **Trial Sites** | | | **Indonesia** | | | | |
| **Name** | | | **The National Research and Innovation Agency of Indonesia (BRIN)** | | | | |
| **Status of ethical review** | | | **☐ Approved (BRIN 25032024000003)** | | | | |
| **Trial Sites** | | | **Indonesia** | | | | |
| **Funding for the Clinical Trial** | | | | | | | |
| **Funding Body Name** | | | **NHMRC** | | | | |
| **Amount of Funding** | | | **$1.2 million** | | | | |
| **Interests that the funding body has in the clinical trial** | | | **None** | | | | |
| **Insurance for Clinical Trial** | | | | | | | |
| **Insurer** | | | **UNSW** | | | | |
| **Type of Insurance** | | |  | | | | |
| **Confirmation of Insurance** | | | **☐Attached**  **~~In progress~~**  **~~To be submitted~~** | | | | |

# **Safety and Monitoring Contacts**

| **Clinical Trials Involving Physiological, Psychological, Psychiatric or Surgical Interventions** | |
| --- | --- |
| **Qualified Physician/Medical Expert** | |
| **Name** | **Richard Bryant** |
| **Telephone** | **+61 2 9385 3641** |
| **Email** | **r.bryant@unsw.edu.au** |
| **Address** | **School of Psychology, UNSW** |
| **Sponsors Independent Physician/Medical Expert** | |
| **Name** | **N/A** |
| **Telephone** |  |
| **Email** |  |
| **Address** |  |
| **Pharmacy, Clinical Laboratory, Radiology, Pathology and other medical and technical departments involved in the trial** | |
| **Name** | **N/A** |
| **Telephone** |  |
| **Email** |  |
| **Address** |  |
| **Independent Safety Monitoring Board or Data Safety Monitoring Board Members** | |
| - Dr. Chien Gooi, UNSW - Dr. Natasha Rawson, UNSW - Dr. Winnie Lau, University of Melbourne | |
| **Trial Management Group** | |
| - Dr Angela Nickerson, UNSW - Dr Philippa Specker, UNSW - Dr Gulsah Kurt, UNSW - Anna Camilleri, UNSW - Dessy Susanty, UNSW - Rizka Argadianti Rachmah, UNSW | |

# **Delegation of Clinical Trial Duties**

Responsibilities for the conduct and oversight for the trial are delegated to you as the Coordinating Principal Investigator. You may delegate trial related responsibilities to the listed Principal Investigator(s) and any trial-related personnel. All trial-related duties delegated by the Coordinating Principal Investigator or Principal Investigator(s) and trial-related personnel must only be delegated to those that are qualified by experience and training. Delegated responsibilities must be retained in the [UNSW Clinical Trial Delegation Log](https://research.unsw.edu.au/document/Clinical%20Trial%20Delegations%20Log.docx). The UNSW Sponsor's Delegate is to be notified of the following:

- Protocol deviation reports outlined in the UNSW Research Misconduct Procedure.
- Any serious breach of Good Clinical Practice, the clinical trial protocol, the clinical trial standard operating procedures, or the human ethics approval that is likely to affect to a significant degree the safety or rights of participants or the reliability and robustness of the data generated in the clinical trial.
- Significant safety issues that are likely to (or have the potential to) affect to a significant degree the safety or rights of participants or the reliability and robustness of the data generated in the clinical trial.
- Urgent safety measures implemented to remove or prevent a significant safety issue.
- Safety reports relating to the continuation, suspension, or discontinuation of the clinical trial for safety reasons.
- Non-compliance with the protocol, SOPs, GCP, and applicable regulatory requirement(s) significantly affects or can potentially affect human subject protection or reliability of trial results significantly.
- Participant complaints or concerns received concerning the conduct of the research.
- Significant modifications to the clinical trial are likely to affect a significant degree the safety or rights of participants or the reliability and robustness of the data generated in the clinical trial.
- Addition of participating trial sites, contractual arrangements at participating sites or modifications to legal agreements.
- The intention to conduct the trial in other countries.

# **Trial Objectives and Purpose**

**Aim 1:** To evaluate the *efficacy* of Doing What Matters in Times of Stress (DWM) – a low-intensity mental health intervention – for refugees displaced in Indonesia.

- The hypothesis to be tested is that DWM (DWM workbook + five weekly facilitator sessions) will decrease psychological distress compared to a waitlist control group.

**Aim 2:** To evaluate the *mechanisms* underlying Doing What Matters in Times of Stress (DWM) – a low-intensity mental health intervention – for refugees displaced in Indonesia.

- The hypothesis to be tested is that reductions in psychological distress during and after the provision of DWM will be mediated by improvements in key psychosocial processes (psychological flexibility, emotion regulation, intolerance of uncertainty, and social functioning).

**Primary endpoint:**

This study will implement a waitlist control Randomised Controlled Trial design, where participants will be randomly assigned to active treatment (DWM) or a waitlist control condition (WL) on a 2:1 ratio.

The main study parameter will be psychological distress, measured using the Kessler Psychological Distress Scale – 10-item version (K10; Kessler et al., 2002). A description of this measure can be found under “Measurement Instruments”. This measure is administered at pre-treatment (including as a screening measure), mid-treatment, immediately post-treatment, and 1 month after the completion of treatment.

**Secondary endpoints:**

The measurement instruments are described under ‘Measurement Instruments’.

1. Severity of posttraumatic stress symptoms (PCL-5, 6-item version)
2. Presence and severity of suicidal ideation (SIDAS)
3. Self-identified problems (PSYCHLOPS)
4. Wellbeing (WHO-5)
5. Psychological Flexibility (adapted CompACT & AAQ-II)
6. Repetitive Negative Thinking (PTQ-Short Form)
7. Intolerance of Uncertainty (abbreviated IOUS)
8. Social satisfaction and functioning (abbreviated WHOQoL-BREF items and SAS-SR-modified)
9. Physical health (abbreviated WHOQoL-BREF)
10. Post-Migration Living Difficulties (adapted PMLD Checklist)
11. Exposure to adverse life events (adapted HTQ)
12. Use of emotion regulation strategies (adapted RESS-EMA)
13. Momentary emotions
14. Functional impairment (WHODAS-12)

**Other study parameters/endpoints (if applicable):**

The measurement instruments are described under ‘Measurement Instruments’.

1. Demographic data
2. Treatment fidelity (checklists)
3. Other indicators on intervention delivery: dose, perceived benefit, quality
4. Treatment contamination check

# **Background Information**

Many studies have shown that refugees suffer elevated rates of mental health problems, including depression, anxiety, and posttraumatic stress disorder (Blackmore, 2020). Providing effective mental health interventions for refugees is thus a critical priority for global public health. However, meeting this priority is challenging, as 99% of the world's refugees reside in low- and middle-income countries (LMICs) where access to psychological treatment is extremely limited (UNHCR, 2022). In an attempt to meet this challenge, the World Health Organisation (WHO) recently developed a set of low-intensity scalable mental health interventions, including Doing What Matters in Times of Stress (DWM) (Bryant, 2023). These interventions are ideally suited to LMICs as they teach psychological skills via lay refugee facilitators and self-help methods (thus requiring few resources).

The DWM intervention was selected for this current project for two reasons:

First, due to practical and logistical considerations. Barriers to the delivery and uptake of mental health interventions for refugees in Indonesia include a lack of financial resources to pay for lengthy treatment programs, limited capacity of mental health specialists to deliver specialized services, language barriers between the refugee and host communities and limited ability of refugees to travel to and from physical appointments. To address these barriers, DWM is: inexpensive, remotely delivered, of short duration, and is simple to train to local providers so that it can be made available rapidly to large numbers of people. This is highly relevant to the study context (refugees displaced in Indonesia), where demand for mental health services exceeds the availability.

Second, due to clinical considerations. DWM teaches core cognitive and behavioural techniques that our previous longitudinal research with refugees in Indonesia found to be relevant to promoting mental health in this context (Nickerson et al., 2022; 2023; HC190494). Moreover, we found high rates of *multiple* psychological problems. Contrary to traditional mental health programs, which usually focus on single psychiatric disorders (such as posttraumatic stress disorder; PTSD), DWM is transdiagnostic (i.e., not disorder-specific) to target common mental health symptoms and general psychological distress.

The WHO has developed the DWM intervention (which is part of a suite of low-intensity, short, less expensive and trans-diagnostic programs) to reduce psychological distress and improve psychosocial functioning. It is based on the WHO treatment guidelines for conditions related to stress (WHO, 2013). DWM is a remotely delivered intervention. Participants receive an illustrated workbook (comprising 5 ‘lessons’) and audio files, as well as 5 weekly calls from a trained non-specialised facilitator. The DWM lessons comprise evidence-based techniques from Acceptance and Commitment Therapy: (a) ‘Grounding’ (mindfulness skills), (b) ‘Unhooking’ (thought defusion techniques), (c) Acting on Your Values (values-guided behavioural activation and problem-solving), (d) Being Kind (consolidation of thought defusion techniques for unkind thoughts), (e) Making Room (consolidation of mindfulness techniques for emotional pain; self-care/self-compassion). An earlier derivative of DWM (Self-Help Plus – the face-to-face group version of DWM) was found to be effective for South Sudanese refugees in Uganda (Tol et al., 2020). Specifically, this trial found that the intervention resulted in significant reductions (at post-intervention and/or 3-month's follow-up) in psychological distress, PTSD, depression, functional impairment, subjective wellbeing and self-identified problems. Further, a secondary analysis found preliminary indications that psychological flexibility may be a mechanism of change driving intervention response (Lakin et al., 2023).

However, two gaps in our evidence-base remain. First, this earlier trial was conducted solely with women, in a face-to-face group-based format. To enhance the reach of the program to more people, this intervention has since been adapted for remote and individual delivery, where participants receive a workbook and audio files alongside calls from a trained facilitator. A pilot trial has demonstrated the feasibility of this approach for displaced refugees in Turkey (Acarturk et al., 2022), however a fully-powered efficacy trial of DWM has yet to be conducted. Second, to date, there has been very little interrogation of how DWM works (Lakin et al., 2023; Bryant, 2023). Without clear evidence on the mechanisms responsible for good mental health among refugees, we cannot refine these interventions to more effectively support refugee communities. This project will directly address these gaps by providing a comprehensive investigation of:

(1) the efficacy of the remote, individually delivered format of DWM among male and female refugees displaced in Indonesia, and

(2) the mechanisms of change within the DWM intervention.

The design of the current RCT has been informed by previous studies (Tol et al., 2020; Acarturk et al., 2023) and gold-standards for intervention evaluation. Trials conducted with refugee communities using DWM or similar WHO interventions (including trials with female refugees in Uganda (N=694), terrorism survivors in Pakistan (N=370) and gender-based violence survivors in Kenya (N=520)) have resulted in no direct adverse events. Participants are screened prior to commencing the program so that those requiring more specialized interventions are referred to experienced local service providers based in Indonesia (e.g., UNHCR and Church World Service). The DWM intervention is based on teaching coping skills and is not expected to provoke adverse reactions. These risks are justified by the benefits of the research described above.

**Population:**

Participants will be adult refugees (predominantly from Afghanistan and Iran) currently residing in Indonesia. This sample was selected for two key reasons. First, it represents the majority demographic of refugees displaced in Indonesia. It is estimated that Indonesia hosts approximately 12,000 displaced refugees, with over 50% originating from Afghanistan or Iran. Second, in a previous 2-year longitudinal study of the mental health and experiences of refugees in Indonesia, refugees from these countries of origin evidenced particularly elevated levels of psychological distress (symptoms of PTSD and depression; Nickerson et al., 2023).

# **Statement of Compliance**

The clinical trial will be conducted in compliance with the following guidelines and documentation:

- [ICH Guidelines for Good Clinical Practice (GCP)](https://www.tga.gov.au/publication/note-guidance-good-clinical-practice)
- [National Statement on Ethical Conduct in Human Research](https://nhmrc.gov.au/about-us/publications/national-statement-ethical-conduct-human-research-2007-updated-2018) (National Statement)
- As approved by the Human Research Ethics Committee (HREC), the clinical trial protocol is responsible for monitoring the trial's conduct.
- The responsibilities set out by the UNSW Sponsors Delegate.

The onsite or remote monitoring standard operating procedures as put in place by the clinical trial sponsor.

# **Trial Design**

A single-blind waitlist control RCT design will be used to answer the research questions (see ‘Schematic of Trial Design’ figure below). Participants will be randomized to either an active treatment (DWM) or waitlist (WL) control condition. For those in the active treatment condition, the intervention, DWM, will be delivered over a 5-week period. Participants will receive a workbook and audio files. The DWM workbook comprises 5 'lessons' that participants can work through in a self-paced format. Participants will receive 5-weekly calls by a trained facilitator, who will assist the participant in learning and practicing the key coping strategies of the program. Participants in the WL control condition will gain access to the DWM workbook and audio files following the completion of the WL period.

The trial is a single-blind randomised controlled trial, which means the outcome assessors are blind to treatment allocation. Randomization will be carried out by an independent researcher not involved in intervention delivery, clinical supervision, independent assessment or other aspects of the day-to-day running of the study. Randomization will be performed using computerized software on a 2:1 basis, such that double the number of participants are allocated to the active treatment condition. This randomization ratio was selected to ensure adequate power for study aim 2, to investigate the mechanisms of change *within* the active treatment.

**Schematic of Trial Design**


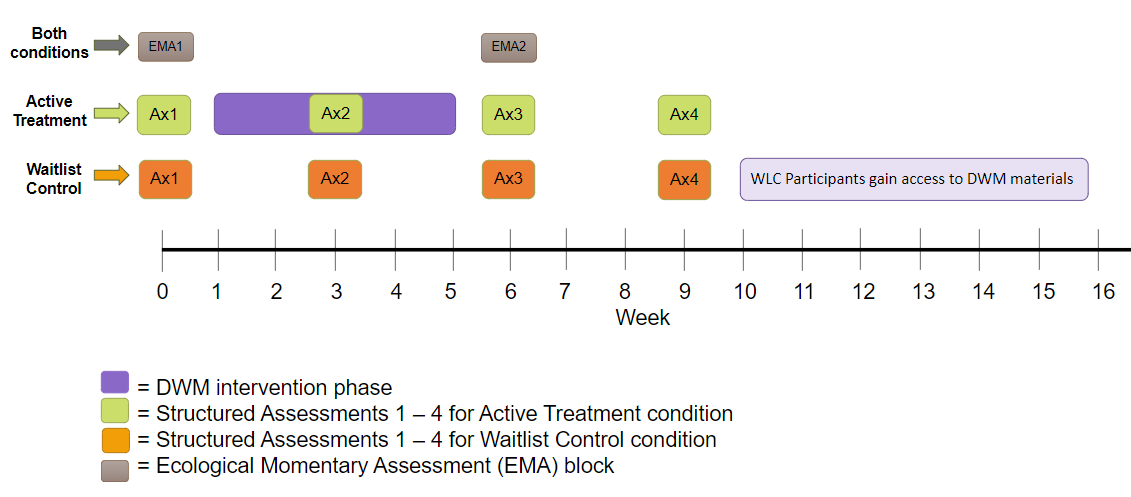


# **Sample Size**

We plan to enrol 303 participants into the trial. As a key focus of this study is to investigate psychological and social mechanisms underlying the impact of DWM on mental health outcomes, we will implement a 2:1 allocation ratio (DWM to WLC) to ensure analyses are adequately powered. Based on previous research, we aim to detect a medium between-groups effect size (0.40) (Tol et al., 2020; Acarturk et al., 2020) on our main outcome measure (Kessler-10). With alpha of 0.05 and power of 80%, we will need to include a minimum of 224 participants across both arms (150 in the DWM arm and 73 in the WLC). We estimate 35% attrition given the mobile nature of the refugee population in Indonesia, and thus we will need to recruit 303 participants into the trial.

# **Selection and Withdrawal of Subjects**

## **Inclusion Criteria**

Inclusion criteria for participants taking part in this study include:

(a) 18+ years,

(b) from a refugee background,

(c) able to speak and read Farsi, and

(d) experiencing elevated levels of psychological distress (K10 > 19.9)

## **Exclusion Criteria**

Exclusion criteria for those who are not eligible to participate in the study include:

(a) participants that do not meet the above inclusion criteria

(b) acute medical conditions,

(c) imminent suicide risk or with expressed acute needs/protection risks (e.g., a woman who reports that she is at acute risk of domestic and/or sexual

violence),

(d) severe mental disorder (psychotic disorders, substance-dependence),

(e) severe cognitive impairment (e.g., severe intellectual disability or dementia), and/or

(f) concurrent psychological treatment (e.g., currently receiving psychological services from a psychologist, counsellor or mental health-focused program)

(g) no access to a smart phone and internet connection.

## **Recruitment Strategy**

The research team will periodically post a study advertisement on our social media accounts (Pathways to Refugee Wellbeing WhatsApp and Instagram accounts). This method of recruitment has previously been used by the research team to recruit 1,235 refugees living in Indonesia for a longitudinal online study (HC190494). Additionally, the study advertisement will be sent (via a WhatsApp message) to refugee participants from our previous research project in Indonesia (HC190494) who gave permission to be contacted about future research studies.

The recruitment ad will direct potential participants to a 5-minute online screener form where interested participants can read further information on the program and decide whether they wish to provide consent to complete the screener form.

## **Screening**

Screening will be conducted in two stages (see Figure below titled, “Schematic of Screening Process and Trial Stages”). First, a brief online screening form will be used to assess inclusion criteria. Second, those who meet the inclusion criteria will then be screened over-the-phone by a trained assessor to verify their responses and additionally assess exclusion criteria.

(a) Online Screener

Potential participants who follow the link in the study advertisement will be directed to a brief online screener form on Qualtrics. The first section of this form is the Participant Information Statement and Consent Form that outlines the two-stage screening process. Potential participants can then indicate if they consent to proceed to the screener. Following informed consent, potential participants will be directed to complete brief questions to verify demographic eligibility (i.e., to verify that they are an adult, Farsi-speaking refugee residing in the Greater Jakarta region of Indonesia). The presence of at least moderate levels of psychological distress will be screened using the Kessler Psychological Distress Scale (K-10; Kessler et al., 2002), where a score above 19.9 will be required to be eligible. (This scale is outlined in *10.1 Trial Intervention*.) Ineligible participants (i.e., those who provided responses that indicate that they do not meet the inclusion criteria) will be advised of their ineligibility immediately within the Qualtrics screener survey or, in any cases where their eligibility needs to be discussed or verified by the team, via email/WhatsApp shortly thereafter. For example, if participants are not eligible for the trial because they are too young or are not from the target language group, they will be provided with feedback explaining why they are not eligible for the study and, where relevant, alternate referral information may be provided. Similarly, if participants are not selected for the trial because they score below the cut-off scores for the K-10, they will be provided feedback on their test outcome and it will be explained why they are not eligible for the study. These feedback scripts have been adapted from the manuals provided by the World Health Orgainsation (see pp. 88-89 of the PM+ intervention manual; WHO, 2016).

(b) Phone Screener

Participants who meet inclusion criteria according to the online screener will then be contacted by a trained assessor for a follow-up phone screener call to verify their eligibility (and assess exclusion criteria). Data collected during the screening call will be recorded by the assessor using Qualtrics.

Individuals who meet the exclusion criteria as described in *9.2 Exclusion Criteria* will be excluded and referred externally for appropriate treatment and support. This refers to individuals with imminent suicide risk or with expressed acute needs/protection risks (for example, a woman who expresses that she is at acute risk of domestic violence or sexual assault). Suicidal ideation will be explored through the ‘assessment of thoughts of suicide’ tool developed by the World Health Organisation (WHO, 2016, pp86). In addition, we will also exclude individuals with severe mental disorder (psychotic disorders, substance-dependence) or severe cognitive impairment (e.g., severe intellectual disability or dementia). Mental, neurological or substance use disorders will be screened with the ‘impairments possibly due to severe mental, neurological or substance use disorders’ tool of the Problem Management Plus (PM+) intervention manual (WHO, 2016, pp87). Individuals meeting the exclusion criteria will be provided with referral information for relevant specialist support hotline and crisis services (UNHCR or Church World Service). If a participant meets any exclusion criteria, they will be immediately notified by the assessor using feedback scripts that have been adapted from the manuals provided by the World Health Orgainsation (see pp. 88-89 of the PM+ intervention manual; WHO, 2016).

Participants that do not endorse any exclusion criteria will be informed of their eligibility for the trial and sent an email/WhatsApp message with a Qualtrics link to the Participant Information Statement and Consent form for the intervention trial. Participants who provide informed consent will then be contacted by the research team to schedule their first assessment.

**Schematic of Screening Process and Trial Stages**

**
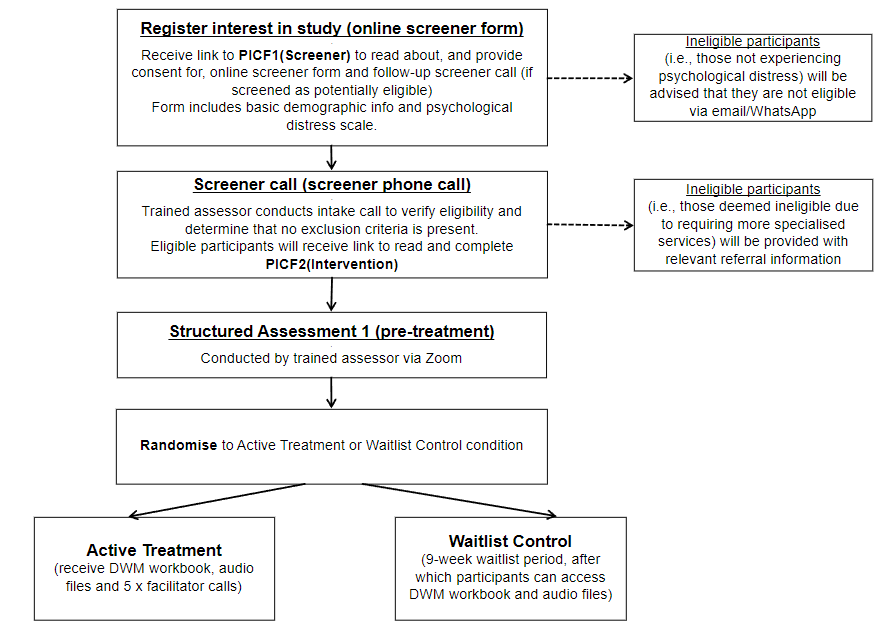
**

## **Consent**

Consent will be obtained in a two stage-process: (1) informed consent for screening, and (2) informed consent for the DWM intervention.

First, consent for the screening process (i.e., online form and, if eligible, follow-up screener call) will be obtained at the beginning of the online screener form. Participants will be directed to the online screener by following the link in the Study Advertisement/Recruitment Ad. The beginning of the online screener will be a brief overview of the research project, followed by the PICF1(Screener) – Easy Read. Participants will be asked to read the consent form. They will have sufficient time to consider their participation as this is an online form, completed without the presence of researchers and in the participant’s own time. Participants will be advised to contact the research team if they have questions. Once they are comfortable providing their consent to participate, they will be asked to provide consent by clicking the relevant checkbox in the online form, after which, they will complete the online screener. If a participant declines to consent, they will not be contacted about the research study. In the event that potentially interested participants contact the team directly to ask questions and/or express their interest in participating, the researcher, once answering their questions, will either re-direct them to the online screener or, if time permitting, administer the screener questions verbally. In the event of verbal screening, real or perceived coercion will be avoided because the research member will make it explicit that participation in the screener is voluntary, and that declining to participate will not affect the participant’s relationship with UNSW. Participants will be provided with the opportunity to ask further questions before consenting. If participants decline consent to screening, they will not be contacted.

Second, participants who are assessed as eligible, following the online and/or phone screening process, will be sent an email/WhatsApp message with a link to the PICF2(Intervention) – Easy Read. Participants will be asked to read the consent form. They will have sufficient time to consider their participation as this is an online consent form, completed without the presence of researchers and in the participant’s own time. Participants will be advised to contact the research team if they have questions. If they wish to consent to participation in the trial, they can provide consent by clicking the relevant checkbox in the online form. Participants who provide consent will then be contacted by the research team to book their first Structured Assessment to commence participation.

This written consent process is appropriate for the data collection method and participant group because potential participants will have ample time to consider their participation in this study, all information about the study will be presented in Farsi (the participants’ own language), and if participants have any queries, they are able to contact the research team.

## **Withdrawal of Consent or Participant**

**Withdrawal of Individual Subjects**

Subjects can leave the study at any time for any reason if they wish to do so without any consequences. The investigator can decide to withdraw a subject from the study for urgent medical reasons, protection needs or resettlement changes.

- Specific criteria for withdrawal (if applicable): Not applicable for this study.
- Replacement of individual subjects after withdrawal: No new subjects will be included for each withdrawn subject.
- Follow-up of subjects withdrawn from treatment: If a subject decides to withdraw from the study, the investigator will ask for the reason. It will be enquired whether the subject wishes to withdraw from the entire study or from a specific time point only, to determine whether the subject wishes to be re-contacted at a later time.

# **Treatment** **of Subjects**

The DWM Intervention Program

DWM is a brief, remotely delivered, self-help intervention (see Doing what matters in times of stress: an illustrated guide. Geneva: World Health Organization; 2020 (<https://www.who.int/publications-detail-redirect/9789240003927> ). It is an illustrated guide of an existing intervention called Self-Help Plus (SH+) (Epping-Jordan et al., 2016). The DWM manual was released by the WHO in 2020 to support the remote delivery of SH+, considering the COVID-19 pandemic and a growing need for online/remotely delivered interventions. DWM is:

- based on acceptance and commitment therapy and adapted for many settings;
- intended as an intervention for managing stress and coping with adversity;
- relevant for anyone above the age of 18;
- designed to be completed over a short period of time;
- practical so that participants can practise skills regularly on their own; and
- illustrated, with accompanying audio recordings to support regular practice.

DWM can be delivered as either a guided or unguided intervention. For the current project, we are testing the guided version of DWM. This means that participants in the active treatment condition will receive a copy of the DWM illustrated workbook that contains five ‘lessons’. This workbook will be accompanied by audio files that provide guided scripts for practicing the newly learnt techniques. Finally, participants will also receive five weekly calls from a trained facilitator, who will help participants to learn and practice the therapeutic techniques. This delivery format is empirically supported by meta-analyses on the therapeutic effects of bibliotherapy, prerecorded self-help interventions, and guided self-help interventions (Cuijper, Munoz, Clarke & Lewinson, 2009; Cuijpers, Donker, van Straten & Andersson, 2010). Participants in the waitlist control condition will gain access to the unguided version of DWM (i.e., workbook and audio files) upon completion of their final assessment at the end of the 9-week waitlist period.

The DWM program is based on Acceptance and Commitment Therapy (ACT; Hayes et al., 2013) techniques that are empirically supported (Hacker, Stone & MacBeth, 2016) and formally recommended by the WHO. The manual involves the following empirically supported elements: psychoeducation, grounding, cognitive defusion, mindfulness exercises, values clarification, and compassion exercises. There is substantial evidence linking ACT-based interventions to reductions in anxiety, depression and stress (Hacker et al., 2016; French, Golijani-Moghaddam & Schroder, 2017).

DWM Skills and Modules

DWM seeks to reduce psychological symptoms by introducing stress management techniques. It comprises five weekly modules, each focused on a specific skill (see Table 1). The specific skill is first learnt via the DWM illustrated workbook and accompanying audio file(s), and then practiced and reinforced by the weekly session with a trained facilitator.

*Table 1. Overview of DWM Modules*

| **Lesson (Overall Theme)** | **Main Goal(s)** | **Core technique(s)** |
| --- | --- | --- |
| 1 (Grounding) | - Practice present-centered awareness during an everyday activity - Practice grounding, to respond effectively to stress | - Psychoeducation - Grounding/Contact with the present moment |
| 2 (Unhooking) | - Practice noticing and naming difficult thoughts and feelings - Refocus on the present | - Grounding/Contact with the present moment - Acceptance - Defusion |
| 3 (Values) | - Identify personal values - Learn to engage in values-guided actions | - Values - Committed Action |
| 4 (Being Kind) | - Enhance kindness to yourself and others | - Compassion towards self and others |
| 5 (Making Room) | - Learn how to accept/tolerate difficult thoughts and feelings via compassion | - Acceptance - Defusion |

The Control Condition

Participants in the Waitlist Control condition will participate in repeated assessments (mid-treatment, post-treatment and follow-up assessments) over a 9-week waitlist period. Following completion of the final assessment, participants will be receive a link to the DWM workbook and audio files.

Use of Co-Intervention (if applicable): Participants are allowed to use any existing prescribed medication or routine care related to any co-morbid health conditions.

- 1. **Trial Intervention**

This trial will be carried out in the Greater Jakarta area of Indonesia. The study will be advertised via the research team’s existing social media channels and participant database. Interested participants will be able to complete an online screener form, which outlines the screening and consent process. Participants who consent to the screener will complete brief questions designed to assess inclusion criteria. Eligible participants will be adult refugees from Farsi-speaking backgrounds who reside in the Greater Jakarta area and score above 19.9 on the K-10. Individuals screened by the online form as potentially eligible will then be contacted by a trained research team member to assess exclusion criteria. Individuals with acute needs, such as imminent suicide or protection risks, will be excluded and referred for appropriate treatment and support. In addition, we will also exclude individuals with severe mental disorders (psychotic disorders, substance-dependence) or severe cognitive impairment (e.g., severe intellectual disability or dementia). We will screen for these exclusion criteria using the tools developed by the World Health Organisation and outlined in their Problem Management Plus (PM+) intervention manual (WHO, 2016, pp. 86-87). Individuals meeting the exclusion criteria will be referred to UNHCR (Jakarta Office) or Church World Service, or to local social service provision, depending on their needs. If the participant agrees, the assessment results will be provided to the referring organisation with permission of the participant and the research team.

Participants deemed eligible based on the online screener and phone screener process will be invited to participate in the trial and sent a link to review the trial’s Participant Information Statement and Consent Form. Following informed consent, participants will be contacted to book their first assessment (Structured Assessment 1). Structured Assessments will be conducted by trained research assistants called ‘Assessors’ (see ‘Trial Personnel’ below) and will involve the administration of the scales in a structured interview format to assess mental health and psychosocial functioning (see ‘Measurement Instruments’ below). If, at the time of the baseline assessment (Structured Assessment 1), the participant’s symptoms of psychological distress have spontaneously improved such that they no longer score above the cut-off, they will be provided feedback on their test outcomes and it will be explained why they are no longer eligible for the study.

After the baseline assessment, participants will be randomized on a 2:1 (active treatment : waitlist control) basis by an independent research assistant, not involved in the assessments, using computerized software. Participants will be randomised to either the Active Treatment condition (*n*=214) or the Waitlist Control condition (*n*=107) to form a single-blind waitlist control RCT design. Participants in the Active Treatment condition will receive the 5-week DWM intervention from Weeks 1-5. Regardless of condition, all participants will complete Structured Assessments every 3 weeks.Upon completion of the final Structured Assessment (1-month follow-up, conducted 9-weeks following baseline assessment), participants in the Waitlist Control condition will gain access to the DWM workbook and audio files.

The instruments administered in Structured Assessments 2-4 closely resemble those used in Structured Assessment 1, with minor changes (as some scales do not need to be re-administered). This is outlined in the ‘Measurement Instruments’ section below. In case participants do not attend a scheduled assessment, the research team will attempt to contact them via follow-up text messages and/or phone calls for a maximum of eight times for scheduling a new appointment.

Participants in both conditions will also complete two 1-week ecological momentary assessment (EMA) blocks, initiated the day following pre-treatment assessment and again following post-treatment assessment (in week 6). At the end of Structured Assessment 1 (pre-treatment), assessors will explain the EMA process and guide participants in downloading and setting-up the Avicenna application on their smart phone. The participant and assessor will then complete a practice survey together. The next day, the participant’s 7-day block will commence. At five random times between 10am and 10pm each day, participants will receive notifications to complete a 1-2 minute survey on their phone. This procedure will be repeated in week 6, following the completion of Structured Assessment 3 (post-treatment). During an EMA block, if participants miss 2 or more of the 5 daily surveys, they will be contacted (either via text message or call) to check whether the participant is experiencing any issues (such as technological difficulties) that the team can assist with to support full participation.

**Trial Personnel:**

Facilitators will be responsible for conducting weekly DWM sessions with participants, via Zoom or phone calls. Facilitators are bilingual (English/Farsi) speakers with prior experience working with people from refugee backgrounds in Indonesia (e.g., previously working as teachers at refugee learning centers, staff in refugee-led originisations and/or interpreters for refugee-focused NGOs in Indonesia). Facilitators will receive 28 hours of in-person training by the research team. The in-person training covers: the DWM manual and techniques, general counselling/facilitation techniques, and responding to participant distress. Ongoing monitoring of facilitators’ capacity to practice will be conducted through regular supervision by a member of the research team who is a Clinical Psychologist. This oversight will help to ensure that any potential concerns about the capacity of the facilitators to carry out their roles is identified and addressed. Protocol adherence will be ensured by regular supervision of the facilitators and treatment fidelity checks using a checklist.

Assessors will be responsible for conducting the Structured Assessments with participants, via Zoom or phone calls. Assessors are bilingual (English/Farsi) speakers with prior experience working with people from refugee backgrounds in Indonesian (e.g., previously working as teachers at refugee learning centers, staff in refugee-led organisations and/or interpreters for refugee-focused NGOs in Indonesia). Assessors will receive 28 hours of in-person training by the research team. The in-person training covers: administering the instruments, general interview techniques, and responding to participant distress. Ongoing monitoring of assessors’ capacity to practice will be conducted through regular supervision by a member of the research team who is a Clinical Psychologist. This oversight will help to ensure that any potential concerns about the capacity of the assessors to carry out their roles is identified and addressed. Protocol adherence will be ensured by regular assessment fidelity checks using a checklist.

Supervisors are responsible for supervising the facilitators and assessors in the day-to-day running of the trial. Supervisors will hold at least Masters-level qualifications in Clinical Psychology.

**Trial Treatment:**

The active treatment is the DWM intervention (see Section 10. Treatment of Subjects for a summary of this intervention and its modules). Participants in the Active Treatment condition will receive the guided version of DWM. This involves receiving the DWM workbook, audio files, and five weekly facilitator calls. Participants in the Waitlist Control condition will gain access to the DWM workbook and audio files upon the completion of their study participation (i.e., following the completion of the final assessment in week 9).

**Measurement Instruments:**

The instruments used to assess and monitor mental health and psychosocial functioning are outlined in the below table.

|  | **When measure is administered** | | | | |
| --- | --- | --- | --- | --- | --- |
| **Construct & Measure** | **Screener** | **Structured Assessment 1** | **Structured Assessments 2-4** | **Ecological Momentary Assessment** | **DWM sessions** |
| Demographics | X | X |  |  |  |
| Psychological Distress (K10)* | X | X | X |  |  |
| Posttraumatic Stress Reactions (PCL-6) |  | X | X |  |  |
| Suicidal Ideation (SIDAS) |  | X | X |  |  |
| Self-Identified Problems (PSYCHLOPS) |  | X | X |  |  |
| Wellbeing (WHO-5) |  | X | X |  |  |
| Psychological Flexibility (adapted CompACT & AAQ-II) |  | X | X |  |  |
| Repetitive Negative Thinking (PTQ- Short form) |  | X | X |  |  |
| Intolerance of Uncertainty (abbreviated IoUS) |  | X | X |  |  |
| Social Satisfaction and Functioning (abbreviated WHOQoL-BREF items and SAS-SR-modified) |  | X | X |  |  |
| Physical Health (abbreviated WHOQoL-BREF) |  | X | X |  |  |
| Post-Migration Living Difficulties (adapted PMLD Checklist) |  | X | X |  |  |
| Perceived benefit (newly adapted items) |  |  |  | X |  |
| Adverse Life Events (adapted HTQ) |  | X |  |  |  |
| Digital Markers** |  | X | X |  |  |
| Momentary Emotions and Stressors |  |  |  | X |  |
| Emotion Regulation Strategies (RESS-EMA) |  |  |  | X |  |
| Treatment fidelity (checklists) |  |  |  |  | X |
| Treatment contamination check |  | X | X | X |  |

*Psychological Distress (K10) is the primary outcome measure

**Digital Markers are measured in Structured Assessments 1 and 3 only.

*Screener and Primary Outcome Measure*

*Psychological Distress*

Psychological distress will be measured through the Kessler-10 Psychological Distress Scale (Kessler et al., 2002). Ten items related to distress are rated on a five-point Likert scale. The sum of the ten items gives a total score ranging from 10 to 50. In a study among Kurdish and Afghan (former) refugees and asylum seekers in New Zealand and Australia, the following cut-off scores were used: 10-15.9 (*low risk of psychological distress*), 16-21.9 (*moderate levels of distress consistent with a diagnosis of moderate depression and/or anxiety disorder*), 22-29.9 (*high level of distress*) and 30 or more (*possibility of very high or severe levels of distress*) (Sulaiman-Hill & Thompson, 2010). In the current study, we will use a score of >19.9 as an indication of moderate to high levels of psychological distress. The K10 was found to be an accurate screener for common mental disorders cross-culturally (Patel et al., 2008; Fassaert et al., 2009, Sulaiman-Hill & Thompson, 2010, Tol et al., 2020). Furthermore, the K10 strongly correlated with other validated measures for screening for common mental disorders in primary care patients in India (*r*=.68 with GHQ-12 and *r*=.84 with SRQ, respectively) (Patel et al., 2008).

*Secondary Outcome Measures*

*PTSD symptoms*

Posttraumatic stress disorder (PTSD) symptoms during the past week according to a DSM-5 PTSD diagnosis will be measured using the PTSD Checklist for DSM-5, 6-item version (Weathers et al., 2013). Items are rated on a 0-4 scale and are summed to calculate a total severity score, with higher scores indicating worse symptomatology. This measure has previously been used with refugees (Tol et al., 2020).

*Suicidal Ideation*

The Suicidal Ideation Attributes Scale (SIDAS; Van Spijker et al., 2014) will be used to measure the presence and inference of suicidal thoughts over the past week. The scale has 5 items, each rated on a 0-10 scale. Scores are summed to calculate a total severity score, with high scores indicating worse symptomatology. However, if a participant scores zero on the first item, then items 2-4 are not administered.

*Self-identified Problems*

The Psychological Outcomes Profiles (PSYCHLOPS) scale is a patient-generated outcome measure as an indicator of change after therapy (Ashworth et al., 2004). PSYCHLOPS consists of four questions. It contains three domains: problems (2 questions), function (1 question), and wellbeing (1 question). This trial will use the ‘problems’ questions only, as functioning and wellbeing will be measured by other instruments in the assessment battery. Participants are asked to give free text responses to the problem domains. Responses are scored on an ordinal six-point scale. PSYCHLOPS has been validated in primary care populations across several countries (Czachowski, Seed, Schofield, & Ashworth, 2011; Héðinsson, Kristjánsdóttir, Ólason, & Sigurðsson, 2013).

*Wellbeing*

Wellbeing and quality of life over the past week will be measured using the World Health Organisations Wellbeing Index (WHO-5; Topp et al., 2015). The scale comprises 5 items, scored on a 0-5 scale, where higher scores indicate greater wellbeing. Items are summed to create a total score. The measure has previously been used in with refugees (Tol et al., 2020).

*Functional Impairment*

Data on health and functional disability will be collected using the 12-item WHO Disability Assessment Schedule 2.0 (WHODAS 2.0) (WHO, 2010b). The WHODAS assess levels of functional impairement resulting from any disease(s), including mental neurological and substance use disorders. It is simple to administer and applicable across cultures and can be used in all adult populations. The WHODAS covers six domains (cognition, mobility, self-care, getting along, life activities, participation). Difficulties are scored on a five-point Likert scale ranging from 0 (none) to 4 (extreme), before summation (range 0-48). Higher scores indicate worse functional impairment. We will use the 12-item interviewer administered version, which has been validated in different cultural contexts (WHO, 2010a).

*Digital Markers*

Data on facial and vocal markers of positive and negative affect and arousal will be collected digitally, using participants responses to two standardised prompts (Schultebraucks et al., 2022; Meaney et al., Under Review). Following the administration of the PSYCHLOPS (standardised outcome measure noted above), where each participant identified the most salient current problem in their life, participants will be asked to describe this problem and their emotional responses for one minute. Following this, participants will then be asked to describe a salient positive experience, event or relationship and their emotional responses for one minute. The one-minute recordings will be extracted from the Structured Assessment zoom recording for analysis of facial (e.g., expressivity), acoustic (e.g., volume, frequency, amount of speech), speech content (e.g., sentiment) and head movement. Digital measurement of facial and vocal markers of negative affect and arousal have been validated to investigate indices relating to negative affect, arousal and emotion regulation (Meaney et al., Under Review). Further, this integrated form of assessment (that makes use of existing assessment recordings) significantly reduces participant burden. This approach is routinely used with clinical populations, including individuals experiencing severe depression (Abbas et al., 2021), schizophrenia (Abbas et al., 2021; 2022), PTSD (Meaney et al., Under Review) and suicide (Galatzer-Levy et al., 2021).

Hypothesised Mechanisms

*Psychological Flexibility*

The Comprehensive assessment of Acceptance and Commitment Therapy processes (CompACT; Golijani-Moghaddam et al., 2023) will be used to measure the presence of psychological flexibly over the past week. An adapted version of the scale, comprising 10 items, will be administered. Items are scored on a 7-point scale, with some reverse scored items. Items are summed to create three subscale scores indexing: openness to experience, valued action and behavioural awareness. Higher scores indicate higher levels of psychological flexibility in each domain.

Acceptance and Action Questionnaire-II (Hayes et al., 2004) will be used to assess psychological inflexibility over the past week. The scale comprises 7 items (on a 7-point Likert scale). Higher scores indicate higher levels of psychological inflexibility characterized by greater experiential avoidance. This scale has been extensively used in the literature, including randomized controlled trials with forcibly displaced people (Acarturk et al. 2022; Lakin et al., 2023). The psychometric properties of the scale were reported excellent (Ong et al., 2024).

*Emotion Regulation*

In the current study, emotion regulation will be assessed using an ecological momentary assessment (EMA) paradigm, which will involve two 1-week EMA blocks (each comprising 5 ultra-brief surveys per day for 7 days). Each survey will comprise 21 items in total: nine items assessing momentary emotional states (e.g., anger, sadness, relaxation; adapted from Cloos, Ceuleman & Kuppens., 2023), eight items assessing momentary emotion regulation strategy use (e.g., rumination, suppression, acceptance; adapted from the Regulation of Emotion Systems Survey-EMA version; Medland et al., 2020), and 4 items assessing momentary stressors (e.g., positive event, negative event, event unpleasantness/pleasantness; adapted from Baik & Newman, 2023). Items are scored on a 0-100 sliding scale. An EMA paradigm, using similar items, have been employed with refugees (Koch et al., 2020).

*Repetitive Negative Thinking*

The short form of the Perseverative Thinking Questionnaire (PTQ) (Ehring et al., 2011) will be used to measure to which extent participants are impacted by negative repetitive thinking over their problems on a 5-point Likert Scale (0 = Never, 4 = Almost Always). The short form (Moeller et al., 2023) consists of 9 items tapping into three subscales: core feature and unproductiveness of repetitive negative thinking, and mental capacity captured by it. Higher scores indicate higher levels of repetitive negative thinking. The scale has previously been used in several studies, including randomized controlled trials with trauma-exposed populations (Heckendorf et al., 2019).

*Intolerance of Uncertainty*

The Intolerance of Uncertainty Scale-short version (Carleton et al., 2007) will be used to measure the presence of intolerance of uncertainty. The current study will use an abbreviated version of the scale, comprising 6 items rated on a 1-5 scale, to reduce the administration burden on the participant. Items are summed to create two subscale scores indexing: prospective anxiety and inhibitory anxiety. Higher scores indicate higher levels of intolerance for uncertainty in each domain. This scale has previously been used with refugees (Nickerson et al., 2019).

*Social Satisfaction and Functioning*

Social satisfaction over the past week will be measured using two items from the World Health Organisation’s Quality of Life Scale (Brief Version) (WHOQoL-BREF; WHO, 1998). Items are scored on a 1-5 scale, where higher scores indicate greater social satisfaction. This scale has previously been used with refugees (Tissue et al., 2022). The modified version of the Social Adjustment Scale-Self Report (SAS-SR) (Cooper et al., 1982; Weissman & Bothwell, 1976) will be used to assess participants’ social functioning with 9 items rated on a 5-point Likert Scale (1 = Not at all, 5 = All the time). Higher scores indicate higher social functioning over the past 7 days. The scale has been widely used with trauma-exposed populations in a range of studies, including RCTs (Scoglio et al., 2020).

Covariates

*Physical Health*

Physical health over the past week will be measured using two items from the World Health Organisation’s Quality of Life Scale (Brief Version) (WHOQoL-BREF; WHO, 1998). Items are scored on a 1-5 scale, where higher scores indicate better physical health. This scale has previously been used with refugees (Tissue et al., 2022).

*Post-Migration Living Difficulties*

Post-migration stressors will be assessed using a version of the Post-Migration Living Difficulties Checklist (PMLDC) (Silove, Sinnerbrink, Field, Manicavasagar, & Steel, 1997; Steel, Silove, Bird, McGorry, & Mohan, 1999) adapted to the Swiss context. This 17-item scale examines the extent to which post-migration challenges had been of concern to the individual over the past 12 months. Items are rated on a five-point scale, ranging from 0 (*not a problem*) to 4 (*a very serious problem*). Items scored at least 3 (*a serious problem*) are considered positive responses, yielding a total count of living difficulties. This scale has consistently been identified as a predictor of mental health among displaced populations (Nickerson, Bryant, Steel, Silove, & Brooks, 2010; Schweitzer et al., 2006; Steel et al., 2006) and has previously been used with Farsi speaking refugees (Nickerson et al., 2015; Schick et al., 2016). In the current research, we will assess post-migration challenges over the past week and the period since the last assessment (at post-intervention assessment and follow-up).

*Exposure to Adverse Life Events*

Exposure to traumatic events will be assessed using an adapted version of the Harvard Trauma Questionnaire (Mollica et al., 1992). The HTQ measures exposure to different types of adverse life events, such as forced isolation, serious injury and war-related combat, using a 16-item checklist. A total count of the types of adverse life events that each individual had experienced and/or witnessed will be derived. In the current research, an adapted version of the scale (that combines similar items to create 8 items overall) will be used to reduce administration burden for the participant.

Other Measures

*Treatment Contamination Check*

Lifetime and current access to mental health services will be measured using a context-adapted item from the Access to Health Care Services questionnaire developed by the STRENGTHS project (Fuhr et al., 2020). Also, participants will be asked to indicate if they gained informal access to the active intervention materials (DWM workbook or audio files) before starting the trial or while undergoing the WL period in the trial. These questions will be in self-report format (rather than interview format), administered at the conclusion of the final EMA survey block, to reduce demand characteristics.

*Perceived Benefit*

Participants’ subjective perception of whether participation in the study led to any direct benefits (i.e., psychological, social or economic benefit) will be assessed using brief self-report questions developed for this project. These questions will be in self-report format (rather than interview format), administered at the conclusion of the final EMA survey block, to reduce demand characteristics.

# **Safety and Monitoring**

1. Assessment of Safety Event Report Forms

Safety reports will be assessed on the seriousness, causality, and expectedness of the event to the trial treatment(s), intervention(s), investigational medical product(s), investigational medical device(s). The following are known and expected adverse effects, harms, risks or discomforts associated with trial procedures, treatments or interventions.

1. Known Adverse Effects

There are no known adverse effects associated with this intervention. The DWM intervention is low-intensity and implements present-focused psychological strategies that have been demonstrated to improve mental health. Thus, we do not anticipate any additional risk or burden for participants.

1. Known Harms, Risks or Discomforts

Participants will be asked to respond to interview questions, and complete brief online questions, about their psychological state and mood, physical health, refugee status and their history of stressful events. This procedure may lead to transient psychological distress, however no psychological stress will be placed on participants that they do not normally experience. Administering the instruments is crucial to draw conclusions about the intervention’s efficacy and mechanisms of change. The research team has extensive experience in conducting research with refugees in Indonesia and Australia and the project Assessors have been trained in interview techniques and how to manage negative emotional reactions in participants.

Participants will be in regular contact with the research team throughout the intervention (with Structured Assessments every 3-weeks and regular facilitator calls during the DWM phase). If, at any stage, the participant experiences an undesirable emotional reaction or indicates they are experiencing significant symptoms (i.e., issues in line with our exclusion criteria, such as symptoms consistent with a severe physical or mental condition) then the trained assessor/facilitator will be able to triage the issue by either

(1) providing referral information directly to the participant so they can access more specialised services, or

(2) escalating the participant to the research team's clinical supervisor who can assess and refer as needed.

1. Adverse Events or Adverse Reactions

Adverse events (AE) are considered any untoward medical occurrence in a patient or clinical trial participant administered the intervention, which does not necessarily have a causal relationship with this treatment.

Adverse Reactions (AR) are considered untoward and unintended responses to the trial intervention related to any intervention procedures.

AEs and ARs are assessed using the safety monitoring flow chart. Those classified as "not serious" are assessed by the qualified physician/medical expert specified in section 2 of the protocol. The Qualified Physician cannot delegate this responsibility to other research personnel.

Adverse event reports must be reported to the Coordinating Principal Investigator without undue delay. All adverse event reports must be recorded in the [UNSW Safety Monitoring Register Template](https://research.unsw.edu.au/document/UNSW%20Safety%20Monitoring%20Register%20Template.xlsx).

1. Serious Adverse Events

Serious Adverse Events (SAEs) that result in or lead to one or more of the following and the event is not related to the trial intervention:

- The death of a trial participant.
- A life-threatening illness or injury involving a trial participant.
- A participant's permanent impairment of body structure or body function.
- In-patient or prolonged hospitalisation (not for a pre-existing condition or an elective surgery) of a trial participant.
- Medical or surgical intervention to prevent life-threatening illness or injury or permanent impairment to a body structure or function of a trial participant.
- Fetal distress, fetal death or congenital abnormality or birth defect.

SAE reports are classified following the safety assessment flowchart and are assessed by Sponsors Independent Medical specified in section 2 of the protocol. The Sponsors Independent Medical cannot delegate this responsibility to other research personnel. SAE reports are reported to the Coordinating Principal Investigator within 48 hours of the event occurring for multicentre clinical trials. SAR reports must be recorded in the [UNSW Safety Monitoring Register Template](https://research.unsw.edu.au/document/UNSW%20Safety%20Monitoring%20Register%20Template.xlsx).

1. Serious Adverse Reactions

A Serious Adverse Reactions (SAR) is an SAE that is related to the trial intervention. SAR reports are classified following the safety assessment flowchart and are assessed by Sponsors Independent Medical specified in section 2 of the protocol. The sponsors independent medical expert must determine whether the SAR was expected or unexpected. The Sponsors Independent Medical cannot delegate this responsibility to other research personnel.

#### **Expected Serious Adverse Reaction**

A serious adverse reaction by its nature, incidence, severity, or outcome is anticipated and identified in the current version of the intervention safety information are classified as a SAR report. SAR reports are reported to the Coordinating Principal Investigator without undue delay for multicentre clinical trials. Serious Adverse Reaction reports must be recorded in the [UNSW Safety Monitoring Register Template](https://research.unsw.edu.au/document/UNSW%20Safety%20Monitoring%20Register%20Template.xlsx).

#### **Suspected Unexpected Serious Adverse Reaction (SUSAR)**

A serious adverse reaction by its nature, incidence, severity, or outcome is unanticipated and not identified in the interventions instructions for use or safety information are classified as a SUSAR.

Fatal or life-threatening Australian SUSAR reports are reported to the Coordinating Principal Investigator, the sponsor's delegate and the approving HREC within 7 calendar days after being made aware of the case follow up information reported within a further 8 calendar days.

All other Australian SUSAR reports are to be reported to the Coordinating Principal Investigator, the sponsor's delegate and the approving HREC within 15 calendar days after being made aware of the case follow up information reported within a further 8 calendar days. SUSAR reports must be recorded in the [UNSW Safety Monitoring Register Template](https://research.unsw.edu.au/document/UNSW%20Safety%20Monitoring%20Register%20Template.xlsx).

1. Significant Safety Issue (SSI)

A safety issue that could adversely affect participants' safety or materially impact the trial's continued ethical acceptability or conduct. The Human Research Ethics Committee and Sponsor's Delegate must be notified of all significant safety issues within 15 calendar days of the sponsor instigating or being made aware of the issue**.** SSI reports must be recorded in the [UNSW Safety Monitoring Register Template](https://research.unsw.edu.au/document/UNSW%20Safety%20Monitoring%20Register%20Template.xlsx).

1. Urgent Safety Measure (USM)

A measure that is taken to eliminate an immediate hazard to a participant's health or safety. Significant safety issues where an urgent safety measure is required to be taken to eliminate an immediate hazard must be classified as a significant safety issue requiring an urgent safety measure. The Human Research Ethics Committee and the Sponsor's Delegate must be notified of any significant safety issues that meet the definition of an urgent safety measure should be notified within 72 hours. Examples include:

- a serious adverse event that could be associated with the trial procedures and that requires modification of the conduct of the trial
- a patient population hazard, such as lack of efficacy of an intervention used for the treatment of a life-threatening disease.

USM reports must be recorded in the [UNSW Safety Monitoring Register Template](https://research.unsw.edu.au/document/UNSW%20Safety%20Monitoring%20Register%20Template.xlsx).

1. Safety Assessment Flow Chart Investigational Medical Product Trials


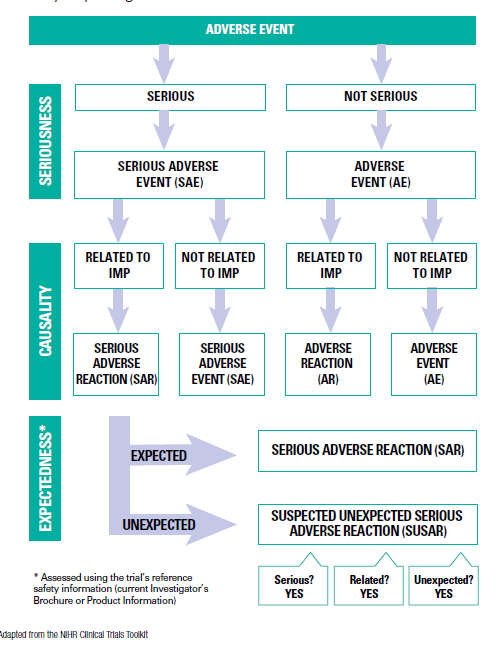


1. Register of Clinical Trial Safety Monitoring Reports

A register of all event reports assessed and classified is to be retained by the Coordinating Principal Investigator and reported to the trial sponsor annually and the HREC if required.

1. Reporting of Clinical Trial Safety Monitoring Reports

Single case reports of Adverse Events Adverse Reactions, Serious Adverse Events (SAEs), Serious Adverse Reactions (SARs), reports do not need to be reported to the UNSW Sponsor's Delegate or the HREC. All single case reports must be recorded in a safety monitoring register and are reported to the UNSW Sponsor's Delegate annually.

#### **Emerging Safety Issues**

The Trial Management Group, Trial Safety Committee or the Data Safety Monitoring Board is responsible for reviewing the safety information to identify any serious emerging safety concerns. If safety concerns are identified, this body will establish a plan to minimise the time participants may be placed at excess risk of harm. Before implementing the plan, the Trial Management Group, Trial Safety Committee or the Data Safety Monitoring Board must seek the advice of the human research ethics committee and sponsor's delegate.

#### **Annual assessment of safety**

The following information must be provided in a report to the sponsors delegate annually:

- Documented evidence that the Trial Management Group, Trial Safety Committee, or the Data Safety Monitoring Board (e.g. meeting minutes) confirmed that regular safety reviews occurred.
- Analysis of the trial intervention(s) and its implications for participants considering all available safety data and relevant clinical or non-clinical studies results.
- Any reports of emerging safety issues and a description of any measures taken or proposed to minimise risks.
- A copy of the safety monitoring register.

# **Non-compliance, Protocol Deviation and Serious Breaches of Good Clinical Practice**

## **Protocol Deviation**

A protocol deviation is defined as any breach, divergence or departure from the requirements of Good Clinical Practice, the clinical trial protocol, the clinical trial standard operating procedures, or the human ethics approval that does not have a significant impact on the continued safety or rights of participants or the reliability and robustness of the data generated in the research or clinical trial. Protocol deviations are events that do not occur persistently or systematically and do not potentially result in participant harms. Examples of protocol deviations include but are not limited to:

- Deviations because of participant adherence to the protocol, including rescheduled study visits, participants refusal to complete scheduled research activities or failure to complete self-report questionnaires required by the study protocol.
- Blood samples obtained or clinical trial testing occurring at times close to, but not precisely at the time points specified in the protocol.
- The completion of consent forms, safety monitoring report, case report forms or data collection tools in a manner that is not consistent with the protocol instructions or failure to make reports within the required reporting timeframes.
- Administration of the clinical trial investigational medical product or device in a manner that is not consistent with the manufacturer's instructions for use.
- Use of an unapproved version of the participant information statement or recruitment of participants using unapproved recruitment procedures.
- Inclusion of a participant that does not meet the inclusion criteria.
- An urgent safety measure must be taken to eliminate an immediate hazard to a participant's health or safety.

## **Serious Breach of Good Clinical Practice**

A serious breach is defined as a breach of Good Clinical Practice, the clinical trial protocol, the clinical trial standard operating procedures, or the human ethics approval that is likely to affect to a significant degree the safety or rights of participants or the reliability and robustness of the data generated in the clinical trial. Examples of serious breaches include but are not limited to:

- Persistent or systematic non-compliance with the instructions for completing consent forms, safety monitoring forms, case report forms or data collection tools that result in continued missed or incomplete data collection.
- Failure to record or report adverse events, serious adverse events, suspected unexpected serious adverse reactions, significant safety issues where urgent safety measures were implemented.
- Failure to conduct clinical trial procedures following the clinical trial delegation log.
- Widespread and uncontrolled use of protocol waivers affecting eligibility criteria, which leads to harm to trial subjects.
- Failure to report investigational medical product or device defects to the clinical trial sponsor or any relevant regulatory body.
- Failure to conduct research following the issued approvals, permits or licences by required laws, regulations, disciplinary standards, and UNSW policies relating to the responsible or safe conduct of research.
- Concealing or facilitating breaches (or potential breaches) of the Research Code by others.
- Researching without the requisite approvals, permits or licences required by laws, regulations, disciplinary standards, and UNSW policies related to the responsible or safe conduct of research.
- Failure to conduct research as approved by an ethics review body where that conduct leads to (or has the potential to) results in participant harms.
- Researching without ethics approval as required by the National Statement on Ethical Conduct in Human Research where that conduct leads to (or has the potential to) result in participant harms.
- Any breaches as outlined in the UNSW Research Misconduct Procedure or the Australian Code for responsible conduct of research that leads to (or can potentially) result in participant harms.

## **Reporting Protocol Deviations**

- Protocol deviations occurring at a site must be documented in site files and reported by the principal site investigator to the Coordinating Principal Investigator.
- The Coordinating Principal Investigator must review the protocol deviation and the clinical trial protocol to establish the corrective actions and preventative steps to prevent the deviation from reoccurring.
- The protocol deviation and corrective action plan must be reported to the UNSW Sponsor's Delegate by the Coordinating Principal Investigator or Coordinating Research Team using the protocol deviation report form.

## **Reporting of a Serious Breach**

- The Principal Investigator must report a serious breach occurring at a participating site to the Coordinating Principal Investigator within a specified timeframe.
- The Coordinating Principal Investigator must review the serious breach, along with the clinical trial protocol, to develop a Corrective and Preventive Action (CAPA) that defines the steps to prevent the serious breach from reoccurring.
- The serious breach report and the CAPA must be provided to the approving HREC, and the UNSW sponsors delegate for review and approval.

## **Reporting of Serious Breaches by Third Parties**

- A Suspected Breach is a report judged by the reporter as a possible serious breach but has yet to be formally confirmed as a serious breach by the sponsor.
- A Suspected Breach form must be completed when a third party (e.g., individual/institution) wishes to report a suspected breach of Good Clinical Practice or the protocol and should be reported directly to the reviewing HREC without reporting through the sponsor.
- Recording of Protocol Deviation and Serious Breach Reports
- A register of protocol deviation and serious breach reports must be recorded. Written records and copies of documentation sent to the sponsor must be retained in the Investigator Site File.
- Copies of protocol deviation and serious breach reports must be recorded, written records and copies of documentation sent to the sponsor, referrals made to the HREC or establishing whether a breach of the Australian Code for Responsible conduct of research must be retained in the Master Site File.

# **Review of a Protocol Deviation and a Serious Breach**

- The UNSW Sponsor's Delegate will review reports to establish whether the event meets the definition of a protocol deviation or serious breach,  establish whether the proposed CAPA is appropriate and establish whether there is or will be ongoing impact reliability and robustness of the data generated.
- The UNSW Sponsor's Delegate will seek advice from the approving HREC on the corrective and preventive actions.
- Protocol deviation or serious breach reports where a UNSW researcher, staff or student is responsible for the protocol deviation or the serious breach will be reviewed as per the [UNSW Research Misconduct Procedure](https://www.gs.unsw.edu.au/policy/documents/researchmisconductproc.pdf) to establish a breach of the [UNSW Research Code of Conduct](https://www.gs.unsw.edu.au/policy/documents/researchcode.pdf) has occurred.
- Protocol deviation or serious breach reports where the UNSW Sponsor's Delegate determines that site personnel are responsible for a protocol deviation or the serious breach will be referred onto their responsible institution for review under their Research Misconduct procedures to establish whether a breach of the [Australian Research Code for the Responsible Conduct of Research](https://www.nhmrc.gov.au/about-us/publications/australian-code-responsible-conduct-research-2018) has occurred.

# **Statistics**

Data collected throughout the study will be analysed using hierarchical linear modelling or linear mixed models. Both intention-to-treat analysis (ITT; using all randomised participants) and completers’ (PP) analysis will be used.

Primary study parameters:

The main conclusion for study aim 1 (testing the efficacy of the DWM intervention, according to K10 scores) will be based on the ITT analysis of the primary outcome. A secondary analysis of the primary outcome will also be presented using the PP population. To estimate the treatment effect, data from Structured Assessments 1-4 will be used. A linear mixed model will be employed for the primary endpoint analysis, which will have treatment as fixed effects, baseline measurement of primary endpoint as covariate, and subject as random effects. The mean difference between two treatment arms at each visit/time together with its 95% confidence interval will be derived from the mixed model. Covariate-adjusted mixed model of primary endpoint will also be performed by adding pre-specified covariates at baseline (gender, age, education, traumatic experiences, and severity of symptoms) into the above model.

- Secondary study parameters: A linear mixed model as mentioned for the primary outcome analysis will be carried out for analysing the secondary outcomes (e.g., PTSD, Suicidal Ideation, Self-Identified Problems, Wellbeing, Functional Impairment, Digital Markers).

The main conclusion for study aim 2 (testing the mechanisms of change within the DWM intervention) will be based on the PP analysis, to ensure that only data from participants who received a sufficient ‘dose’ of the intervention are analysed. Cross-lagged panel analysis will be used to determine the temporal sequencing of psychological factors over the course of the intervention period, to test whether the hypothesised processes were mechanisms of change driving the intervention effects [hypothesis 2].

- The hypothesised mechanisms are: psychological flexibility (primary), emotion regulation, social satisfaction and functioning, and intolerance of uncertainty.

Missing data: Missing data will be treated as missing at random (MAR). No imputations of missing values will be made, as multilevel models can deal with missing data (Singer & Willett, 2003).

Other study parameters:

- Treatment fidelity: In order to determine whether the intervention-as-implemented does not differ from the intervention-as-designed, fidelity checklists filled out by research assistants independent to the treatment facilitation team will be completed for a random sample, stratified on facilitator, of sessions/participants. Treatment fidelity will be analysed as a manipulation check.

Interim analysis (if applicable): Interim analyses will be considered in case safety issues are (suspected to be) violated.

# **Data Ownership**

All research data collected during this trial is governed and handled following the Research Data Governance and Materials Handling [policy](https://www.gs.unsw.edu.au/policy/documents/researchdatagovernancepolicy.pdf). UNSW, rather than any individual or Organisational Unit, is the Custodian of data and materials and any information derived from the data. Original research data and primary materials generated in the research conducted at the University will be owned and retained by the University subject to any contractual, statutory, ethical, or funding body requirements.

# **Handling and Reporting Data**

Principal Investigators are responsible for maintaining adequate and accurate source documents and trial records that include all pertinent observations on each site's trial subjects. Source data must be attributable, legible, contemporaneous, original, accurate, and complete.

Trial subjects will be assigned a participant ID, and data will be reported using the [case report form]. Data reported on the [case report form], derived from source documents, should be consistent with the source documents, or the discrepancies must be explained. Any change or correction to a [case report form] should be dated, initialled, and explained (if necessary) and should not obscure the original entry (i.e., an audit trail should be maintained); this applies to both written and electronic changes or corrections.

1. Direct Access to Source Data and Documents

Site principal investigator(s) and institution(s) will permit trial-related monitoring, audits, IRB/IEC review, and regulatory inspection(s), providing direct access to source data/documents.

# **Monitoring Quality Control and Quality Assurance**

The Coordinating Principal Investigator and Principal Investigator(s) 'responsibility are to monitor the clinical trial. The Coordinating Principal Investigator and Principal Investigator(s) are responsible for undertaking or participating in site initiation or protocol-specific training before recruitment and data collection commences. A monitoring report demonstrating regular compliance monitoring with the clinical trial protocol, procedures, and HREC approval is provided to the UNSW Sponsor's Delegate annually.

Root, cause, analysis reports are to be completed by the Coordinating Principal Investigator for reports of non-compliance and serious breaches. A corrective and preventative action plan must be developed and actioned for any reports of non-compliance and serious breaches.

# **Clinical Trial Research Agreement**

The Coordinating Principal investigators must ensure that agreements are executed at each of the following sites before site initiation, recruitment, and data collection commences.

# **Research Governance Site Authorisation**

Site authorisation is to be obtained, or if a research site is added, a site authorisation letter from the delegated authority of an institution responsible for any participating site is obtained. It is to be stored as a GCP essential document before participants are recruited at a participating site.

# **Good Clinical Practice Requirements**

It is recommended that the Coordinating and Principal Investigators' ensure that all investigators and trial-related staff have current Good Clinical Practice Training. Once completed, the evidence of training confirmation is to be stored as a GCP essential document.

It is the responsibility of the Coordinating and Principal Investigators to familiarise themselves with the requirements of the [Guideline for Good Clinical Practice (E6, R2)](https://database.ich.org/sites/default/files/E6_R2_Addendum.pdf)

# **Essential Documents for the Conduct of a Clinical Trial**

All essential documents referred to in section 8.2 of the [Guideline for Good Clinical Practice (E6, R2)](https://database.ich.org/sites/default/files/E6_R2_Addendum.pdf)   are to be retained by all trial investigators.

# **Clinical Trial Delegation and Responsibilities Log**

| **Protocol / Study Number:** |  | **Sponsor Name:** |  |
| --- | --- | --- | --- |
| **Principal Investigator Name:** |  | **Site Number:** |  |
| **Site Name (if applicable)** |  | | |

***THIS FORM IS TO BE COMPLETED BY ALL PERSONNEL INVOLVED IN THE STUDY AFTER RECEIVING PROPER STUDY TRAINING AND BEFORE TAKING PART IN ANY STUDY ACTIVITIES**

**Principal Investigator (PI)**

By signing, I confirm/acknowledge that the tasks listed below will only be delegated to appropriately trained, skilled and qualified staff. I will remain responsible for the overall study conduct and reported data, ensuring study oversight. All associates, colleagues, and employees assisting in the conduct of the study are informed about their obligations and have not performed any study tasks before appropriate delegation and completion of appropriate training. Mechanisms are in place to ensure that site staff receives the appropriate information and training throughout the study and that a 2-way communication channel exists between staff and self. Any changes in staff or delegation in staff will be recorded promptly.

| **Name** | **Principal Investigator’s Signature** | **Initials** | **Start**  **(dd/mmm/yyyy)** | **End**  **(dd/mmm/yyyy)**  **(complete only if prior to end of study)** |
| --- | --- | --- | --- | --- |
|  |  |  |  |  |
|  |  |  |  |  |

Site Staff

| **Name** | **Signature** | **Initials** | **Study Role** | **Key Study Task(s)**  **(choose from list below)** | **Start**  **(dd/mmm/yyyy)** | **End**  **(dd/mmm/yyyy) (complete only if prior to end of study)** | **PI Initials & Date**  **(dd/mmm/yyyy)** |
| --- | --- | --- | --- | --- | --- | --- | --- |
|  |  |  |  |  |  |  | __/___/_____ |
|  |  |  |  |  |  |  | __/___/_____ |
|  |  |  |  |  |  |  | __/___/_____ |
|  |  |  |  |  |  |  | __/___/_____ |
|  |  |  |  |  |  |  | __/___/_____ |
|  |  |  |  |  |  |  | __/___/_____ |
|  |  |  |  |  |  |  | __/___/_____ |
|  |  |  |  |  |  |  | __/___/_____ |
|  |  |  |  |  |  |  | __/___/_____ |
|  |  |  |  |  |  |  | __/___/_____ |
|  |  |  |  |  |  |  | __/___/_____ |
|  |  |  |  |  |  |  | __/___/_____ |

| **Comments:** |
| --- |
|  |

| **Electronic Signature Declaration for Principal Investigator and Site Staff**   1. My electronic signature as it applies to entering electronic data or signing records in sponsor-owned or sponsor -outsourced computer systems is the legally binding equivalent of my handwritten signature. 2. I will not share password(s) assigned to me for this study with any other persons. |
| --- |

| **Principal Investigator's End of Study Declaration**  I hereby confirm that the above information is accurate and complete, and that I authorised the delegation of study-related tasks to each individual as listed above.  **Principal Investigator’s Signature:** **Date:** |
| --- |

**Task Key:**

| 1. Obtain informed consent * | 12. Sample collection |
| --- | --- |
| 2. Subject selection/recruitment* | 13. Sample processing and/or shipment |
| 3. Confirm eligibility (review inclusion/exclusion criteria)* | 14. Evaluate study-related test results * |
| 4. Obtain medical history (source documents) | 15. Use IWRS/IVRS |
| 5. Perform physical exam* | 16. Make entries/corrections on (e)CRFs |
| 6. Conduct study visit procedure as outlined in the protocol* | 17. Sign- off (e)CRFs* |
| 7. Make study-related medical decisions* | 18. Maintain essential documents |
| 8. Assess AEs/SAEs* | 19. Perform study-related assessments as per protocol * |
| 9. Dispense study drug* | 20. Complete company- specific log ( if applicable) |
| 10. Perform drug accountability | 21. Other (specify)____________________________________________ |
| 11. Study drug storage and temperature monitoring | 22. Other (specify) ____________________________________________ |

*These tasks may only be performed by qualified individual as permitted by local law, medical or standard of care practices, or applicable required training as per job description or designation.

# **Safety Monitoring Register Template**

- [UNSW Safety Monitoring Register Template](https://research.unsw.edu.au/document/UNSW%20Safety%20Monitoring%20Register%20Template.xlsx)
- [UNSW Adverse Event or Incident Event Case Report Form](https://research.unsw.edu.au/document/Adverse%20Event%20Incident%20Report%20Form%20September%202019%20.docx) Example.

# References

Abbas, A., Yadav, V., Smith, E., Ramjas, E., Rutter, S. B., Benavidez, C., ... & Galatzer-Levy, I. R. (2021). Computer vision-based assessment of motor functioning in schizophrenia: use of smartphones for remote measurement of schizophrenia symptomatology. Digital biomarkers, 5(1), 29-36.

Abbas, A., Hansen, B. J., Koesmahargyo, V., Yadav, V., Rosenfield, P. J., Patil, O., ... & Galatzer-Levy, I. R. (2022). Facial and vocal markers of schizophrenia measured using remote smartphone assessments: Observational study. *JMIR formative research*, *6*(1), e26276.

Acarturk, C., Kurt, G., Ilkkursun, Z., Uygun, E., & Karaoglan-Kahilogullari, A. (2022). “Doing What Matters in Times of Stress” to Decrease Psychological Distress During COVID-19: A Randomised Controlled Pilot Trial. *Intervention Journal of Mental Health and Psychosocial Support in Conflict Affected Areas*, *20*(2), 170-178.

Acarturk, C., Uygun, E., Ilkkursun, Z., Yurtbakan, T., Kurt, G., Adam-Troian, J., ... & Fuhr, D. C. (2022). Group problem management plus (PM+) to decrease psychological distress among Syrian refugees in Turkey: a pilot randomised controlled trial. *BMC psychiatry*, *22*, 1-11.

Ashworth, M., Shepher, M., Christey, J., Matthews, V., Wright, K., Parmentier, H., … Godfrey, E. (2004). A client-generated psychometric instrument: the development of “PSYCHLOPS.” Counseling and Psychotherapy Research, 4(2), 27–32. https://doi.org/10.1093/astrogeo/atw101

Baik, S. Y., & Newman, M. G. (2023). The transdiagnostic use of worry and rumination to avoid negative emotional contrasts following negative events: A momentary assessment study. *Journal of Anxiety Disorders*, *95*, 102679.

Blackmore, R., Gray, K. M., Boyle, J. A., Fazel, M., Ranasinha, S., Fitzgerald, G., ... & Gibson-Helm, M. (2020). Systematic review and meta-analysis: the prevalence of mental illness in child and adolescent refugees and asylum seekers. *Journal of the American Academy of Child & Adolescent Psychiatry*, *59*(6), 705-714.

Bond, F. W., Hayes, S. C., Baer, R. A., Carpenter, K. M., Guenole, N., Orcutt, H. K., Waltz, T., & Zettle, R. D. (2011). Preliminary psychometric properties of the Acceptance and Action Questionnaire – II: A revised measure of psychological inflexibility and experiential avoidance. Behavior Therapy, 42, 676–688.

Bryant, R. A. (2023). Scalable interventions for refugees. *Cambridge Prisms: Global Mental Health*, *10*, e8.

Carleton, R. N., Norton, M. P. J., & Asmundson, G. J. (2007). Fearing the unknown: A short version of the Intolerance of Uncertainty Scale. *Journal of anxiety disorders*, *21*(1), 105-117.

Cloos, L., Ceulemans, E., & Kuppens, P. (2023). Development, validation, and comparison of self-report measures for positive and negative affect in intensive longitudinal research. *Psychological Assessment*, *35*(3), 189.

Cooper, P., Osborn, M., Gath, D., & Feggetter, G. (1982). Evaluation of a modified self-report measure of social adjustment. *The British Journal of Psychiatry, 141*(1), 68-75.

Cuijpers, P., Muñoz, R. F., Clarke, G. N., & Lewinsohn, P. M. (2009). Psychoeducational treatment and prevention of depression: the “Coping with Depression” course thirty years later. *Clinical psychology review*, *29*(5), 449-458.

Cuijpers, P., Donker, T., van Straten, A., Li, J., & Andersson, G. (2010). Is guided self-help as effective as face-to-face psychotherapy for depression and anxiety disorders? A systematic review and meta-analysis of comparative outcome studies. *Psychological medicine*, *40*(12), 1943-1957.

Czachowski, S., Seed, P., Schofield, P., & Ashworth, M. (2011). Measuring psychological change during cognitive behaviour therapy in primary care: a Polish study using ‘PSYCHLOPS’(Psychological Outcome Profiles). *PLoS One*, *6*(12), e27378.

Ehring, T., Zetsche, U., Weidacker, K., Wahl, K., Schönfeld, S., & Ehlers, A. (2011). The Perseverative Thinking Questionnaire (PTQ): Validation of a content-independent measure of repetitive negative thinking. *Journal of behavior therapy and experimental psychiatry, 42*(2), 225-232.

Epping‐Jordan, J. E., Harris, R., Brown, F. L., Carswell, K., Foley, C., García‐Moreno, C., ... & van Ommeren, M. (2016). Self‐Help Plus (SH+): a new WHO stress management package. *World Psychiatry*, *15*(3), 295.

Fassaert, T., de Wit, M. A. S., Tuinebreijer, W. C., Wouters, H., Verhoeff, A. P., Beekman, A. T. F., & Dekker, J. (2009). Psychometric properties of an interviewer-administered version of the Kessler Psychological Distress scale (K10) among Dutch, Moroccan and Turkish respondents. International Journal of Methods in Psychiatric Research, 18(3), 159–168.

French, K., Golijani-Moghaddam, N., & Schröder, T. (2017). What is the evidence for the efficacy of self-help acceptance and commitment therapy? A systematic review and meta-analysis. *Journal of Contextual Behavioral Science*, *6*(4), 360-374.

Fuhr, D. C., Acarturk, C., McGrath, M., Ilkkursun, Z., Sondorp, E., Sijbrandij, M., ... & Roberts, B. (2020). Treatment gap and mental health service use among Syrian refugees in Sultanbeyli, Istanbul: a cross-sectional survey. *Epidemiology and psychiatric sciences*, *29*, e70.

Galatzer-Levy, I., Abbas, A., Ries, A., Homan, S., Sels, L., Koesmahargyo, V., ... & Kleim, B. (2021). Validation of visual and auditory digital markers of suicidality in acutely suicidal psychiatric inpatients: proof-of-concept study. *Journal of medical Internet research*, *23*(6), e25199.

Golijani-Moghaddam, N., Morris, J. L., Bayliss, K., & Dawson, D. L. (2023). The CompACT-10: Development and validation of a comprehensive assessment of acceptance and commitment therapy processes short-form in representative UK samples. *Journal of Contextual Behavioral Science*, *29*, 59-66.

Hacker, T., Stone, P., & MacBeth, A. (2016). Acceptance and commitment therapy–do we know enough? Cumulative and sequential meta-analyses of randomized controlled trials. *Journal of affective disorders*, *190*, 551-565.

Hayes, S. C., Levin, M. E., Plumb-Vilardaga, J., Villatte, J. L., & Pistorello, J. (2013). Acceptance and commitment therapy and contextual behavioral science: Examining the progress of a distinctive model of behavioral and cognitive therapy. *Behavior therapy*, *44*(2), 180-198.

Heckendorf, H., Lehr, D., Ebert, D. D., & Freund, H. (2019). Efficacy of an internet and app-based gratitude intervention in reducing repetitive negative thinking and mechanisms of change in the intervention's effect on anxiety and depression: results from a randomized controlled trial.*Behaviour research and therapy, 119*, 103415.

Héðinsson, H., Kristjánsdóttir, H., Ólason, D. Þ., & Sigurðsson, J. F. (2013). A validation and replication study of the patient-generated measure PSYCHLOPS on an Icelandic clinical population. *European Journal of Psychological Assessment*.

Kessler, R. C., Andrews, G., Colpe, L. J., Hiripi, E., Mroczek, D. K., Normand, S.-L. T., … Zaslavsky, A. M. (2002). Short screening scales to monitor population prevalences and trends in non-specific psychological distress. Psychological Medicine, 32, 959–976. https://doi.org/10.1017/ S0033291702006074

Koch, T., Liedl, A., Takano, K., & Ehring, T. (2020). Daily worry in trauma-exposed Afghan refugees: Relationship with affect and sleep in a study using ecological momentary assessment. *Cognitive Therapy and Research*, *44*, 645-658.

Lakin, D. P., Cooper, S. E., Andersen, L., Brown, F. L., Augustinavicius, J. L., Carswell, K., ... & Tol, W. A. (2023). Psychological flexibility in South Sudanese female refugees in Uganda as a mechanism for change within a guided self-help intervention. *Journal of Consulting and Clinical Psychology*, *91*(1), 6.

Meaney, T., Yaday, V., Galatzer-Levy, I., & Bryant, R. (Under Review). Different emotional challens underpinning regulation of affective states.

Medland, H., De France, K., Hollenstein, T., Mussoff, D., & Koval, P. (2020). Regulating emotion systems in everyday life. *European Journal of Psychological Assessment*.

Moeller, S. B., Larsen, P. V., Arendt, I. M. T., Ehring, T., Reinholt, N., Hvenegaard, M., ... & Arnfred, S. (2023). Validation of the Danish Version of Perseverative Thinking Questionnaire (PTQ)–Introducing the PTQ Short Version. *Psychological Test Adaptation and Development*, *4,* 310-318.

Mollica, R. F., Caspi-Yavin, Y., Bollini, P., Truong, T., Tor, S., & Lavelle, J. (1992). The Harvard Trauma Questionnaire: Validating a cross-cultural instrument for measuring torture, trauma, and posttraumatic stress disorder in Indochinese refugees. *The Journal of nervous and mental disease*, *180*(2), 111-116.

Morris, J. (2019). *Development and validation of a short form of the Comprehensive assessment of Acceptance and Commitment Therapy processes (CompACT-SF)* (Doctoral dissertation, University of Nottingham).

Nickerson, A., Bryant, R. A., Steel, Z., Silove, D., & Brooks, R. (2010). The impact of fear for family on mental health in a resettled Iraqi refugee community. *Journal of psychiatric research*, *44*(4), 229-235.

Nickerson, A., Bryant, R. A., Schnyder, U., Schick, M., Mueller, J., & Morina, N. (2015). Emotion dysregulation mediates the relationship between trauma exposure, post-migration living difficulties and psychological outcomes in traumatized refugees. *Journal of Affective Disorders*, *173*, 185-192.

Nickerson, A., Liddell, B. J., Keegan, D., Edwards, B., Felmingham, K. L., Forbes, D., ... & Bryant, R. A. (2019). Longitudinal association between trust, psychological symptoms and community engagement in resettled refugees. *Psychological medicine*, *49*(10), 1661-1669.

Nickerson, A., Hoffman, J., Keegan, D., Kashyap, S., Tricesaria, D., Pestalozzi, Z., ... & Liddell, B. J. (2022). Context, coping, and mental health in refugees living in protracted displacement. *Journal of Traumatic Stress*, *35*(6), 1769-1782.

Nickerson, A., Hoffman, J., Keegan, D., Kashyap, S., Argadianti, R., Tricesaria, D., ... & Liddell, B. (2023). Intolerance of uncertainty, posttraumatic stress, depression, and fears for the future among displaced refugees. *Journal of Anxiety Disorders*, *94*, 102672.

Ong, C. W., Barthel, A. L., & Hofmann, S. G. (2024). The relationship between psychological inflexibility and well-being in adults: a meta-analysis of the Acceptance and Action Questionnaire. Behavior Therapy, 55(1), 26-41.

Patel, V., Araya, R., Chowdhary, N., King, M., Kirkwood, B., Nayak, S., … Weiss, H. A. (2008). Detecting common mental disorders in primary care in India: a comparison of five screening questionnaires. Psychological Medicine, 38(2), 221–228. https://doi.org/10.1017/S0033291707002334

Schick, M., Zumwald, A., Knöpfli, B., Nickerson, A., Bryant, R. A., Schnyder, U., ... & Morina, N. (2016). Challenging future, challenging past: the relationship of social integration and psychological impairment in traumatized refugees. *European journal of psychotraumatology*, *7*(1), 28057.

Schultebraucks, K., Yadav, V., Shalev, A. Y., Bonanno, G. A., & Galatzer-Levy, I. R. (2022). Deep learning-based classification of posttraumatic stress disorder and depression following trauma utilizing visual and auditory markers of arousal and mood. *Psychological Medicine*, *52*(5), 957-967.

Schweitzer, R., Melville, F., Steel, Z., & Lacherez, P. (2006). Trauma, post-migration living difficulties, and social support as predictors of psychological adjustment in resettled Sudanese refugees. *Australian & New Zealand Journal of Psychiatry*, *40*(2), 179-187.

Scoglio, A. A., Reilly, E. D., Girouard, C., Quigley, K. S., Carnes, S., & Kelly, M. M. (2022). Social functioning in individuals with post-traumatic stress disorder: A systematic review. *Trauma, Violence, & Abuse, 23*(2), 356-371.

Silove, D., Sinnerbrink, I., Field, A., Manicavasagar, V., & Steel, Z. (1997). Anxiety, depression and PTSD in asylum-seekers: Associations with pre-migration trauma and post-migration stressors. *The British Journal of Psychiatry*, *170*, 351.

Singer, J. D., & Willett, J. B. (2003). *Applied longitudinal data analysis: Modeling change and event occurrence*. Oxford university press.

Steel, Z., Silove, D., Bird, K., McGorry, P., & Mohan, P. (1999). Pathways from war trauma to posttraumatic stress symptoms among Tamil asylum seekers, refugees, and immigrants. *Journal of traumatic stress*, *12*, 421-435.

Steel, Z., Silove, D., Brooks, R., Momartin, S., Alzuhairi, B., & Susljik, I. N. A. (2006). Impact of immigration detention and temporary protection on the mental health of refugees. *The british journal of psychiatry*, *188*(1), 58-64.

Sulaiman-Hill, C. M., & Thompson, S. C. (2010). Selecting instruments for assessing psychological wellbeing in Afghan and Kurdish refugee groups. BMC Research Notes, 3, 237. https://doi.org/10.1186/1756-0500-3-237

Tissue, A., Specker, P., Hoffman, J., Uppal, S., Cloitre, M., Neuner, F., ... & Nickerson, A. (2023). Skills training in affective and interpersonal regulation for refugees integrated with narrative exposure therapy: A case study on the treatment of PTSD and emotion dysregulation for refugees and asylum-seekers. *Clinical Case Studies*, *22*(3), 285-306.

Tol, W. A., Leku, M. R., Lakin, D. P., Carswell, K., Augustinavicius, J., Adaku, A., ... & van Ommeren, M. (2020). Guided self-help to reduce psychological distress in South Sudanese female refugees in Uganda: a cluster randomised trial. *The Lancet Global Health*, *8*(2), e254-e263.

Topp, C. W., Østergaard, S. D., Søndergaard, S., & Bech, P. (2015). The WHO-5 Well-Being Index: a systematic review of the literature. *Psychotherapy and psychosomatics*, *84*(3), 167-176.

Ustun, T. B., Kostanjesek, N., Chatterji, S., Rehm, J., & World Health Organization. (2010). Measuring health and disability: manual for WHO Disability Assessment Schedule (WHODAS 2.0)/edited by TB Üstün, N. Kostanjsek, S. Chatterji, J. Rehm. In *Measuring health and disability: manual for WHO Disability Assessment Schedule (WHODAS 2.0)/edited by TB Üstün, N. Kostanjsek, S. Chatterji, J. Rehm*.

Van Spijker, B. A., Batterham, P. J., Calear, A. L., Farrer, L., Christensen, H., Reynolds, J., & Kerkhof, A. J. (2014). The Suicidal Ideation Attributes Scale (SIDAS): Community‐based validation study of a new scale for the measurement of suicidal ideation. *Suicide and Life‐Threatening Behavior*, *44*(4), 408-419.

Weathers, F., Blake, D., Schnurr, P., Kaloupek, D., Marx, B., & Keane, T. (2013). The Life Events Checklist for DSM-5 (LEC-5). Retrieved March 3, 2017, from http://www.ptsd.va.gov/professional/assessment/te-measures/life_events_checklist.asp

Weissman, M. M., & Bothwell, S. (1976). Assessment of social adjustment by patient self-report. *Archives of general psychiatry, 33*(9), 1111-1115.

WHO. (2010a). Measuring Health and Disability: Manual for WHO Disability Assessment Schedule WHODAS 2.0. (T. B. Üstun, N. Kostansjek, S. Chatterji, & J. Rehm, Eds.), World Health Organization. Geneva: WHO. Retrieved from https://books.google.com/books?hl=en&lr=&id=h9fhLNiaRTgC&pgis=1

WHO. (2010b). World Health Organization Disability Assessment Schedule II (WHODAS II). Geneva, Switzerland: World Health Organization.

WHO. (2013). Guidelines for the management of conditions specifically related to stress. Geneva.

WHO. (2016). Problem Management Plus (PM+): Individual psychological help for adults impaired by distress in communities exposed to adversity. Geneva: WHO.

# **
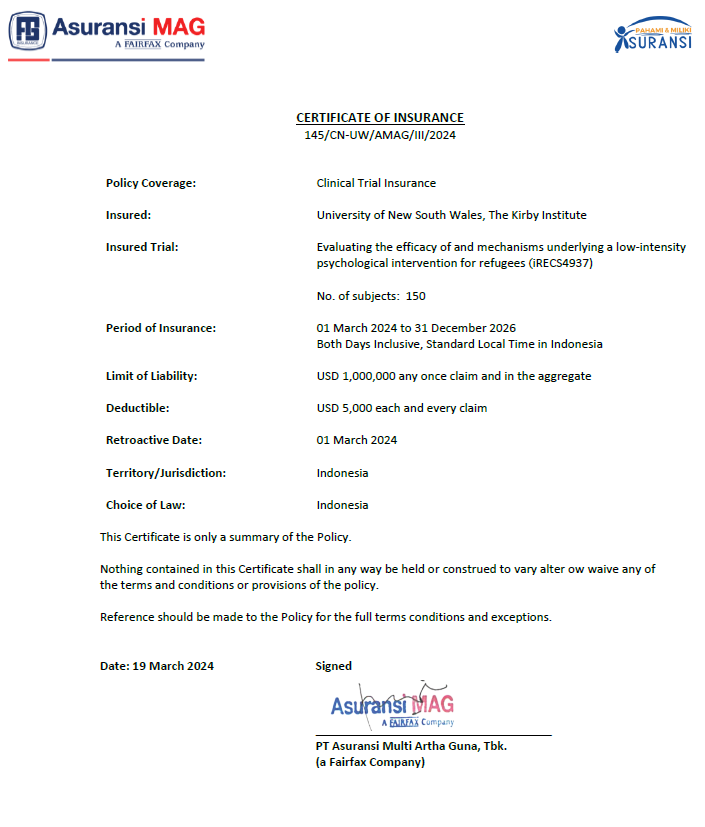
**Certificate of Insurance
