# Supplementary material for: Efficacy of a World Health Organization–Guided Self-Help Intervention for Reducing Psychological Distress in Afghan Refugees: Randomized Controlled Trial
Source: JMIR Ment Health. 2026 May 20;13:e89928. doi: 10.2196/89928 (PMC13189532; doi:10.2196/89928)
Supplement: Multimedia Appendix 2 [file mental-v13-e89928-s002.docx]

# **Table S1.** Abbreviated Enhancing Assessment of Common Therapeutic Factors scale.

| 1. How well did the facilitator demonstrate **non-verbal communication and active listening skills**? | (0) NOT ASSESSED = this factor was not assessed because it was not applicable or because it did not emerge in the session. (1) NEEDS IMPROVEMENT = the facilitator engages in other activities such as answering the door or call or laughs at the participant, or shows negative facial expressions.  (2) DONE PARTIALLY =the facilitator makes some, but not consistent, eye contact through video image (e.g., looking in the general direction of the participant); uses some body language to express interest; appears artificial  (3) DONE WELL= the facilitator makes appropriate eye contact throughout interaction; smiles when appropriate; sets up clear visibility on the camera (e.g., no backlight or unclear camera positioning); if necessary, helps participant set up audio/video for clearer communication; use various body languages; leans in to show interest; use of ‘uh-huh’, ‘hmm’ or other culturally appropriate non-lexical utterances to signal interest |
| --- | --- |
| 2.How well did the facilitator demonstrate **verbal communication skills**? - Rating option 1 was taken from EQUIP, and the rest is from original ENACT. | (0) NOT ASSESSED = this factor was not assessed because it was not applicable or because it did not emerge in the session. (1) NEEDS IMPROVEMENT = the facilitator interrupts participant; asks many suggestive or leading closed-ended questions; corrects participants; uses culturally and age-inappropriate language and terms.  (2) DONE PARTIALLY =the facilitator uses open-ended questions but does not explore topics further or offer summaries for participant reflection. (3) DONE WELL= the facilitator uses open-ended questions, summarizes and clarifies statements, e.g., “What happened? Tell me more.” |
| 3.How well did the facilitator **explain and promote confidentiality**? | (0) NOT ASSESSED = this factor was not assessed because it was not applicable or because it did not emerge in the session. (1) NEEDS IMPROVEMENT = the facilitator does not address confidentiality or minimizes participant's concern about confidentiality.  (2) DONE PARTIALLY = the facilitator tells participant that everything is confidential without explaining exceptions such as harm to self or others, or the facilitator states everything is confidential while conducting calls in a non-private setting. (3) DONE WELL=the facilitator explains that all facilitator-participant discussions are confidential with the exception of harm to self and others, and the facilitator adjusts conversation topics based on private or non-private setting |
| 4.How well did the facilitator **build rapport and use self-disclosure**? | (0) NOT ASSESSED = this factor was not assessed because it was not applicable or because it did not emerge in the session. (1) NEEDS IMPROVEMENT = the facilitator does not introduce him/herself or attempt to make the participant feel comfortable or the facilitator dominates the session talking about his/her own experiences. (2) DONE PARTIALLY = the facilitator introduces him/herself but does not help the participant feel comfortable through small talk and informal conversation or the facilitator disclosure but it is not related to participant experience or needs. (3) DONE WELL=the facilitator introduces him/herself, tries to make the participant feel comfortable and disclosure focuses on participant needs. |
| 5.How well did the facilitator **explore and normalise feelings**? | (0) NOT ASSESSED = this factor was not assessed because it was not applicable or because it did not emerge in the session. (1) NEEDS IMPROVEMENT = the facilitator does not ask about participant’s feelings or the facilitator is judgmental/critical about participant’s emotions and feelings (e.g., “You shouldn’t feel that way”, “You should stop thinking or feeling that.”)  (2) DONE PARTIALLY = the facilitator asks about feelings but does not normalize/validate or does not explore feelings in detail with participant. (3) DONE WELL = the facilitator explains that the participant’s feelings in context and if appropriate, feelings are expected for a person in his/her situation. |
| 6.How well did the facilitator **demonstrate empathy, warmth, & genuineness**? | (0) NOT ASSESSED = this factor was not assessed because it was not applicable or because it did not emerge in the session. (1) NEEDS IMPROVEMENT = the facilitator does not ask about harm to self or others. (2) DONE PARTIALLY =the facilitator asks about harm to self or others, but does not explain that he/she needs to inform the clinical supervisor immediately. (3) DONE WELL = the facilitator asks about harm to self or others and informs the clinical supervisor either immediately after the call or during the call. |
| 7.How well did the facilitator assess **harm to self, harm to others, harm from others and develop collaborative response plan**? | (0) NOT ASSESSED = this factor was not assessed because it was not applicable or because it did not emerge in the session. (1) NEEDS IMPROVEMENT = the facilitator is critical, hostile, or dismissive of participant’s concerns or complaints. (2) DONE PARTIALLY = the facilitator is generally warm and friendly to patient, but does not demonstrate the ability to put him/herself in the experience of the participant. (3) DONE WELL = the facilitator demonstrates that he/she understands the experience of patient in a genuine and sincere manner. |
| 8.How well did the facilitator **collaboratively set goal and address participants' expectations**? | (0) NOT ASSESSED = this factor was not assessed because it was not applicable or because it did not emerge in the session. (1) NEEDS IMPROVEMENT = the facilitator does not ask participant about his/her goals and expectations for treatment, or the facilitator just tells the participant what to do without asking his/her expectations (2) DONE PARTIALLY= the facilitator asks participant about goals but does not discuss if these are realistic or can be accomplished. (3) DONE WELL = the facilitator asks about goals and discusses with participant what is and is not achievable through treatment, and the facilitator and participant collaboratively establish treatment plan |
| 9.How well did the facilitator **promote realistic hope for change**? | (0) NOT ASSESSED = this factor was not assessed because it was not applicable or because it did not emerge in the session. (1) NEEDS IMPROVEMENT = the facilitator either gives no hope (e.g., you will never get better) or gives unrealistic expectations (e.g., you will be cured in a few weeks and never have problems again) for what to expect in intervention. (2) DONE PARTIALLY= the facilitator vaguely tells the participant what will happen during intervention, e.g., missing out the key information such as the purpose and duration of the intervention. (3) DONE WELL = the facilitator helps the participant feel positive about the future and creates realistic expectations about what can and cannot be achieved through the intervention, and the facilitator checks the participant’s understanding of realistic change. |
| 10.How well did the facilitator give **psychoeducation and use local terminology**? | (0) NOT ASSESSED = this factor was not assessed because it was not applicable or because it did not emerge in the session. (1) NEEDS IMPROVEMENT = the facilitator uses technical terms to explain mental health or uses stigmatizing terms or does not explain how intervention works (2) DONE PARTIALLY= the facilitator uses a limited amount of technical terms and no stigmatizing terms, but the facilitator does not incorporate local concepts into psychoeducation  (3) DONE WELL = the facilitator conducts psychoeducation using local concepts, terms, and idioms of distress to explain mental health and intervention in non-stigmatizing language and checks to see if the participant understands. |
| 11.How well did the facilitator e**licitate feedback when providing advice, suggestions, and recommendations**? | (0) NOT ASSESSED = this factor was not assessed because it was not applicable or because it did not emerge in the session. (1) NEEDS IMPROVEMENT = the facilitator lectures a participant about what to do without asking if this is acceptable and comfortable for the participant. (2) DONE PARTIALLY= the facilitator gives focused advice but does not ask for feedback from participant to see if the advice is helpful. (3) DONE WELL = the facilitator gives a few suggestions when asked by participant and asks for participant's feedback about suggestions. |

Note. The adaptation has been made based on the original ENACT scale and its rating Kohrt,B.A, Ramaiya M.K, Rai S, Bhardwaj A., & Jordans M. D. Development of a scoring system for non-specialist ratings of clinical competence in global mental health: a qualitative process evaluation of the Enhancing Assessment of Common Therapeutic Factors (ENACT) scale. Global Mental Health, **2**, e23.). The "Not assessed" option was taken from a recent adaptation study in South Africa (Spedding, M., Kohrt, B., Myers, B., Stein, D. J., Petersen, I., Lund, C., & Sorsdahl, K. (2022). ENhancing Assessment of Common Therapeutic factors (ENACT) tool: adaptation and psychometric properties in South Africa. Global Mental Health, 9, 375-383.) Raters assessed each facilitators’ performance on a 4-point scale (0= *not assessed*, 3= *done well*) across 11 domains (e.g., verbal and non-verbal communication skills, explaining privacy, building rapport and self-disclosure, and normalising feelings). Scores ≥ 2 were considered to represent an acceptable level of competency.
