# Supplementary material for: Efficacy of a World Health Organization–Guided Self-Help Intervention for Reducing Psychological Distress in Afghan Refugees: Randomized Controlled Trial
Source: JMIR Ment Health. 2026 May 20;13:e89928. doi: 10.2196/89928 (PMC13189532; doi:10.2196/89928)
Supplement: Multimedia Appendix 3 [file mental-v13-e89928-s003.docx]

# **Table S1.** Distributions of lost to follow-up participants.

|  | DWM condition  (N, %) | RAC condition  (N, %) | | Total  (N, %) | Chi-square test (P-value) |
| --- | --- | --- | --- | --- | --- |
| Mid-treatment | 17 (8.4%) | | 7 (6.9%) | 24 (7.9%) | .204 (.652) |
| Post-treatment | 23 (11.4%) | | 13 (12.9%) | 36 (11.9%) | .142 (.706) |
| One-month follow-up | 26 (12.9%) | | 11 (10.9%) | 37 (12.2%) | .246 (.620) |
