# Supplementary material for: Efficacy of a World Health Organization–Guided Self-Help Intervention for Reducing Psychological Distress in Afghan Refugees: Randomized Controlled Trial
Source: JMIR Ment Health. 2026 May 20;13:e89928. doi: 10.2196/89928 (PMC13189532; doi:10.2196/89928)
Supplement: Multimedia Appendix 4 [file mental-v13-e89928-s004.docx]

# **Table S1.** Key characteristics of lost versus retained participants.

|  | Retained at follow-up (N=266) Mean(SD)/%(n) | Lost to follow-up (N=37) Mean(SD)/%(n) | Difference (SE) | SMD |
| --- | --- | --- | --- | --- |
| Age (years) | 30.94 (8.53) | 29.43 (8.66%) | -1.51 (1.52) | 0.18 |
| Gender |  |  |  |  |
| Female | 27.8% (74) | 18.9% (7) | 8.9 % (0.07) | 0.21 |
| Male | 72.2% (192) | 81.1% (30) | -8.9 (0.07) |  |
| Education level |  |  |  | 0.28 |
| No education or primary education | 57.9% (154) | 62.2% (23) | -4.3% (0.09) |  |
| High school or trade training | 32.7% (87) | 35.1% (13) | -2.4% (0.08) |  |
| Tertiary education | 9.4% (25) | 2.7% (1) | 6.7% (0.03) |  |
| Marital status |  |  |  | 0.14 |
| Married or in a relationship | 63.5% (169) | 70.3% (26) | -6.7% (0.08) |  |
| Not currently in a relationship | 36.5% (97) | 29.7% (11) | 6.7% (0.08) |  |
| Country of origin |  |  |  |  |
| Iran | 6.4% (17) | 13.5% (5) | -7.1% (0.06) | 0.28 |
| Afghanistan | 246 (92.5) | 86.5% (32) | 6% (0.06) |  |
| Pakistan | 1.1% (3) | 0% (0) | 1% (0.01) |  |
| City of residency |  |  |  | 0.11 |
| Bogor | 61.7% (164) | 64.9% (24) | -3.2% (0.08) |  |
| Jakarta | 23.3% (62) | 18.9% (7) | 4.4% (0.07) |  |
| Other | 15% (62) | 16.2% (16) | -1.2% (0.06) |  |
| Living situation |  |  |  | 0.11 |
| Independent housing | 88.7% (236) | 91.9% (34) | -3.2% (0.05) |  |
| Refugee shelter | 11.3% (30) | 8.1% (3) | 3.2% (0.05) |  |
| Financial status |  |  |  |  |
| Earning money | 21.6% (57) | 16.2% (6) | 5.4% (0.07) |  |
| Not earning money | 78.4% (207) | 83.8% (31) | -5.4% (0.07) |  |
| PTE Exposure | 2.73 (1.79) | 2.59 (2.01) | -0.14 (0.35) | 0.07 |
| Time in Indonesia (years) | 8.25 (2.43) | 8.86 (1.80) | 0.61 (0.33) | 0.29 |
| Family separation |  |  |  | 0.14 |
| No immediate family in Indonesia | 60.9% (162) | 54.1% (20) | 6.8% (0.09) |  |
| Some immediate family in Indonesia; or | 23.7% (63) | 27% (10) | -3.3% (0.08) |  |
| All immediate family in Indonesia | 15.4% (41) | 18.9 (7) | -3.5% (0.07) |  |
| Previous psychological treatment |  |  |  | 0.16 |
| No | 74.4% (198) | 81.1% (30) | -6.6% (0.07) |  |
| Yes | 25.6% (68) | 18.9 (7) | 6.6% (0.07) |  |
| Severity of daily stressors | 2.47 (0.69) | 2.38 (0.79) | -0.09 (0.14) | 0.12 |
| Psychological distress (K1) | 33.26 (6.19) | 32.27 (6.73) | -0.99 (1.17) | 0.15 |
| PTSD symptoms total score (PCL) | 13.88 (4.84) | 13.38 (4.41) | -0.51 (0.78) | 0.11 |
| Wellbeing total score (WHO-5) | 5.68 (3.91) | 7.38 (4.37) | 1.69 (0.76) | 0.41 |
| Disability total score (WHODAS) | 29.05 (9.28) | 29.22 (8.79) | 0.16 (1.55) | 0.02 |
| Social Functioning total score (SAS) | 25.17 (5.01) | 27.03 (5.61) | 1.85 (0.97) | 0.35 |

Note. SMD: Standardized mean difference.
