# Supplementary material for: Efficacy of a World Health Organization–Guided Self-Help Intervention for Reducing Psychological Distress in Afghan Refugees: Randomized Controlled Trial
Source: JMIR Ment Health. 2026 May 20;13:e89928. doi: 10.2196/89928 (PMC13189532; doi:10.2196/89928)
Supplement: Multimedia Appendix 5 [file mental-v13-e89928-s005.docx]

# **Table S1.** Summary results for per-protocol analyses.

|  | DWM Estimated Average Value (SE) | RAC Estimated Average Value (SE) | b (SE) | P-value | βeta (SE) |
| --- | --- | --- | --- | --- | --- |
| K10 score |  |  |  |  |  |
| Mid-treatment | 29.085 (0.444) | 32.046 (0.517) | -2.961 (0.687) | <.001 | -0.478 (0.111) |
| Post-treatment | 27.501 (0.482) | 31.112 (0.601) | -3.611 (0.776) | <.001 | -0.583 (0.125) |
| Follow-up | 26.396 (0.529) | 29.273 (0.690) | -2.877 (0.876) | .001 | -0.465 (0.142) |
| PCL score |  |  |  |  |  |
| Mid-treatment | 11.536 (0.305) | 13.936 (0.392) | -2.400 (0.504) | <.001 | -0.499 (0.105) |
| Post-treatment | 11.446 (0.347) | 13.780 (0.421) | -2.334 (0.552) | <.001 | -0.485 (0.115) |
| Follow-up | 10.862 (0.373) | 12.842 (0.466) | -1.981 (0.602) | .001 | -0.412 (0.125) |
| WHO-5 score | |  |  |  |  |
| Mid-treatment | 8.360 (0.345) | 6.840 (0.377) | 1.519 (0.514) | .004 | 0.384 (0.130) |
| Post-treatment | 9.039 (0.386) | 6.589 (0.420) | 2.450 (0.577) | <.001 | 0.619 (0.146) |
| Follow-up | 9.236 (0.395) | 7.875 (0.454) | 1.361 (0.609) | .027 | 0.344 (0.154) |
| WHODAS Score | |  |  |  |  |
| Mid-treatment | 25.999 (0.579) | 28.069 (0.755) | -2.069 (0.962) | .033 | -0.223 (0.104) |
| Post-treatment | 25.246 (0.584) | 27.443 (0.857) | -2.198 (1.046) | .037 | -0.237 (0.113) |
| Follow-up | 23.636 (0.649) | 25.808 (0.735) | -2.172 (0.993) | .030 | -0.234 (0.107) |
| SAS Score | |  |  |  |  |
| Mid-treatment | 30.484 (0.362) | 28.443 (0.457) | 2.041 (0.594) | .001 | 0.403 (0.117) |
| Post-treatment | 30.673 (0.339) | 28.862 (0.490) | 1.811 (0.608) | .003 | 0.358 (0.120) |
| Follow-up | 31.150 (0.356) | 29.817 (0.481) | 1.333 (0.607) | .029 | 0.263 (0.120) |
| PSYCHLOPS | |  |  |  |  |
| Mid-treatment | 8.721 (0.128) | 9.004 (0.130) | -0.283 (0.183) | .123 | -0.229 (0.148) |
| Post-treatment | 8.687 (0.119) | 9.191 (0.126) | -0.504 (0.174) | .004 | -0.407 (0.141) |
| Follow-up | 8.551 (0.145) | 8.971 (0.128) | -0.420 (0.194) | .031 | -0.339 (0.157) |
