# Supplementary material for: Efficacy of a World Health Organization–Guided Self-Help Intervention for Reducing Psychological Distress in Afghan Refugees: Randomized Controlled Trial
Source: JMIR Ment Health. 2026 May 20;13:e89928. doi: 10.2196/89928 (PMC13189532; doi:10.2196/89928)
Supplement: Multimedia Appendix 6 [file mental-v13-e89928-s006.docx]

# **Table S1.** Summary results for completers-only analyses.

|  | DWM Estimated Average Value (SE) | RAC Estimated Average Value (SE) | b (SE) | P-value | βeta (SE) |
| --- | --- | --- | --- | --- | --- |
| K10 score |  |  |  |  |  |
| Mid-treatment | 29.275 (0.466) | 31.907 (0.527) | -2.633 (0.710) | .001 | -0.425 (0.115) |
| Post-treatment | 27.639 (0.498) | 30.998 (0.601) | -3.359 (0.787) | <.001 | -0.543 (0.127) |
| Follow-up | 26.516 (0.534) | 29.218 (0.691) | -2.702 (0.881) | .003 | -0.437 (0.142) |
| PCL score |  |  |  |  |  |
| Mid-treatment | 11.713 (0.329) | 13.939 (0.401) | 13.939 (0.401) | <.001 | -0.460 (0.109) |
| Post-treatment | 11.570 (0.360) | 13.705 (0.418) | 13.705 (0.418) | .001 | -0.441 (0.115) |
| Follow-up | 10.922 (0.378) | 12.828 (0.466) | 12.828 (0.466) | .002 | -0.393 (0.125) |
| WHO-5 score | |  |  |  |  |
| Mid-treatment | 8.293 (0.357) | 6.833 (0.389) | 1.460 (0.533) | .007 | 0.374 (0.136) |
| Post-treatment | 8.928 (0.396) | 6.448 (0.404) | 2.480 (0.574) | <.001 | 0.635 (0.147) |
| Follow-up | 9.135 (0.392) | 7.833 (0.451) | 1.303 (0.606) | .033 | 0.333 (0.155) |
| WHODAS Score | |  |  |  |  |
| Mid-treatment | 26.349 (0.625) | 28.121 (0.786) | -1.772 (1.013) | .082 | 0.376 (0.122) |
| Post-treatment | 25.436 (0.610) | 27.268 (0.852) | -1.832 (1.056) | .085 | 0.323 (0.123) |
| Follow-up | 23.857 (0.668) | 25.765 (0.741) | -1.908 (1.009) | .060 | 0.255 (0.121) |
| SAS Score | |  |  |  |  |
| Mid-treatment | 30.434 (0.375) | 28.547 (0.468) | 1.886 (0.611) | .002 | -0.191 (0.109) |
| Post-treatment | 30.562 (0.349) | 28.945 (0.492) | 1.617 (0.617) | .010 | -0.197 (0.114) |
| Follow-up | 31.125 (0.353) | 29.847 (0.483) | 1.278 (0.608) | .037 | -0.206 (0.109) |
| PSYCHLOPS | |  |  |  |  |
| Mid-treatment | 8.771 (0.130) | 8.989 (0.135) | -0.218 (0.188) | .247 | -0.181 (0.149) |
| Post-treatment | 8.730 (0.121) | 9.185 (0.128) | -0.455 (0.177) | .010 | -0.361 (0.140) |
| Follow-up | 8.584 (0.144) | 8.967 (0.128) | -0.383 (0.194) | .049 | -0.304 (0.154) |
