# Supplementary material for: Efficacy of a World Health Organization–Guided Self-Help Intervention for Reducing Psychological Distress in Afghan Refugees: Randomized Controlled Trial
Source: JMIR Ment Health. 2026 May 20;13:e89928. doi: 10.2196/89928 (PMC13189532; doi:10.2196/89928)
Supplement: Checklist 1 [file mental-v13-e89928-s007.pdf]

# CONSORT-EHEALTH (V 1.6.1) - Submission/Publication Form

The CONSORT-EHEALTH checklist is intended for authors of randomized trials evaluating web-based and Internet-based applications/interventions, including mobile interventions, electronic games (incl multiplayer games), social media, certain telehealth applications, and other interactive and/or networked electronic applications. Some of the items (e.g. all subitems under item 5 - description of the intervention) may also be applicable for other study designs.

The goal of the CONSORT EHEALTH checklist and guideline is to be
a) a guide for reporting for authors of RCTs,
b) to form a basis for appraisal of an ehealth trial (in terms of validity)

CONSORT-EHEALTH items/subitems are MANDATORY reporting items for studies published in the Journal of Medical Internet Research and other journals / scientific societies endorsing the checklist.

Items numbered 1., 2., 3., 4a., 4b etc are original CONSORT or CONSORT-NPT (non-pharmacologic treatment) items.
Items with Roman numerals (i., ii, iii, iv etc.) are CONSORT-EHEALTH extensions/clarifications.

As the CONSORT-EHEALTH checklist is still considered in a formative stage, we would ask that you also RATE ON A SCALE OF 1-5 how important/useful you feel each item is FOR THE PURPOSE OF THE CHECKLIST and reporting guideline (optional).

Mandatory reporting items are marked with a red *.
In the textboxes, either copy & paste the relevant sections from your manuscript into this form - please include any quotes from your manuscript in QUOTATION MARKS,
or answer directly by providing additional information not in the manuscript, or elaborating on why the item was not relevant for this study.

YOUR ANSWERS WILL BE PUBLISHED AS A SUPPLEMENTARY FILE TO YOUR PUBLICATION IN JMIR AND ARE CONSIDERED PART OF YOUR PUBLICATION (IF ACCEPTED).
Please fill in these questions diligently. Information will not be copyedited, so please use proper spelling and grammar, use correct capitalization, and avoid abbreviations.

DO NOT FORGET TO SAVE AS PDF _AND_ CLICK THE SUBMIT BUTTON SO YOUR ANSWERS ARE IN OUR DATABASE !!!

Citation Suggestion (if you append the pdf as Appendix we suggest to cite this paper in the caption):
Eysenbach G, CONSORT-EHEALTH Group
CONSORT-EHEALTH: Improving and Standardizing Evaluation Reports of Web-based and Mobile Health Interventions
J Med Internet Res 2011;13(4):e126

URL: http://www.jmir.org/2011/4/e126/
doi: 10.2196/jmir.1923
PMID: 22209829

**gulsahkurttt@gmail.com** Hesap değiştir

Paylaşılmıyor

Taslak kaydedildi

* Zorunlu soruyu belirtir

Your name *

First Last

Angela Nickerson

Primary Affiliation (short), City, Country *

University of Toronto, Toronto, Canada

School of Psychology

Your e-mail address *

abc@gmail.com

anickerson@psy.unsw.edu.au

Title of your manuscript *

Provide the (draft) title of your manuscript.

The efficacy of a WHO guided self-help intervention for reducing psychological distress in Afghan refugees: A randomized controlled trial

Name of your App/Software/Intervention *

If there is a short and a long/alternate name, write the short name first and add the long name in brackets.

Doing What Matters in Times of Stress (DWM)

Evaluated Version (if any)

e.g. "V1", "Release 2017-03-01", "Version 2.0.27913"

Yanıtınız

Language(s) *

What language is the intervention/app in? If multiple languages are available, separate by comma (e.g. "English, French")

Farsi

URL of your Intervention Website or App

e.g. a direct link to the mobile app on app in appstore (itunes, Google Play), or URL of the website. If the intervention is a DVD or hardware, you can also link to an Amazon page.

https://www.who.int/publications-detail-redirect/9789240003927

URL of an image/screenshot (optional)

Yanıtınız

Accessibility *

Can an enduser access the intervention presently?

- (selected) access is free and open
- access only for special usergroups, not open
- access is open to everyone, but requires payment/subscription/in-app purchases
- app/intervention no longer accessible
- Diğer:

Primary Medical Indication/Disease/Condition *

e.g. "Stress", "Diabetes", or define the target group in brackets after the condition, e.g. "Autism (Parents of children with)", "Alzheimers (Informal Caregivers of)"

Psychological stress (Refugees)

Primary Outcomes measured in trial *

comma-separated list of primary outcomes reported in the trial

psychological distress at post-treatment

Secondary/other outcomes

Are there any other outcomes the intervention is expected to affect?

PTSD symptoms, personally identified psychological problems, wellbeing, and functioning at post-treatment and one-month follow-up assessment and psychological distress at one-month follow-up assessment

Recommended "Dose" *

What do the instructions for users say on how often the app should be used?

- ( ) Approximately Daily
- (x) Approximately Weekly
- ( ) Approximately Monthly
- ( ) Approximately Yearly
- ( ) "as needed"
- ( ) Diğer:

Approx. Percentage of Users (starters) still using the app as recommended after 3 months *

- (x) unknown / not evaluated
- ( ) 0-10%
- ( ) 11-20%
- ( ) 21-30%
- ( ) 31-40%
- ( ) 41-50%
- ( ) 51-60%
- ( ) 61-70%
- ( ) 71%-80%
- ( ) 81-90%
- ( ) 91-100%
- ( ) Diğer:

Overall, was the app/intervention effective? *

- (selected) yes: all primary outcomes were significantly better in intervention group vs control
- partly: SOME primary outcomes were significantly better in intervention group vs control
- no statistically significant difference between control and intervention
- potentially harmful: control was significantly better than intervention in one or more outcomes
- inconclusive: more research is needed
- Diğer:

Article Preparation Status/Stage *

At which stage in your article preparation are you currently (at the time you fill in this form)

- not submitted yet - in early draft status
- not submitted yet - in late draft status, just before submission
- submitted to a journal but not reviewed yet
- submitted to a journal and after receiving initial reviewer comments
- (selected) submitted to a journal and accepted, but not published yet
- published
- Diğer:

Journal *

If you already know where you will submit this paper (or if it is already submitted), please provide the journal name (if it is not JMIR, provide the journal name under "other")

- ○ not submitted yet / unclear where I will submit this
- ○ Journal of Medical Internet Research (JMIR)
- ○ JMIR mHealth and UHealth
- ○ JMIR Serious Games
- ◉ JMIR Mental Health
- ○ JMIR Public Health
- ○ JMIR Formative Research
- ○ Other JMIR sister journal
- ○ Diğer:

Is this a full powered effectiveness trial or a pilot/feasibility trial? *

- ○ Pilot/feasibility
- ◉ Fully powered

Manuscript tracking number *

If this is a JMIR submission, please provide the manuscript tracking number under "other" (The ms tracking number can be found in the submission acknowledgement email, or when you login as author in JMIR. If the paper is already published in JMIR, then the ms tracking number is the four-digit number at the end of the DOI, to be found at the bottom of each published article in JMIR)

- ○ no ms number (yet) / not (yet) submitted to / published in JMIR
- ◉ Diğer: 89928

TITLE AND ABSTRACT

1a) TITLE: Identification as a randomized trial in the title

1a) Does your paper address CONSORT item 1a? *

I.e does the title contain the phrase "Randomized Controlled Trial"? (if not, explain the reason under "other")

- (selected) yes
- Diğer:

1a-i) Identify the mode of delivery in the title

Identify the mode of delivery. Preferably use “web-based” and/or “mobile” and/or “electronic game” in the title. Avoid ambiguous terms like “online”, “virtual”, “interactive”. Use “Internet-based” only if Intervention includes non-web-based Internet components (e.g. email), use “computer-based” or “electronic” only if offline products are used. Use “virtual” only in the context of “virtual reality” (3-D worlds). Use “online” only in the context of “online support groups”. Complement or substitute product names with broader terms for the class of products (such as “mobile” or “smart phone” instead of “iphone”), especially if the application runs on different platforms.

| | 1 | 2 | 3 | 4 | 5 | |
|---|---|---|---|---|---|---|
| subitem not at all important | ○ | ○ | ○ | ○ | ○ | essential |

Does your paper address subitem 1a-i? *

Copy and paste relevant sections from manuscript title (include quotes in quotation marks "like this" to indicate direct quotes from your manuscript), or elaborate on this item by providing additional information not in the ms, or briefly explain why the item is not applicable/relevant for your study

"WHO guided self-help intervention"

1a-ii) Non-web-based components or important co-interventions in title

Mention non-web-based components or important co-interventions in title, if any (e.g., “with telephone support”).

| | 1 | 2 | 3 | 4 | 5 | |
|---|---|---|---|---|---|---|
| subitem not at all important | ○ | ○ | ○ | ○ | ○ | essential |

Does your paper address subitem 1a-ii?

Copy and paste relevant sections from manuscript title (include quotes in quotation marks "like this" to indicate direct quotes from your manuscript), or elaborate on this item by providing additional information not in the ms, or briefly explain why the item is not applicable/relevant for your study

Yanıtınız

1a-iii) Primary condition or target group in the title

Mention primary condition or target group in the title, if any (e.g., “for children with Type I Diabetes”) Example: A Web-based and Mobile Intervention with Telephone Support for Children with Type I Diabetes: Randomized Controlled Trial

| | 1 | 2 | 3 | 4 | 5 | |
|---|---|---|---|---|---|---|
| subitem not at all important | ○ | ○ | ○ | ○ | ○ | essential |

Does your paper address subitem 1a-iii? *

Copy and paste relevant sections from manuscript title (include quotes in quotation marks "like this" to indicate direct quotes from your manuscript), or elaborate on this item by providing additional information not in the ms, or briefly explain why the item is not applicable/relevant for your study

"a WHO guided self-help intervention for reducing psychological distress in Afghan refugees"

## 1b) ABSTRACT: Structured summary of trial design, methods, results, and conclusions

NPT extension: Description of experimental treatment, comparator, care providers, centers, and blinding status.

## 1b-i) Key features/functionalities/components of the intervention and comparator in the METHODS section of the ABSTRACT

Mention key features/functionalities/components of the intervention and comparator in the abstract. If possible, also mention theories and principles used for designing the site. Keep in mind the needs of systematic reviewers and indexers by including important synonyms. (Note: Only report in the abstract what the main paper is reporting. If this information is missing from the main body of text, consider adding it)

| | 1 | 2 | 3 | 4 | 5 | |
|---|---|---|---|---|---|---|
| subitem not at all important | ○ | ○ | ○ | ○ | ○ | essential |

## Does your paper address subitem 1b-i? *

Copy and paste relevant sections from the manuscript abstract (include quotes in quotation marks "like this" to indicate direct quotes from your manuscript), or elaborate on this item by providing additional information not in the ms, or briefly explain why the item is not applicable/relevant for your study

"A single-blind randomized controlled trial with 303 Farsi-speaking refugees was conducted between June 2024 and June 2025. Participants with moderate to high psychological distress (Kessler Psychological Distress Scale, K10 ≥ 20) were randomly allocated to the facilitator-guided individual DWM condition (N = 202) or a repeated assessment control (RAC) condition (N = 101)"

1b-ii) Level of human involvement in the METHODS section of the ABSTRACT

Clarify the level of human involvement in the abstract, e.g., use phrases like “fully automated” vs. “therapist/nurse/care provider/physician-assisted” (mention number and expertise of providers involved, if any). (Note: Only report in the abstract what the main paper is reporting. If this information is missing from the main body of text, consider adding it)

| | 1 | 2 | 3 | 4 | 5 | |
|---|---|---|---|---|---|---|
| subitem not at all important | ○ | ○ | ○ | ○ | ○ | essential |

Does your paper address subitem 1b-ii?

Copy and paste relevant sections from the manuscript abstract (include quotes in quotation marks "like this" to indicate direct quotes from your manuscript), or elaborate on this item by providing additional information not in the ms, or briefly explain why the item is not applicable/relevant for your study

"the facilitator-guided individual DWM condition"

1b-iii) Open vs. closed, web-based (self-assessment) vs. face-to-face assessments in the METHODS section of the ABSTRACT

Mention how participants were recruited (online vs. offline), e.g., from an open access website or from a clinic or a closed online user group (closed usergroup trial), and clarify if this was a purely web-based trial, or there were face-to-face components (as part of the intervention or for assessment). Clearly say if outcomes were self-assessed through questionnaires (as common in web-based trials). Note: In traditional offline trials, an open trial (open-label trial) is a type of clinical trial in which both the researchers and participants know which treatment is being administered. To avoid confusion, use “blinded” or “unblinded” to indicated the level of blinding instead of “open”, as “open” in web-based trials usually refers to “open access” (i.e. participants can self-enrol). (Note: Only report in the abstract what the main paper is reporting. If this information is missing from the main body of text, consider adding it)

| | 1 | 2 | 3 | 4 | 5 | |
|---|---|---|---|---|---|---|
| subitem not at all important | ○ | ○ | ○ | ○ | ○ | essential |

Does your paper address subitem 1b-iii?

Copy and paste relevant sections from the manuscript abstract (include quotes in quotation marks "like this" to indicate direct quotes from your manuscript), or elaborate on this item by providing additional information not in the ms, or briefly explain why the item is not applicable/relevant for your study

Yanıtınız

1b-iv) RESULTS section in abstract must contain use data

Report number of participants enrolled/assessed in each group, the use/uptake of the intervention (e.g., attrition/adherence metrics, use over time, number of logins etc.), in addition to primary/secondary outcomes. (Note: Only report in the abstract what the main paper is reporting. If this information is missing from the main body of text, consider adding it)

| | 1 | 2 | 3 | 4 | 5 | |
|---|---|---|---|---|---|---|
| subitem not at all important | ○ | ○ | ○ | ○ | ○ | essential |

Does your paper address subitem 1b-iv?

Copy and paste relevant sections from the manuscript abstract (include quotes in quotation marks "like this" to indicate direct quotes from your manuscript), or elaborate on this item by providing additional information not in the ms, or briefly explain why the item is not applicable/relevant for your study

Yanıtınız

1b-v) CONCLUSIONS/DISCUSSION in abstract for negative trials

Conclusions/Discussions in abstract for negative trials: Discuss the primary outcome - if the trial is negative (primary outcome not changed), and the intervention was not used, discuss whether negative results are attributable to lack of uptake and discuss reasons. (Note: Only report in the abstract what the main paper is reporting. If this information is missing from the main body of text, consider adding it)

| | 1 | 2 | 3 | 4 | 5 | |
|---|---|---|---|---|---|---|
| subitem not at all important | ○ | ○ | ○ | ○ | ○ | essential |

Does your paper address subitem 1b-v?

Copy and paste relevant sections from the manuscript abstract (include quotes in quotation marks "like this"  to indicate direct quotes from your manuscript), or elaborate on this item by providing additional information not in the ms, or briefly explain why the item is not applicable/relevant for your study

Yanıtınız

INTRODUCTION

2a) In INTRODUCTION: Scientific background and explanation of rationale

2a-i) Problem and the type of system/solution

Describe the problem and the type of system/solution that is object of the study: intended as stand-alone intervention vs. incorporated in broader health care program? Intended for a particular patient population? Goals of the intervention, e.g., being more cost-effective to other interventions, replace or complement other solutions? (Note: Details about the intervention are provided in “Methods” under 5)

| | 1 | 2 | 3 | 4 | 5 | |
|---|---|---|---|---|---|---|
| subitem not at all important | ○ | ○ | ○ | ○ | ○ | essential |

Does your paper address subitem 2a-i? *

Copy and paste relevant sections from the manuscript (include quotes in quotation marks "like this" to indicate direct quotes from your manuscript), or elaborate on this item by providing additional information not in the ms, or briefly explain why the item is not applicable/relevant for your study

"The objective of this study was to evaluate the efficacy of DWM in reducing psychological distress (primary outcome), PTSD symptoms, personally identified psychological problems improving wellbeing and functioning amongst Afghan refugees living in Indonesia, as well as to evaluate the clinical significance of associated symptom change."

2a-ii) Scientific background, rationale: What is known about the (type of) system

Scientific background, rationale: What is known about the (type of) system that is the object of the study (be sure to discuss the use of similar systems for other conditions/diagnoses, if appropiate), motivation for the study, i.e. what are the reasons for and what is the context for this specific study, from which stakeholder viewpoint is the study performed, potential impact of findings [2]. Briefly justify the choice of the comparator.

| | 1 | 2 | 3 | 4 | 5 | |
|---|---|---|---|---|---|---|
| subitem not at all important | ○ | ○ | ○ | ○ | ○ | essential |

Does your paper address subitem 2a-ii? *

Copy and paste relevant sections from the manuscript (include quotes in quotation marks "like this" to indicate direct quotes from your manuscript), or elaborate on this item by providing additional information not in the ms, or briefly explain why the item is not applicable/relevant for your study

"There is a need for low-cost, scalable, and sustainable approaches to reduce psychological symptoms amongst refugees in LMICs. Interventions that require mental health specialists, intensive training and supervision, and face-to-face delivery over several months may not be feasible in low-resource settings[7]. To address this problem, the World Health Organization (WHO) has developed a suite of scalable interventions to alleviate mental burden in humanitarian and low-resource settings[8,9]. One of these – Doing What Matters in Times of Stress[10] (DWM) –is a predominantly self-help intervention, where individuals are provided with a workbook and audio files in their native language, and receive short phone calls from non-specialists to support their engagement with the materials. Although some trials have been conducted with DWM in combination with other interventions[11], the only study to date testing DWM as a standalone intervention was undertaken with Syrian refugees and Turkish nationals in Türkiye[12]. While this trial found promising evidence for reduction in PTSD symptoms in Syrian refugees and depression symptoms in Turkish nationals, it was underpowered to detect group differences. Other studies have tested Self Help-Plus, DWM's group-based alternative, demonstrating its efficacy in preventing mental disorders amongst refugees with sub-clinical symptoms in Western Europe and Türkiye[13,14], and reducing psychological distress in female Sudanese refugees in a camp setting in Uganda[15]. To date, however, there has not been a fully-powered trial investigating the efficacy of the DWM program in reducing psychological distress among refugees living in a LMIC. This is an important gap as there is evidence that individually-supported interventions may confer greater benefit than their group-based counterparts[16]. Further, self-help approaches with individual guidance may overcome important implementation barriers for refugees in transit settings, including stigma, logistical challenges, and the need for individual tailoring of content and delivery[17].

In this study, we conducted a randomized controlled trial evaluating the efficacy of DWM in reducing psychological distress amongst Afghan refugees living in prolonged displacement in the greater Jakarta area in Indonesia. Indonesia is key transit country for refugees in the Asia-Pacific, hosting approximately 12,000 refugees[18]. Like many transit countries, Indonesia is a non-signatory to the United Nations Refugee and its Protocol and does not permanently resettle refugees. In this context (as in many other transit settings), refugees are exposed to a myriad of daily stressors and have access to few formal services [19]. This means that the conditions of safety and security that are important for mental health recovery following adversity are notably absent[20], and that it is incumbent on refugee communities and refugee-led organizations to provide necessary supports to displaced individuals. There is thus an urgent need to evaluate the efficacy of scalable self-help approaches that can be implemented by refugee communities to reduce psychological distress."

2b) In INTRODUCTION: Specific objectives or hypotheses

Does your paper address CONSORT subitem 2b? *

Copy and paste relevant sections from the manuscript (include quotes in quotation marks "like this" to indicate direct quotes from your manuscript), or elaborate on this item by providing additional information not in the ms, or briefly explain why the item is not applicable/relevant for your study

"The objective of this study was to evaluate the efficacy of DWM in reducing psychological distress (primary outcome), PTSD symptoms, personally identified psychological problems improving wellbeing and functioning amongst Afghan refugees living in Indonesia, as well as to evaluate the clinical significance of associated symptom change. We hypothesized that, compared to a repeated assessment control group (RAC), refugees who received DWM would show greater improvements in psychological distress at post-treatment (primary outcome) and one-month follow-up, as well as greater improvements in PTSD symptoms, functional impairment, personally identified psychological problems, social functioning and wellbeing."

METHODS

3a) Description of trial design (such as parallel, factorial) including allocation ratio

Does your paper address CONSORT subitem 3a? *

Copy and paste relevant sections from the manuscript (include quotes in quotation marks "like this" to indicate direct quotes from your manuscript), or elaborate on this item by providing additional information not in the ms, or briefly explain why the item is not applicable/relevant for your study

"We conducted a two-arm single-blind randomized controlled trial with refugees from Afghanistan living in Indonesia." "Eligible participants were randomly assigned to DWM or the RAC condition at a 2:1 ratio. This ratio was implemented to allow for the investigation of mechanisms of action within the DWM condition (which will be detailed in future reports). Randomization was conducted by a researcher who was not involved in the delivery of the intervention. Randomization was performed using a computerized software (REDCap)30. Assessors were masked to treatment condition allocation. Assessors were managed separately from other members of the research team and did not interact with the DWM facilitators."

3b) Important changes to methods after trial commencement (such as eligibility criteria), with reasons

Does your paper address CONSORT subitem 3b? *

Copy and paste relevant sections from the manuscript (include quotes in quotation marks "like this" to indicate direct quotes from your manuscript), or elaborate on this item by providing additional information not in the ms, or briefly explain why the item is not applicable/relevant for your study

No changes have been made after trial commencement.

3b-i) Bug fixes, Downtimes, Content Changes

Bug fixes, Downtimes, Content Changes: ehealth systems are often dynamic systems. A description of changes to methods therefore also includes important changes made on the intervention or comparator during the trial (e.g., major bug fixes or changes in the functionality or content) (5-iii) and other “unexpected events” that may have influenced study design such as staff changes, system failures/downtimes, etc. [2].

| | 1 | 2 | 3 | 4 | 5 | |
|---|---|---|---|---|---|---|
| subitem not at all important | ○ | ○ | ○ | ○ | ○ | essential |

Does your paper address subitem 3b-i?

Copy and paste relevant sections from the manuscript (include quotes in quotation marks "like this" to indicate direct quotes from your manuscript), or elaborate on this item by providing additional information not in the ms, or briefly explain why the item is not applicable/relevant for your study

Yanıtınız

4a) Eligibility criteria for participants

Does your paper address CONSORT subitem 4a? *

Copy and paste relevant sections from the manuscript (include quotes in quotation marks "like this" to indicate direct quotes from your manuscript), or elaborate on this item by providing additional information not in the ms, or briefly explain why the item is not applicable/relevant for your study

"The inclusion criteria were: (a) 18 years or older, (b) from a refugee background (i.e., living in Indonesia and having registered with the UNHCR or intending to register with the UNHCR), (c) residing in Greater Jakarta area (Jakarta, Bogor, Depok, Tangerang, or Bekasi), (d) ability to speak and read Farsi, (e) elevated psychological distress (≥ 20 on K10 [29]), and (f) providing written/verbal informed consent before entering the study. The Farsi language was selected for this study as it represented a common language for Afghan refugees from Dari, Hazaragi, and Pashto-speaking backgrounds. Exclusion criteria included: (a) acute medical condition, (b) imminent suicide risk or expressed acute needs/protection risk (i.e., being at acute risk of domestic and/or sexual violence), (c) severe mental disorder (psychotic disorder or substance dependence), (d) severe cognitive impairment (e.g. severe intellectual disability or dementia), (e) concurrent psychological treatment (e.g. concurrently receiving psychological services from a psychologist, counsellor or mental health professional), (f) no access to smartphone or internet connection, and (g) having another household member who had already applied to take part in the study. Those excluded based on suicide risk, severe mental disorder, or severe cognitive impairment were referred to the available general or specialist services. "

4a-i) Computer / Internet literacy

Computer / Internet literacy is often an implicit “de facto” eligibility criterion - this should be explicitly clarified.

| | 1 | 2 | 3 | 4 | 5 | |
|---|---|---|---|---|---|---|
| subitem not at all important | ○ | ○ | ○ | ○ | ○ | essential |

Does your paper address subitem 4a-i?

Copy and paste relevant sections from the manuscript (include quotes in quotation marks "like this" to indicate direct quotes from your manuscript), or elaborate on this item by providing additional information not in the ms, or briefly explain why the item is not applicable/relevant for your study

Yanıtınız

4a-ii) Open vs. closed, web-based vs. face-to-face assessments:

Open vs. closed, web-based vs. face-to-face assessments: Mention how participants were recruited (online vs. offline), e.g., from an open access website or from a clinic, and clarify if this was a purely web-based trial, or there were face-to-face components (as part of the intervention or for assessment), i.e., to what degree got the study team to know the participant. In online-only trials, clarify if participants were quasi-anonymous and whether having multiple identities was possible or whether technical or logistical measures (e.g., cookies, email confirmation, phone calls) were used to detect/prevent these.

| | 1 | 2 | 3 | 4 | 5 | |
|---|---|---|---|---|---|---|
| subitem not at all important | ○ | ○ | ○ | ○ | ○ | essential |

Does your paper address subitem 4a-ii? *

Copy and paste relevant sections from the manuscript (include quotes in quotation marks "like this" to indicate direct quotes from your manuscript), or elaborate on this item by providing additional information not in the ms, or briefly explain why the item is not applicable/relevant for your study

"We elected to undertake this study in a digital format (i.e., recruitment via social media, assessment and delivery of DWM materials via Zoom calls) to increase access to the intervention in a population that was geographically dispersed around the greater Jakarta area. This methodology was considered feasible given (1) widespread internet access in Indonesia 27, (2) the successful implementation of digital procedures in our previous longitudinal study of 1,300 refugees living in Indonesia [19], and (3) reports from our study partners that smartphone usage was ubiquitous amongst refugees in Indonesia, consistent with evidence that refugees commonly use digital methods to stay in contact with family in the country of origin or displaced in other settings [27,28]. In recognition of the cost of accessing reliable internet in Indonesia, participants in both conditions were provided with an internet allowance." "Participants were recruited online through social media, advertisements on the study website, and flyers distributed to organizations supporting refugees in the Greater Jakarta area in Indonesia (Jakarta, Bogor, Depok, Tangerang, and Bekasi). Interested participants completed an online registration form that included study information, consent to a screening call with a trained assessor, the Kessler Psychological Distress Scale (K10)[29], an initial eligibility question confirming refugee status in Indonesia, and demographic questions. Those who consented and met the initial eligibility criteria were contacted by a trained assessor for an online screening call. During this call, information was collected on access to a digital device and internet connection, current treatment, suicide risk, and severe cognitive impairment. The K10 was also re-administered to verify self-reported responses. Participants who met the full eligibility criteria proceeded to the baseline assessment prior to randomization. All assessments were conducted online by trained assessors."

4a-iii) Information giving during recruitment

Information given during recruitment. Specify how participants were briefed for recruitment and in the informed consent procedures (e.g., publish the informed consent documentation as appendix, see also item X26), as this information may have an effect on user self-selection, user expectation and may also bias results.

| | 1 | 2 | 3 | 4 | 5 | |
|---|---|---|---|---|---|---|
| subitem not at all important | ○ | ○ | ○ | ○ | ○ | essential |

Does your paper address subitem 4a-iii?

Copy and paste relevant sections from the manuscript (include quotes in quotation marks "like this" to indicate direct quotes from your manuscript), or elaborate on this item by providing additional information not in the ms, or briefly explain why the item is not applicable/relevant for your study

Yanıtınız

4b) Settings and locations where the data were collected

Does your paper address CONSORT subitem 4b? *

Copy and paste relevant sections from the manuscript (include quotes in quotation marks "like this" to indicate direct quotes from your manuscript), or elaborate on this item by providing additional information not in the ms, or briefly explain why the item is not applicable/relevant for your study

"Participants were recruited online through social media, advertisements on the study website, and flyers distributed to organizations supporting refugees in the Greater Jakarta area in Indonesia (Jakarta, Bogor, Depok, Tangerang, and Bekasi). Interested participants completed an online registration form that included study information, consent to a screening call with a trained assessor, the Kessler Psychological Distress Scale (K10)[29], an initial eligibility question confirming refugee status in Indonesia, and demographic questions. Those who consented and met the initial eligibility criteria were contacted by a trained assessor for an online screening call. During this call, information was collected on access to a digital device and internet connection, current treatment, suicide risk, and severe cognitive impairment. The K10 was also re-administered to verify self-reported responses. Participants who met the full eligibility criteria proceeded to the baseline assessment prior to randomization. "

4b-i) Report if outcomes were (self-)assessed through online questionnaires

Clearly report if outcomes were (self-)assessed through online questionnaires (as common in web-based trials) or otherwise.

| | 1 | 2 | 3 | 4 | 5 | |
|---|---|---|---|---|---|---|
| subitem not at all important | ○ | ○ | ○ | ○ | ○ | essential |

Does your paper address subitem 4b-i? *

Copy and paste relevant sections from the manuscript (include quotes in quotation marks "like this" to indicate direct quotes from your manuscript), or elaborate on this item by providing additional information not in the ms, or briefly explain why the item is not applicable/relevant for your study

"Participants were recruited online through social media, advertisements on the study website, and flyers distributed to organizations supporting refugees in the Greater Jakarta area in Indonesia (Jakarta, Bogor, Depok, Tangerang, and Bekasi). Interested participants completed an online registration form that included study information, consent to a screening call with a trained assessor, the Kessler Psychological Distress Scale (K10)[29], an initial eligibility question confirming refugee status in Indonesia, and demographic questions. Those who consented and met the initial eligibility criteria were contacted by a trained assessor for an online screening call. During this call, information was collected on access to a digital device and internet connection, current treatment, suicide risk, and severe cognitive impairment. The K10 was also re-administered to verify self-reported responses. Participants who met the full eligibility criteria proceeded to the baseline assessment prior to randomization. All assessments were conducted online by trained assessors. "

4b-ii) Report how institutional affiliations are displayed

Report how institutional affiliations are displayed to potential participants [on ehealth media], as affiliations with prestigious hospitals or universities may affect volunteer rates, use, and reactions with regards to an intervention.(Not a required item – describe only if this may bias results)

| | 1 | 2 | 3 | 4 | 5 | |
|---|---|---|---|---|---|---|
| subitem not at all important | ○ | ○ | ○ | ○ | ○ | essential |

Does your paper address subitem 4b-ii?

Copy and paste relevant sections from the manuscript (include quotes in quotation marks "like this" to indicate direct quotes from your manuscript), or elaborate on this item by providing additional information not in the ms, or briefly explain why the item is not applicable/relevant for your study

Yanıtınız

5) The interventions for each group with sufficient details to allow replication, including how and when they were actually administered

5-i) Mention names, credential, affiliations of the developers, sponsors, and owners

Mention names, credential, affiliations of the developers, sponsors, and owners [6] (if authors/evaluators are owners or developer of the software, this needs to be declared in a “Conflict of interest” section or mentioned elsewhere in the manuscript).

| | 1 | 2 | 3 | 4 | 5 | |
|---|---|---|---|---|---|---|
| subitem not at all important | ○ | ○ | ○ | ○ | ○ | essential |

Does your paper address subitem 5-i?

Copy and paste relevant sections from the manuscript (include quotes in quotation marks "like this" to indicate direct quotes from your manuscript), or elaborate on this item by providing additional information not in the ms, or briefly explain why the item is not applicable/relevant for your study

Yanıtınız

5-ii) Describe the history/development process

Describe the history/development process of the application and previous formative evaluations (e.g., focus groups, usability testing), as these will have an impact on adoption/use rates and help with interpreting results.

| | 1 | 2 | 3 | 4 | 5 | |
|---|---|---|---|---|---|---|
| subitem not at all important | ○ | ○ | ○ | ○ | ○ | essential |

Does your paper address subitem 5-ii?

Copy and paste relevant sections from the manuscript (include quotes in quotation marks "like this" to indicate direct quotes from your manuscript), or elaborate on this item by providing additional information not in the ms, or briefly explain why the item is not applicable/relevant for your study

Yanıtınız

5-iii) Revisions and updating

Revisions and updating. Clearly mention the date and/or version number of the application/intervention (and comparator, if applicable) evaluated, or describe whether the intervention underwent major changes during the evaluation process, or whether the development and/or content was "frozen" during the trial. Describe dynamic components such as news feeds or changing content which may have an impact on the replicability of the intervention (for unexpected events see item 3b).

| | 1 | 2 | 3 | 4 | 5 | |
|---|---|---|---|---|---|---|
| subitem not at all important | ○ | ○ | ○ | ○ | ○ | essential |

Does your paper address subitem 5-iii?

Copy and paste relevant sections from the manuscript (include quotes in quotation marks "like this" to indicate direct quotes from your manuscript), or elaborate on this item by providing additional information not in the ms, or briefly explain why the item is not applicable/relevant for your study

Yanıtınız

5-iv) Quality assurance methods

Provide information on quality assurance methods to ensure accuracy and quality of information provided [1], if applicable.

| | 1 | 2 | 3 | 4 | 5 | |
|---|---|---|---|---|---|---|
| subitem not at all important | ○ | ○ | ○ | ○ | ○ | essential |

Does your paper address subitem 5-iv?

Copy and paste relevant sections from the manuscript (include quotes in quotation marks "like this" to indicate direct quotes from your manuscript), or elaborate on this item by providing additional information not in the ms, or briefly explain why the item is not applicable/relevant for your study

Yanıtınız

5-v) Ensure replicability by publishing the source code, and/or providing screenshots/screen-capture video, and/or providing flowcharts of the algorithms used

Ensure replicability by publishing the source code, and/or providing screenshots/screen-capture video, and/or providing flowcharts of the algorithms used. Replicability (i.e., other researchers should in principle be able to replicate the study) is a hallmark of scientific reporting.

| | 1 | 2 | 3 | 4 | 5 | |
|---|---|---|---|---|---|---|
| subitem not at all important | ○ | ○ | ○ | ○ | ○ | essential |

Does your paper address subitem 5-v?

Copy and paste relevant sections from the manuscript (include quotes in quotation marks "like this" to indicate direct quotes from your manuscript), or elaborate on this item by providing additional information not in the ms, or briefly explain why the item is not applicable/relevant for your study

Yanıtınız

5-vi) Digital preservation

Digital preservation: Provide the URL of the application, but as the intervention is likely to change or disappear over the course of the years; also make sure the intervention is archived (Internet Archive, webcitation.org, and/or publishing the source code or screenshots/videos alongside the article). As pages behind login screens cannot be archived, consider creating demo pages which are accessible without login.

| | 1 | 2 | 3 | 4 | 5 | |
|---|---|---|---|---|---|---|
| subitem not at all important | ○ | ○ | ○ | ○ | ○ | essential |

Does your paper address subitem 5-vi?

Copy and paste relevant sections from the manuscript (include quotes in quotation marks "like this" to indicate direct quotes from your manuscript), or elaborate on this item by providing additional information not in the ms, or briefly explain why the item is not applicable/relevant for your study

Yanıtınız

5-vii) Access

Access: Describe how participants accessed the application, in what setting/context, if they had to pay (or were paid) or not, whether they had to be a member of specific group. If known, describe how participants obtained “access to the platform and Internet” [1]. To ensure access for editors/reviewers/readers, consider to provide a “backdoor” login account or demo mode for reviewers/readers to explore the application (also important for archiving purposes, see vi).

| | 1 | 2 | 3 | 4 | 5 | |
|---|---|---|---|---|---|---|
| subitem not at all important | ○ | ○ | ○ | ○ | ○ | essential |

Does your paper address subitem 5-vii? *

Copy and paste relevant sections from the manuscript (include quotes in quotation marks "like this" to indicate direct quotes from your manuscript), or elaborate on this item by providing additional information not in the ms, or briefly explain why the item is not applicable/relevant for your study

"Participants in the DWM condition received the materials via WhatsApp message and then commenced the program with an Introduction Call from their assigned facilitator. In this call, the facilitators explained the DWM program, outlined their roles, discussed privacy and confidentiality, and identified participants' goals for participating in the program. This was followed by weekly lesson calls (via Zoom) during which facilitators reviewed and practiced each strategy with the participants, answered any questions, and discussed any challenges they encountered while using the materials. At the end of each call, participants and their facilitators collaboratively completed the Daily Action Plan to schedule between-session practices and to identify potential challenges and solutions to help participants stay on track." "Participants in the RAC condition were informed of their group allocation via WhatsApp message, provided with information about their upcoming assessments with the assessors, and notified that they would receive access to the DWM materials upon completing assessments. Once they completed the assessments, they were provided with a link to access the DWM booklet and audio recordings."

5-viii) Mode of delivery, features/functionalities/components of the intervention and comparator, and the theoretical framework

Describe mode of delivery, features/functionalities/components of the intervention and comparator, and the theoretical framework [6] used to design them (instructional strategy [1], behaviour change techniques, persuasive features, etc., see e.g., [7, 8] for terminology). This includes an in-depth description of the content (including where it is coming from and who developed it) [1]," whether [and how] it is tailored to individual circumstances and allows users to track their progress and receive feedback" [6]. This also includes a description of communication delivery channels and – if computer-mediated communication is a component – whether communication was synchronous or asynchronous [6]. It also includes information on presentation strategies [1], including page design principles, average amount of text on pages, presence of hyperlinks to other resources, etc. [1].

| | 1 | 2 | 3 | 4 | 5 | |
|---|---|---|---|---|---|---|
| subitem not at all important | ○ | ○ | ○ | ○ | ○ | essential |

Does your paper address subitem 5-viii? *

Copy and paste relevant sections from the manuscript (include quotes in quotation marks "like this" to indicate direct quotes from your manuscript), or elaborate on this item by providing additional information not in the ms, or briefly explain why the item is not applicable/relevant for your study

"DWM is a self-help stress management program developed by the WHO[10] to teach individuals evidence-based strategies for reducing psychological distress. The program consists of an illustrated booklet with five sections and accompanying audio recordings, introducing techniques based on Acceptance and Commitment Therapy, such as mindful attention, acceptance of difficult thoughts and feelings, engaging in valued actions, and practicing kindness toward oneself and others. In this study, the Farsi version of DWM was delivered over five weeks via weekly one-to-one calls with a trained facilitator. The original Farsi materials developed by the WHO [10] were used in this study without modification to preserve fidelity and replicability. Participants in the DWM condition received the materials via WhatsApp message and then commenced the program with an Introduction Call from their assigned facilitator. In this call, the facilitators explained the DWM program, outlined their roles, discussed privacy and confidentiality, and identified participants' goals for participating in the program. This was followed by weekly lesson calls (via Zoom) during which facilitators reviewed and practiced each strategy with the participants, answered any questions, and discussed any challenges they encountered while using the materials. At the end of each call, participants and their facilitators collaboratively completed the Daily Action Plan to schedule between-session practices and to identify potential challenges and solutions to help participants stay on track."

5-ix) Describe use parameters

Describe use parameters (e.g., intended “doses” and optimal timing for use). Clarify what instructions or recommendations were given to the user, e.g., regarding timing, frequency, heaviness of use, if any, or was the intervention used ad libitum.

| | 1 | 2 | 3 | 4 | 5 | |
|---|---|---|---|---|---|---|
| subitem not at all important | ○ | ○ | ○ | ○ | ○ | essential |

Does your paper address subitem 5-ix?

Copy and paste relevant sections from the manuscript (include quotes in quotation marks "like this" to indicate direct quotes from your manuscript), or elaborate on this item by providing additional information not in the ms, or briefly explain why the item is not applicable/relevant for your study

Yanıtınız

5-x) Clarify the level of human involvement

Clarify the level of human involvement (care providers or health professionals, also technical assistance) in the e-intervention or as co-intervention (detail number and expertise of professionals involved, if any, as well as “type of assistance offered, the timing and frequency of the support, how it is initiated, and the medium by which the assistance is delivered”. It may be necessary to distinguish between the level of human involvement required for the trial, and the level of human involvement required for a routine application outside of a RCT setting (discuss under item 21 – generalizability).

| | 1 | 2 | 3 | 4 | 5 | |
|---|---|---|---|---|---|---|
| subitem not at all important | ○ | ○ | ○ | ○ | ○ | essential |

Does your paper address subitem 5-x?

Copy and paste relevant sections from the manuscript (include quotes in quotation marks "like this" to indicate direct quotes from your manuscript), or elaborate on this item by providing additional information not in the ms, or briefly explain why the item is not applicable/relevant for your study

Yanıtınız

5-xi) Report any prompts/reminders used

Report any prompts/reminders used: Clarify if there were prompts (letters, emails, phone calls, SMS) to use the application, what triggered them, frequency etc. It may be necessary to distinguish between the level of prompts/reminders required for the trial, and the level of prompts/reminders for a routine application outside of a RCT setting (discuss under item 21 – generalizability).

| | 1 | 2 | 3 | 4 | 5 | |
|---|---|---|---|---|---|---|
| subitem not at all important | ○ | ○ | ○ | ○ | ○ | essential |

Does your paper address subitem 5-xi? *

Copy and paste relevant sections from the manuscript (include quotes in quotation marks "like this" to indicate direct quotes from your manuscript), or elaborate on this item by providing additional information not in the ms, or briefly explain why the item is not applicable/relevant for your study

Not applicable to the current study as the intervention was provided by the trained assessors weekly.

5-xii) Describe any co-interventions (incl. training/support)

Describe any co-interventions (incl. training/support): Clearly state any interventions that are provided in addition to the targeted eHealth intervention, as ehealth intervention may not be designed as stand-alone intervention. This includes training sessions and support [1]. It may be necessary to distinguish between the level of training required for the trial, and the level of training for a routine application outside of a RCT setting (discuss under item 21 – generalizability.

| | 1 | 2 | 3 | 4 | 5 | |
|---|---|---|---|---|---|---|
| subitem not at all important | ○ | ○ | ○ | ○ | ○ | essential |

Does your paper address subitem 5-xii? *

Copy and paste relevant sections from the manuscript (include quotes in quotation marks "like this" to indicate direct quotes from your manuscript), or elaborate on this item by providing additional information not in the ms, or briefly explain why the item is not applicable/relevant for your study

Not applicable to the current study as the intervention was provided by trained facilitators via Zoom.

6a) Completely defined pre-specified primary and secondary outcome measures, including how and when they were assessed

Does your paper address CONSORT subitem 6a? *

Copy and paste relevant sections from the manuscript (include quotes in quotation marks "like this" to indicate direct quotes from your manuscript), or elaborate on this item by providing additional information not in the ms, or briefly explain why the item is not applicable/relevant for your study

"The objective of this study was to evaluate the efficacy of DWM in reducing psychological distress (primary outcome), PTSD symptoms, personally identified psychological problems improving wellbeing and functioning amongst Afghan refugees living in Indonesia, as well as to evaluate the clinical significance of associated symptom change" "Participants were assessed at four-time points: baseline, mid-treatment (three weeks after randomization or upon completion of Lesson 3 in the DWM condition), post-treatment (six weeks after randomization or after completing Lesson 5 in DWM), and one-month follow-up (nine weeks after randomization or one month after completing Lesson 5 in DWM). After each assessment, participants received a $USD6 digital grocery voucher as compensation and internet allowance.

Screening measures. To determine eligibility, we used the total score of the Kessler Psychological Distress Scale (K10)[29], a 10-item measure of anxiety and depression symptoms with total scores ranging from 0 to 50. Participants were eligible to take part in the study if they reached the threshold of moderate to high psychological distress, which is represented by a cut-off score of 20 on the K10 [32]. A cut-off of 20 was selected to recruit participants with moderate to severe levels of psychological distress. This score was derived from population-based surveys that specify that a cut-off of 20 represents likelihood of having a mild mental disorder [32] Further, this approach is consistent with other research investigating the efficacy of scalable interventions that has employed an inclusion cut-off of 20 on the K10 to represent moderate-to-severe psychological distress [33].

Suicide risk was assessed with three questions adapted from the Problem Management Plus (PM+) manual[8] which assessed whether the participant (1) had serious thoughts or a plan to end their life over the past month, (2) had taken any actions to end their life over the past month, or (3) planned to end their life in the next two weeks. Participants were ineligible to take part in the current study if they reported a suicide attempt in the past month or a plan to end their life in the next two weeks.

Severe mental disorder and cognitive impairment were again assessed using the PM+ observation checklist completed by the assessors. Participants were evaluated based on their ability to understand and follow the conversation (indicative of cognitive impairment) and whether they appeared disconnected from reality or exhibited unusual behaviours (e.g., disorganized speech and confusion).

The primary outcome for this study was psychological distress, which was assessed using the K10 (α = 0.83)[29]. Secondary outcomes for this study were assessed as follows. Symptoms of posttraumatic stress disorder were measured using the PTSD Checklist-Civilian six-item version (PCL-6) [34] rated on a 4-point Likert Scale (0= not at all, 4= extremely) (α = 0.76; range: 0 to 24, with higher scores indicating higher PTSD symptoms). Perceived level of well-being was assessed with the World Health Organization (WHO) 5-item Wellbeing Index (WHO-5) [35], rated on a 6-point Likert Scale (0= at no time, 5= all of the time) (α = 0.72; range 0-25, with higher indicating better well-being (range 0-25). Functional impairment was measured with the 12-item WHODAS 2.0 [36], rated on a 5-point scale (1= no difficulty, 5= extreme difficulty (α = 0.87; range 0-60, with higher scores indicating greater functional impairment). Social functioning was assessed with nine items from the modified version of the Social Adjustment Scale-Self Report [37,38] asking for frequency and quality of interactions with family and friends and spending time on leisure time activities. The items are rated on a 5-point Likert Scale (1= not all, 5= all the time) (α = 0.65; range 9-45, with higher scores indicating greater social adjustment). Self-identified problems were measured with the Psychological Outcomes Profiles (PSYCHLOPS) [39] asking participants to generate two salient problems and rate their impact overall on a 5-

point scale (1= not all, 5= severely affected) (Spearman-Brown coefficient = 0.66; range 0-10, with higher scores indicating greater reported problems). Eight items adapted from the Harvard Trauma Questionnaire (HTQ) [40] was used to assess exposure to potentially traumatic events, rated as Yes (1) and No (0). Seven items adapted from the modified version of the Post-Migration Living Difficulties Checklist (PMLDC) [41–43] were used to measure experiences of post-displacement stressors in Indonesia with items rated on a 5-point scale (0=a small problem, 4= a very serious problem). "

6a-i) Online questionnaires: describe if they were validated for online use and apply CHERRIES items to describe how the questionnaires were designed/deployed

If outcomes were obtained through online questionnaires, describe if they were validated for online use and apply CHERRIES items to describe how the questionnaires were designed/deployed [9].

| | 1 | 2 | 3 | 4 | 5 | |
|---|---|---|---|---|---|---|
| subitem not at all important | ○ | ○ | ○ | ○ | ○ | essential |

Does your paper address subitem 6a-i?

Copy and paste relevant sections from manuscript text

Yanıtınız

6a-ii) Describe whether and how “use” (including intensity of use/dosage) was defined/measured/monitored

Describe whether and how “use” (including intensity of use/dosage) was defined/measured/monitored (logins, logfile analysis, etc.). Use/adoption metrics are important process outcomes that should be reported in any ehealth trial.

| | 1 | 2 | 3 | 4 | 5 | |
|---|---|---|---|---|---|---|
| subitem not at all important | ○ | ○ | ○ | ○ | ○ | essential |

Does your paper address subitem 6a-ii?

Copy and paste relevant sections from manuscript text

Yanıtınız

6a-iii) Describe whether, how, and when qualitative feedback from participants was obtained

Describe whether, how, and when qualitative feedback from participants was obtained (e.g., through emails, feedback forms, interviews, focus groups).

| | 1 | 2 | 3 | 4 | 5 | |
|---|---|---|---|---|---|---|
| subitem not at all important | ○ | ○ | ○ | ○ | ○ | essential |

Does your paper address subitem 6a-iii?

Copy and paste relevant sections from manuscript text

Yanıtınız

6b) Any changes to trial outcomes after the trial commenced, with reasons

Does your paper address CONSORT subitem 6b? *

Copy and paste relevant sections from the manuscript (include quotes in quotation marks "like this" to indicate direct quotes from your manuscript), or elaborate on this item by providing additional information not in the ms, or briefly explain why the item is not applicable/relevant for your study

Not relevant for the current study as no changes were made after the commencement of the study

7a) How sample size was determined

NPT: When applicable, details of whether and how the clustering by care provides or centers was addressed

7a-i) Describe whether and how expected attrition was taken into account when calculating the sample size

Describe whether and how expected attrition was taken into account when calculating the sample size.

| | 1 | 2 | 3 | 4 | 5 | |
|---|---|---|---|---|---|---|
| subitem not at all important | ○ | ○ | ○ | ○ | ○ | essential |

Does your paper address subitem 7a-i?

Copy and paste relevant sections from manuscript title (include quotes in quotation marks "like this" to indicate direct quotes from your manuscript), or elaborate on this item by providing additional information not in the ms, or briefly explain why the item is not applicable/relevant for your study

"Based on previous studies with Self Help-Plus (the group-based version of DWM)[13,15], we hypothesized a 0.40 between-groups effect size on our primary outcome (K10) and calculated that a minimum of 73 participants per condition would be required to achieve 80% power for $\alpha = 0.05$. Given that we were also interested in investigating mechanisms within the DWM condition, we required a larger sample size in this group (n = 146). Estimating at 35% attrition rate due to the mobility of the refugee population in Indonesia, a final sample size of 303 participants (202 in the DWM and 101 in the RAC condition) was planned."

7b) When applicable, explanation of any interim analyses and stopping guidelines

Does your paper address CONSORT subitem 7b? *

Copy and paste relevant sections from the manuscript (include quotes in quotation marks "like this" to indicate direct quotes from your manuscript), or elaborate on this item by providing additional information not in the ms, or briefly explain why the item is not applicable/relevant for your study

Not relevant for the current study as no interim analysis was conducted nor any stopping guidelines implemented.

8a) Method used to generate the random allocation sequence

NPT: When applicable, how care providers were allocated to each trial group

Does your paper address CONSORT subitem 8a? *

Copy and paste relevant sections from the manuscript (include quotes in quotation marks "like this" to indicate direct quotes from your manuscript), or elaborate on this item by providing additional information not in the ms, or briefly explain why the item is not applicable/relevant for your study

"Randomization was conducted by a researcher who was not involved in the delivery of the intervention. Randomization was performed using a computerized software (REDCap)30."

8b) Type of randomisation; details of any restriction (such as blocking and block size)

Does your paper address CONSORT subitem 8b? *

Copy and paste relevant sections from the manuscript (include quotes in quotation marks "like this" to indicate direct quotes from your manuscript), or elaborate on this item by providing additional information not in the ms, or briefly explain why the item is not applicable/relevant for your study

" Eligible participants were randomly assigned to DWM or the RAC condition at a 2:1 ratio. This ratio was implemented to allow for the investigation of mechanisms of action within the DWM condition (which will be detailed in future reports). Randomization was conducted by a researcher who was not involved in the delivery of the intervention. Randomization was performed using a computerized software (REDCap)30."

9) Mechanism used to implement the random allocation sequence (such as sequentially numbered containers), describing any steps taken to conceal the sequence until interventions were assigned

Does your paper address CONSORT subitem 9? *

Copy and paste relevant sections from the manuscript (include quotes in quotation marks "like this" to indicate direct quotes from your manuscript), or elaborate on this item by providing additional information not in the ms, or briefly explain why the item is not applicable/relevant for your study

"Randomization was conducted by a researcher who was not involved in the delivery of the intervention. Randomization was performed using a computerized software (REDCap)30. Assessors were masked to treatment condition allocation. Assessors were managed separately from other members of the research team and did not interact with the DWM facilitators. Participants were instructed not to share their condition allocation with the assessors to ensure objectivity of the assessments."

10) Who generated the random allocation sequence, who enrolled participants, and who assigned participants to interventions

Does your paper address CONSORT subitem 10? *

Copy and paste relevant sections from the manuscript (include quotes in quotation marks "like this" to indicate direct quotes from your manuscript), or elaborate on this item by providing additional information not in the ms, or briefly explain why the item is not applicable/relevant for your study

"Randomization was conducted by a researcher who was not involved in the delivery of the intervention". Additionally, the researcher assistant who was responsible for the trial management enrolled participants to the conditions based on their group allocations determined by the randomization process oversight by a researcher who wasnt involved in the delivery of the intervention.

11a) If done, who was blinded after assignment to interventions (for example, participants, care providers, those assessing outcomes) and how

NPT: Whether or not administering co-interventions were blinded to group assignment

### 11a-i) Specify who was blinded, and who wasn’t

Specify who was blinded, and who wasn’t. Usually, in web-based trials it is not possible to blind the participants [1, 3] (this should be clearly acknowledged), but it may be possible to blind outcome assessors, those doing data analysis or those administering co-interventions (if any).

| | 1 | 2 | 3 | 4 | 5 | |
|---|---|---|---|---|---|---|
| subitem not at all important | ○ | ○ | ○ | ○ | ○ | essential |

### Does your paper address subitem 11a-i? *

Copy and paste relevant sections from the manuscript (include quotes in quotation marks "like this" to indicate direct quotes from your manuscript), or elaborate on this item by providing additional information not in the ms, or briefly explain why the item is not applicable/relevant for your study

"Assessors were masked to treatment condition allocation. Assessors were managed separately from other members of the research team and did not interact with the DWM facilitators. Participants were instructed not to share their condition allocation with the assessors to ensure objectivity of the assessments. "

### 11a-ii) Discuss e.g., whether participants knew which intervention was the “intervention of interest” and which one was the “comparator”

Informed consent procedures (4a-ii) can create biases and certain expectations - discuss e.g., whether participants knew which intervention was the “intervention of interest” and which one was the “comparator”.

| | 1 | 2 | 3 | 4 | 5 | |
|---|---|---|---|---|---|---|
| subitem not at all important | ○ | ○ | ○ | ○ | ○ | essential |

Does your paper address subitem 11a-ii?

Copy and paste relevant sections from the manuscript (include quotes in quotation marks "like this" to indicate direct quotes from your manuscript), or elaborate on this item by providing additional information not in the ms, or briefly explain why the item is not applicable/relevant for your study

Yanıtınız

11b) If relevant, description of the similarity of interventions

(this item is usually not relevant for ehealth trials as it refers to similarity of a placebo or sham intervention to a active medication/intervention)

Does your paper address CONSORT subitem 11b? *

Copy and paste relevant sections from the manuscript (include quotes in quotation marks "like this" to indicate direct quotes from your manuscript), or elaborate on this item by providing additional information not in the ms, or briefly explain why the item is not applicable/relevant for your study

"A repeated assessment control condition was implemented in this study to evaluate the relative benefit of DWM over and above supportive contact with refugee community members. This condition was selected as (1) we were interested in investigating the efficacy of DWM in contexts where there are limited supports are available; the absence of formal services for refugees in Indonesia meant that a care-as-usual control condition was not meaningful, and (2) the substantial assessment schedule in this study (four assessments administered via Zoom by refugee community members) allows us to, at least partly, control for potential benefits of social contact on psychological distress [31]. While the repeated assessment control afforded an ecologically valid control condition that allows us to address study aims, this design means we are unable to make inferences regarding the superiority of DWM compared to other methods, nor to completely rule out that study effects were driven by supportive contact (as those in the DWM condition received a greater dosage of this)."

12a) Statistical methods used to compare groups for primary and secondary outcomes

NPT: When applicable, details of whether and how the clustering by care providers or centers was addressed

Does your paper address CONSORT subitem 12a? *

Copy and paste relevant sections from the manuscript (include quotes in quotation marks "like this" to indicate direct quotes from your manuscript), or elaborate on this item by providing additional information not in the ms, or briefly explain why the item is not applicable/relevant for your study

"For our primary analysis, we investigated the impact of DWM on the total K10 score at each timepoint in the intention-to-treat (ITT) sample. We used a linear-mixed-models approach to estimate the between-groups effect of DWM vs RAC at mid-treatment, post-treatment, and follow-up. This model had time as a fixed effect, covaried for baseline K10 score, and included participant as a random effect. We constrained the fixed effect of the intervention to be 0 and created dummy variables for time at mid-treatment, post-treatment, and follow-up (with pre-treatment being the reference group). We then included dummy-coded time x intervention interaction terms in the model for each time-point. This yielded estimates of the average between-group differences in K10 score for DWM vs RAC at each time-point, controlling for baseline scores on K10. Robust inference for fixed effects was obtained using cluster-robust (sandwich) standard errors with the CR2 small-sample correction, implemented via the clubSandwich package in R, with clustering at the participant level. Statistical tests and p-values were based on Satterthwaite-type degrees of freedom derived from the CR2 variance estimator. We next tested whether the same pattern of results was found when adjusting for covariates (age, gender, count of traumatic experiences, and time spent in Indonesia), in the per-protocol population, among follow-up completers, and for secondary outcomes using the same approach described above. We calculated effect sizes (Cohen's d) by dividing the between-groups effect by the pooled baseline standard deviation, adjusting for baseline scores on the outcome variable. We calculated Reliable Change Index (RCI) scores for the K10 following the procedure outlined by Jacobson & Traux (1991), to examine whether changes from baseline to mid-treatment, post-treatment, and one-month follow-up assessments were reliable and clinically meaningful in addition to being statistically significant. All statistical analyses were conducted in R Studio 4.4.3. "

12a-i) Imputation techniques to deal with attrition / missing values

Imputation techniques to deal with attrition / missing values: Not all participants will use the intervention/comparator as intended and attrition is typically high in ehealth trials. Specify how participants who did not use the application or dropped out from the trial were treated in the statistical analysis (a complete case analysis is strongly discouraged, and simple imputation techniques such as LOCF may also be problematic [4]).

| | 1 | 2 | 3 | 4 | 5 | |
|---|---|---|---|---|---|---|
| subitem not at all important | ○ | ○ | ○ | ○ | ○ | essential |

Does your paper address subitem 12a-i? *

Copy and paste relevant sections from the manuscript (include quotes in quotation marks "like this" to indicate direct quotes from your manuscript), or elaborate on this item by providing additional information not in the ms, or briefly explain why the item is not applicable/relevant for your study

"Per-protocol results are presented in Multimedia Appendix 6 and 7. Findings were consistent with the ITT analyses. Sensitivity analyses with participants completed the follow-up assessment found a similar pattern, except that the DWM condition no longer showed greater improvements than the RAC condition in functional impairment at post-treatment (β = -0.323, SE = 0.123, P=.085) and follow-up (β = -0.255, SE = 0.121, P=.060)."

12b) Methods for additional analyses, such as subgroup analyses and adjusted analyses

Does your paper address CONSORT subitem 12b? *

Copy and paste relevant sections from the manuscript (include quotes in quotation marks "like this" to indicate direct quotes from your manuscript), or elaborate on this item by providing additional information not in the ms, or briefly explain why the item is not applicable/relevant for your study

"The same results were obtained in the covariate-adjusted model (Table 3). Per-protocol results are presented in Multimedia Appendix 6 and 7. Findings were consistent with the ITT analyses. Sensitivity analyses with participants completed the follow-up assessment found a similar pattern, except that the DWM condition no longer showed greater improvements than the RAC condition in functional impairment at post-treatment (β = -0.323, SE = 0.123, P=.085) and follow-up (β = -0.255, SE = 0.121, P=.060). "

X26) REB/IRB Approval and Ethical Considerations [recommended as subheading under "Methods"] (not a CONSORT item)

X26-i) Comment on ethics committee approval

| | 1 | 2 | 3 | 4 | 5 | |
|---|---|---|---|---|---|---|
| subitem not at all important | ○ | ○ | ○ | ○ | ○ | essential |

Does your paper address subitem X26-i?

Copy and paste relevant sections from the manuscript (include quotes in quotation marks "like this" to indicate direct quotes from your manuscript), or elaborate on this item by providing additional information not in the ms, or briefly explain why the item is not applicable/relevant for your study

Yanıtınız

x26-ii) Outline informed consent procedures

Outline informed consent procedures e.g., if consent was obtained offline or online (how? Checkbox, etc.?), and what information was provided (see 4a-ii). See [6] for some items to be included in informed consent documents.

| | 1 | 2 | 3 | 4 | 5 | |
|---|---|---|---|---|---|---|
| subitem not at all important | ○ | ○ | ○ | ○ | ○ | essential |

Does your paper address subitem X26-ii?

Copy and paste relevant sections from the manuscript (include quotes in quotation marks "like this" to indicate direct quotes from your manuscript), or elaborate on this item by providing additional information not in the ms, or briefly explain why the item is not applicable/relevant for your study

Yanıtınız

X26-iii) Safety and security procedures

Safety and security procedures, incl. privacy considerations, and any steps taken to reduce the likelihood or detection of harm (e.g., education and training, availability of a hotline)

| | 1 | 2 | 3 | 4 | 5 | |
|---|---|---|---|---|---|---|
| subitem not at all important | ○ | ○ | ○ | ○ | ○ | essential |

Does your paper address subitem X26-iii?

Copy and paste relevant sections from the manuscript (include quotes in quotation marks "like this" to indicate direct quotes from your manuscript), or elaborate on this item by providing additional information not in the ms, or briefly explain why the item is not applicable/relevant for your study

Yanıtınız

RESULTS

13a) For each group, the numbers of participants who were randomly assigned, received intended treatment, and were analysed for the primary outcome

NPT: The number of care providers or centers performing the intervention in each group and the number of patients treated by each care provider in each center

Does your paper address CONSORT subitem 13a? *

Copy and paste relevant sections from the manuscript (include quotes in quotation marks "like this" to indicate direct quotes from your manuscript), or elaborate on this item by providing additional information not in the ms, or briefly explain why the item is not applicable/relevant for your study

"Participant recruitment was conducted from June 2024 to March 2025. One-month follow-up assessments were completed in June 2025. A total of 547 potentially eligible participants completed the screening assessment, of whom 375 proceeded to the baseline assessment. Out of these 375 participants, 72 participants were excluded because of limited Farsi proficiency (N=20), self-withdrawal from the study (N=17), decreased K10 scores since the screening call which rendered them ineligible for participation (N=12), suicide risk (N=10) and other reasons (e.g., currently receiving psychological treatment, lack of private space in which to do calls, no access to smartphone or internet; N = 14). Out of the 375 people who completed baseline assessments, 303 were randomized to the DWM or RAC condition. The flow of the participants is provided in the CONSORT Flow (Figure 1). The retention rate from baseline to follow-up assessment was 87.79%. There were no significant differences in the distributions of participants lost to follow-up between the study conditions at any assessment point (Multimedia Appendix 4). The comparison between the participants who were lost to follow-up and retained on key characteristics is presented in Multimedia Appendix 5. The majority of the participants in the DWM condition completed all five Lessons (N=180, 89.11%). No serious adverse event was reported."

13b) For each group, losses and exclusions after randomisation, together with reasons

Does your paper address CONSORT subitem 13b? (NOTE: Preferably, this is shown in a CONSORT flow diagram) *

Copy and paste relevant sections from the manuscript (include quotes in quotation marks "like this" to indicate direct quotes from your manuscript), or elaborate on this item by providing additional information not in the ms, or briefly explain why the item is not applicable/relevant for your study

This is shown in the CONSORT Flow Diagram in detail.

13b-i) Attrition diagram

Strongly recommended: An attrition diagram (e.g., proportion of participants still logging in or using the intervention/comparator in each group plotted over time, similar to a survival curve) or other figures or tables demonstrating usage/dose/engagement.

| | 1 | 2 | 3 | 4 | 5 | |
|---|---|---|---|---|---|---|
| subitem not at all important | ○ | ○ | ○ | ○ | ○ | essential |

Does your paper address subitem 13b-i?

Copy and paste relevant sections from the manuscript or cite the figure number if applicable (include quotes in quotation marks "like this" to indicate direct quotes from your manuscript), or elaborate on this item by providing additional information not in the ms, or briefly explain why the item is not applicable/relevant for your study

Yanıtınız

14a) Dates defining the periods of recruitment and follow-up

Does your paper address CONSORT subitem 14a? *

Copy and paste relevant sections from the manuscript (include quotes in quotation marks "like this" to indicate direct quotes from your manuscript), or elaborate on this item by providing additional information not in the ms, or briefly explain why the item is not applicable/relevant for your study

"Participant recruitment was conducted from June 2024 to March 2025. One-month follow-up assessments were completed in June 2025."

14a-i) Indicate if critical "secular events" fell into the study period

Indicate if critical "secular events" fell into the study period, e.g., significant changes in Internet resources available or "changes in computer hardware or Internet delivery resources"

| | 1 | 2 | 3 | 4 | 5 | |
|---|---|---|---|---|---|---|
| subitem not at all important | ○ | ○ | ○ | ○ | ○ | essential |

Does your paper address subitem 14a-i?

Copy and paste relevant sections from the manuscript (include quotes in quotation marks "like this" to indicate direct quotes from your manuscript), or elaborate on this item by providing additional information not in the ms, or briefly explain why the item is not applicable/relevant for your study

Yanıtınız

14b) Why the trial ended or was stopped (early)

Does your paper address CONSORT subitem 14b? *

Copy and paste relevant sections from the manuscript (include quotes in quotation marks "like this" to indicate direct quotes from your manuscript), or elaborate on this item by providing additional information not in the ms, or briefly explain why the item is not applicable/relevant for your study

Not relevant because the intervention didn't end prematurely.

15) A table showing baseline demographic and clinical characteristics for each group

NPT: When applicable, a description of care providers (case volume, qualification, expertise, etc.) and centers (volume) in each group

Does your paper address CONSORT subitem 15? *

Copy and paste relevant sections from the manuscript (include quotes in quotation marks "like this" to indicate direct quotes from your manuscript), or elaborate on this item by providing additional information not in the ms, or briefly explain why the item is not applicable/relevant for your study

This is given in Table 1 in detail.

15-i) Report demographics associated with digital divide issues

In ehealth trials it is particularly important to report demographics associated with digital divide issues, such as age, education, gender, social-economic status, computer/Internet/ehealth literacy of the participants, if known.

| | 1 | 2 | 3 | 4 | 5 | |
|---|---|---|---|---|---|---|
| subitem not at all important | ○ | ○ | ○ | ○ | ○ | essential |

Does your paper address subitem 15-i? *

Copy and paste relevant sections from the manuscript (include quotes in quotation marks "like this" to indicate direct quotes from your manuscript), or elaborate on this item by providing additional information not in the ms, or briefly explain why the item is not applicable/relevant for your study

The demographics of the participants are given in Table 1. No information is available regarding ehealth literacy of the participants.

16) For each group, number of participants (denominator) included in each analysis and whether the analysis was by original assigned groups

16-i) Report multiple “denominators” and provide definitions

Report multiple “denominators” and provide definitions: Report N’s (and effect sizes) “across a range of study participation [and use] thresholds” [1], e.g., N exposed, N consented, N used more than x times, N used more than y weeks, N participants “used” the intervention/comparator at specific pre-defined time points of interest (in absolute and relative numbers per group). Always clearly define “use” of the intervention.

| | 1 | 2 | 3 | 4 | 5 | |
|---|---|---|---|---|---|---|
| subitem not at all important | ○ | ○ | ○ | ○ | ○ | essential |

Does your paper address subitem 16-i? *

Copy and paste relevant sections from the manuscript (include quotes in quotation marks "like this" to indicate direct quotes from your manuscript), or elaborate on this item by providing additional information not in the ms, or briefly explain why the item is not applicable/relevant for your study

"The results of intent-to-treat linear mixed models investigating differences in DWM and the RAC condition on primary and secondary outcome measures are presented in Table 2. Compared to the RAC, DWM led to significantly greater improvements in psychological distress (primary outcome; β = -0.563, SE = 0.124, P<.001, d = -0.563 at post-treatment and β = -0.447, SE = 0.140, P=.002, d = -0.447 at follow-up), as well as PTSD symptoms (β = -0.471, SE = 0.115, P<.001, d = -0.471 at post-treatment, β = -0.406, SE = 0.126, P=.002, d = -0.406 at follow-up), wellbeing (β = 0.601, SE = 0.144, P<.001, d = 0.601 at post-treatment, β = 0.324, SE = 0.152, P=.035, d = 0.324 at follow-up), functional impairment (β = -0.216, SE = 0.109, P=.048, d = -0.226 at post-treatment, β = -0.226, SE = 0.114, P=.049, d = -0.216 at follow-up), social functioning (β = 0.339, SE = 0.119, P =.005, d = 0.339 at post-treatment, β = 0.255, SE = 0.118, P=.033, d = 0.255 at follow-up) and personally-identified psychological problems (β = -0.401, SE = 0.137 P=.003, d = -0.401 at post-treatment, β = -0.321, SE = 0.152, P=.036, d = -0.321 at follow-up). Notably, the RAC condition also showed significant decreases in psychological distress, PTSD symptoms, wellbeing, functional impairment, and social functioning at follow-up. The same results were obtained in the covariate-adjusted model (Table 3). Per-protocol results are presented in Multimedia Appendix 6 and 7. Findings were consistent with the ITT analyses. Sensitivity analyses with participants completed the follow-up assessment found a similar pattern, except that the DWM condition no longer showed greater improvements than the RAC condition in functional impairment at post-treatment (β = -0.323, SE = 0.123, P=.085) and follow-up (β = -0.255, SE = 0.121, P=.060). "

16-ii) Primary analysis should be intent-to-treat

Primary analysis should be intent-to-treat, secondary analyses could include comparing only "users", with the appropriate caveats that this is no longer a randomized sample (see 18-i).

| | 1 | 2 | 3 | 4 | 5 | |
|---|---|---|---|---|---|---|
| subitem not at all important | ○ | ○ | ○ | ○ | ◉ | essential |

Seçimi temizle

Does your paper address subitem 16-ii?

Copy and paste relevant sections from the manuscript (include quotes in quotation marks "like this" to indicate direct quotes from your manuscript), or elaborate on this item by providing additional information not in the ms, or briefly explain why the item is not applicable/relevant for your study

"The results of intent-to-treat linear mixed models investigating differences in DWM and the RAC condition on primary and secondary outcome measures are presented in Table 2. Compared to the RAC, DWM led to significantly greater improvements in psychological distress (primary outcome; β = -0.563, SE = 0.124, P<.001, d = -0.563 at post-treatment and β = -0.447, SE = 0.140, P=.002, d = -0.447 at follow-up), as well as PTSD symptoms (β = -0.471, SE = 0.115, P<.001, d = -0.471 at post-treatment, β = -0.406, SE = 0.126, P=.002, d = -0.406 at follow-up), wellbeing (β = 0.601, SE = 0.144, P<.001, d = 0.601 at post-treatment, β = 0.324, SE = 0.152, P=.035, d = 0.324 at follow-up), functional impairment (β = -0.216, SE = 0.109, P=.048, d = -0.226 at post-treatment, β = -0.226, SE = 0.114, P=.049, d = -0.216 at follow-up), social functioning (β = 0.339, SE = 0.119, P =.005, d = 0.339 at post-treatment, β = 0.255, SE = 0.118, P=.033, d = 0.255 at follow-up) and personally-identified psychological problems (β = -0.401, SE = 0.137 P=.003, d = -0.401 at post-treatment, β = -0.321, SE = 0.152, P=.036, d = -0.321 at follow-up). Notably, the RAC condition also showed significant decreases in psychological distress, PTSD symptoms, wellbeing, functional impairment, and social functioning at follow-up. The same results were obtained in the covariate-adjusted model (Table 3). Per-protocol results are presented in Multimedia Appendix 6 and 7. Findings were consistent with the ITT analyses. Sensitivity analyses with participants completed the follow-up assessment found a similar pattern, except that the DWM condition no longer showed greater improvements than the RAC condition in functional impairment at post-treatment (β = -0.323, SE = 0.123, P=.085) and follow-up (β = -0.255, SE = 0.121, P=.060). "

17a) For each primary and secondary outcome, results for each group, and the estimated effect size and its precision (such as 95% confidence interval)

Does your paper address CONSORT subitem 17a? *

Copy and paste relevant sections from the manuscript (include quotes in quotation marks "like this" to indicate direct quotes from your manuscript), or elaborate on this item by providing additional information not in the ms, or briefly explain why the item is not applicable/relevant for your study

"Compared to the RAC, DWM led to significantly greater improvements in psychological distress (primary outcome; β = -0.563, SE = 0.124, P<.001, d = -0.563 at post-treatment and β = -0.447, SE = 0.140, P=.002, d = -0.447 at follow-up), as well as PTSD symptoms (β = -0.471, SE = 0.115, P<.001, d = -0.471 at post-treatment, β = -0.406, SE = 0.126, P=.002, d = -0.406 at follow-up), wellbeing (β = 0.601, SE = 0.144, P<.001, d = 0.601 at post-treatment, β = 0.324, SE = 0.152, P=.035, d = 0.324 at follow-up), functional impairment (β = -0.216, SE = 0.109, P=.048, d = -0.226 at post-treatment, β = -0.226, SE = 0.114, P=.049, d = -0.216 at follow-up), social functioning (β = 0.339, SE = 0.119, P =.005, d = 0.339 at post-treatment, β = 0.255, SE = 0.118, P=.033, d = 0.255 at follow-up) and personally-identified psychological problems (β = -0.401, SE = 0.137 P=.003, d = -0.401 at post-treatment, β = -0.321, SE = 0.152, P=.036, d = -0.321 at follow-up). Notably, the RAC condition also showed significant decreases in psychological distress, PTSD symptoms, wellbeing, functional impairment, and social functioning at follow-up. The same results were obtained in the covariate-adjusted model (Table 3)."

17a-i) Presentation of process outcomes such as metrics of use and intensity of use

In addition to primary/secondary (clinical) outcomes, the presentation of process outcomes such as metrics of use and intensity of use (dose, exposure) and their operational definitions is critical. This does not only refer to metrics of attrition (13-b) (often a binary variable), but also to more continuous exposure metrics such as “average session length”. These must be accompanied by a technical description how a metric like a “session” is defined (e.g., timeout after idle time) [1] (report under item 6a).

| | 1 | 2 | 3 | 4 | 5 | |
|---|---|---|---|---|---|---|
| subitem not at all important | ○ | ○ | ○ | ○ | ○ | essential |

Does your paper address subitem 17a-i?

Copy and paste relevant sections from the manuscript (include quotes in quotation marks "like this" to indicate direct quotes from your manuscript), or elaborate on this item by providing additional information not in the ms, or briefly explain why the item is not applicable/relevant for your study

Yanıtınız

17b) For binary outcomes, presentation of both absolute and relative effect sizes is recommended

Does your paper address CONSORT subitem 17b? *

Copy and paste relevant sections from the manuscript (include quotes in quotation marks "like this" to indicate direct quotes from your manuscript), or elaborate on this item by providing additional information not in the ms, or briefly explain why the item is not applicable/relevant for your study

No binary outcome was used in the current study.

18) Results of any other analyses performed, including subgroup analyses and adjusted analyses, distinguishing pre-specified from exploratory

Does your paper address CONSORT subitem 18? *

Copy and paste relevant sections from the manuscript (include quotes in quotation marks "like this" to indicate direct quotes from your manuscript), or elaborate on this item by providing additional information not in the ms, or briefly explain why the item is not applicable/relevant for your study

"The same results were obtained in the covariate-adjusted model (Table 3). Per-protocol results are presented in Multimedia Appendix 6 and 7. Findings were consistent with the ITT analyses. Sensitivity analyses with participants completed the follow-up assessment found a similar pattern, except that the DWM condition no longer showed greater improvements than the RAC condition in functional impairment at post-treatment (β = -0.323, SE = 0.123, P=.085) and follow-up (β = -0.255, SE = 0.121, P=.060). At post-treatment and follow-up assessments, the DWM condition had more participants who recovered or showed reliable improvement in their K10 symptoms compared to the RAC condition. Similarly, the proportion of those who deteriorated was lower in the DWM condition than in the RAC condition at each assessment (Table 4). "

18-i) Subgroup analysis of comparing only users

A subgroup analysis of comparing only users is not uncommon in ehealth trials, but if done, it must be stressed that this is a self-selected sample and no longer an unbiased sample from a randomized trial (see 16-iii).

| | 1 | 2 | 3 | 4 | 5 | |
|---|---|---|---|---|---|---|
| subitem not at all important | ○ | ○ | ○ | ○ | ○ | essential |

Does your paper address subitem 18-i?

Copy and paste relevant sections from the manuscript (include quotes in quotation marks "like this" to indicate direct quotes from your manuscript), or elaborate on this item by providing additional information not in the ms, or briefly explain why the item is not applicable/relevant for your study

Yanıtınız

19) All important harms or unintended effects in each group

(for specific guidance see CONSORT for harms)

Does your paper address CONSORT subitem 19? *

Copy and paste relevant sections from the manuscript (include quotes in quotation marks "like this" to indicate direct quotes from your manuscript), or elaborate on this item by providing additional information not in the ms, or briefly explain why the item is not applicable/relevant for your study

"No serious adverse event was reported."

19-i) Include privacy breaches, technical problems

Include privacy breaches, technical problems. This does not only include physical "harm" to participants, but also incidents such as perceived or real privacy breaches [1], technical problems, and other unexpected/unintended incidents. "Unintended effects" also includes unintended positive effects [2].

| | 1 | 2 | 3 | 4 | 5 | |
|---|---|---|---|---|---|---|
| subitem not at all important | ○ | ○ | ○ | ○ | ○ | essential |

Does your paper address subitem 19-i?

Copy and paste relevant sections from the manuscript (include quotes in quotation marks "like this" to indicate direct quotes from your manuscript), or elaborate on this item by providing additional information not in the ms, or briefly explain why the item is not applicable/relevant for your study

Yanıtınız

19-ii) Include qualitative feedback from participants or observations from staff/researchers

Include qualitative feedback from participants or observations from staff/researchers, if available, on strengths and shortcomings of the application, especially if they point to unintended/unexpected effects or uses. This includes (if available) reasons for why people did or did not use the application as intended by the developers.

| | 1 | 2 | 3 | 4 | 5 | |
|---|---|---|---|---|---|---|
| subitem not at all important | ○ | ○ | ○ | ○ | ○ | essential |

Does your paper address subitem 19-ii?

Copy and paste relevant sections from the manuscript (include quotes in quotation marks "like this" to indicate direct quotes from your manuscript), or elaborate on this item by providing additional information not in the ms, or briefly explain why the item is not applicable/relevant for your study

Yanıtınız

DISCUSSION

22) Interpretation consistent with results, balancing benefits and harms, and considering other relevant evidence

NPT: In addition, take into account the choice of the comparator, lack of or partial blinding, and unequal expertise of care providers or centers in each group

22-i) Restate study questions and summarize the answers suggested by the data, starting with primary outcomes and process outcomes (use)

Restate study questions and summarize the answers suggested by the data, starting with primary outcomes and process outcomes (use).

| | 1 | 2 | 3 | 4 | 5 | |
|---|---|---|---|---|---|---|
| subitem not at all important | ○ | ○ | ○ | ○ | ○ | essential |

Does your paper address subitem 22-i? *

Copy and paste relevant sections from the manuscript (include quotes in quotation marks "like this" to indicate direct quotes from your manuscript), or elaborate on this item by providing additional information not in the ms, or briefly explain why the item is not applicable/relevant for your study

"We found that DWM was effective in reducing psychological distress and PTSD symptoms, as well as improving wellbeing and overall daily functioning, in refugees living in a transit country." "The clinical significance of these results is reflected in the finding that, compared to the repeated assessment group, participants who received DWM showed significantly greater rates of recovery (26% vs 10%) and improvement (28% vs 20%) at the follow-up assessment as measured by the Reliable Change Index. A similar pattern of findings was also observed at post-treatment."

22-ii) Highlight unanswered new questions, suggest future research

Highlight unanswered new questions, suggest future research.

| | 1 | 2 | 3 | 4 | 5 | |
|---|---|---|---|---|---|---|
| subitem not at all important | ○ | ○ | ○ | ○ | ○ | essential |

Does your paper address subitem 22-ii?

Copy and paste relevant sections from the manuscript (include quotes in quotation marks "like this" to indicate direct quotes from your manuscript), or elaborate on this item by providing additional information not in the ms, or briefly explain why the item is not applicable/relevant for your study

Yanıtınız

20) Trial limitations, addressing sources of potential bias, imprecision, and, if relevant, multiplicity of analyses

20-i) Typical limitations in ehealth trials

Typical limitations in ehealth trials: Participants in ehealth trials are rarely blinded. Ehealth trials often look at a multiplicity of outcomes, increasing risk for a Type I error. Discuss biases due to non-use of the intervention/usability issues, biases through informed consent procedures, unexpected events.

| | 1 | 2 | 3 | 4 | 5 | |
|---|---|---|---|---|---|---|
| subitem not at all important | ○ | ○ | ○ | ○ | ○ | essential |

Does your paper address subitem 20-i? *

Copy and paste relevant sections from the manuscript (include quotes in quotation marks "like this" to indicate direct quotes from your manuscript), or elaborate on this item by providing additional information not in the ms, or briefly explain why the item is not applicable/relevant for your study

"We note a number of limitations. First, the study relied on a relatively short follow-up assessment period (1 month); future research testing the efficacy of DWM should evaluate its efficacy in the longer-term. Second, the study lacked active control condition, and as a result we cannot exclude the possible role of non-specific factors such as facilitator support, contributing to the effects of DWM. Third, we note that a number of the assessment measures have not been validated with the cultural groups who participated in the study. Finally, digital access requirements may have introduced selection bias."

21) Generalisability (external validity, applicability) of the trial findings

NPT: External validity of the trial findings according to the intervention, comparators, patients, and care providers or centers involved in the trial

21-i) Generalizability to other populations

Generalizability to other populations: In particular, discuss generalizability to a general Internet population, outside of a RCT setting, and general patient population, including applicability of the study results for other organizations

| | 1 | 2 | 3 | 4 | 5 | |
|---|---|---|---|---|---|---|
| subitem not at all important | ○ | ○ | ○ | ○ | ○ | essential |

Does your paper address subitem 21-i?

Copy and paste relevant sections from the manuscript (include quotes in quotation marks "like this" to indicate direct quotes from your manuscript), or elaborate on this item by providing additional information not in the ms, or briefly explain why the item is not applicable/relevant for your study

Yanıtınız

21-ii) Discuss if there were elements in the RCT that would be different in a routine application setting

Discuss if there were elements in the RCT that would be different in a routine application setting (e.g., prompts/reminders, more human involvement, training sessions or other co-interventions) and what impact the omission of these elements could have on use, adoption, or outcomes if the intervention is applied outside of a RCT setting.

| | 1 | 2 | 3 | 4 | 5 | |
|---|---|---|---|---|---|---|
| subitem not at all important | ○ | ○ | ○ | ○ | ○ | essential |

Does your paper address subitem 21-ii?

Copy and paste relevant sections from the manuscript (include quotes in quotation marks "like this" to indicate direct quotes from your manuscript), or elaborate on this item by providing additional information not in the ms, or briefly explain why the item is not applicable/relevant for your study

Yanıtınız

OTHER INFORMATION

23) Registration number and name of trial registry

Does your paper address CONSORT subitem 23? *

Copy and paste relevant sections from the manuscript (include quotes in quotation marks "like this" to indicate direct quotes from your manuscript), or elaborate on this item by providing additional information not in the ms, or briefly explain why the item is not applicable/relevant for your study

"The trial protocol (Multimedia Appendix 1) was prospectively registered at the Australian and New Zealand Clinical Trials Registry (ACTRN12624000609550)"

24) Where the full trial protocol can be accessed, if available

Does your paper address CONSORT subitem 24? *

Cite a Multimedia Appendix, other reference, or copy and paste relevant sections from the manuscript (include quotes in quotation marks "like this" to indicate direct quotes from your manuscript), or elaborate on this item by providing additional information not in the ms, or briefly explain why the item is not applicable/relevant for your study

"The trial protocol (Multimedia Appendix 1) was prospectively registered at the Australian and New Zealand Clinical Trials Registry (ACTRN12624000609550)"

25) Sources of funding and other support (such as supply of drugs), role of funders

Does your paper address CONSORT subitem 25? *

Copy and paste relevant sections from the manuscript (include quotes in quotation marks "like this" to indicate direct quotes from your manuscript), or elaborate on this item by providing additional information not in the ms, or briefly explain why the item is not applicable/relevant for your study

"The current study was supported by an Australian Research Council Linkage Grant (LP230100426). AN was supported by an Australian National Health and Medical Research Council Investigator Leadership Grant (2018104). PS was supported by an MQ: Transforming Mental Health Postdoctoral Scholarship (MPSIP\15)."

X27) Conflicts of Interest (not a CONSORT item)

X27-i) State the relation of the study team towards the system being evaluated

In addition to the usual declaration of interests (financial or otherwise), also state the relation of the study team towards the system being evaluated, i.e., state if the authors/evaluators are distinct from or identical with the developers/sponsors of the intervention.

| | 1 | 2 | 3 | 4 | 5 | |
|---|---|---|---|---|---|---|
| subitem not at all important | ○ | ○ | ○ | ○ | ○ | essential |

Does your paper address subitem X27-i?

Copy and paste relevant sections from the manuscript (include quotes in quotation marks "like this" to indicate direct quotes from your manuscript), or elaborate on this item by providing additional information not in the ms, or briefly explain why the item is not applicable/relevant for your study

Yanıtınız

About the CONSORT EHEALTH checklist

As a result of using this checklist, did you make changes in your manuscript? *

- ○ yes, major changes
- ◉ yes, minor changes
- ○ no

What were the most important changes you made as a result of using this checklist?

Yanıtınız

How much time did you spend on going through the checklist INCLUDING making changes in your manuscript *

It is around two hours in total- filling out the checklist (only mandatory items) and changes

As a result of using this checklist, do you think your manuscript has improved? *

- (x) yes
- ( ) no
- ( ) Diğer:

Would you like to become involved in the CONSORT EHEALTH group?

This would involve for example becoming involved in participating in a workshop and writing an "Explanation and Elaboration" document

- ( ) yes
- (x) no
- ( ) Diğer:

Seçimi temizle

Any other comments or questions on CONSORT EHEALTH

Yanıtınız

STOP - Save this form as PDF before you click submit

To generate a record that you filled in this form, we recommend to generate a PDF of this page (on a Mac, simply select "print" and then select "print as PDF") before you submit it.

When you submit your (revised) paper to JMIR, please upload the PDF as supplementary file.

Don't worry if some text in the textboxes is cut off, as we still have the complete information in our database. Thank you!

Final step: Click submit !

Click submit so we have your answers in our database!

Gönder Formu temizle

Google Formlar üzerinden asla şifre göndermeyin.

Bu içerik Google tarafından oluşturulmamış veya onaylanmamıştır. - İletişim formu sahibi - Hizmet Şartları - Gizlilik Politikası

Bu form şüpheli mi görünüyor? Rapor

Google Formlar
